# Supplementary material for: A randomized, double-blind, placebo-controlled phase IIa trial of efruxifermin for patients with compensated NASH cirrhosis
Source: JHEP Rep. 2022 Aug 23;5(1):100563. doi: 10.1016/j.jhepr.2022.100563 (PMC9832280; doi:10.1016/j.jhepr.2022.100563)
Supplement: Multimedia component 1 [file mmc1.pdf]

ARTICLE IN PRESS

Research article

JHEP|Reports

A randomized, double-blind, placebo-controlled phase IIa trial of efruxifermin for patients with compensated NASH cirrhosis

Stephen A. Harrison,[1] Peter J. Ruane,[2] Bradley Freilich,[3] Guy Neff,[4] Rashmee Patil,[5] Cynthia Behling,[6] Chen Hu,[7] Reshma Shringarpure,[8] Brittany de Temple,[8] Erica Fong,[8] Erik J. Tillman,[8] Timothy Rolph,[8] Andrew Cheng,[8] Kitty Yale[8,*]

[1]*Pinnacle Clinical Research, San Antonio, TX, United States;* [2]*Ruane Clinical Research Group Inc., Los Angeles, CA, United States;* [3]*Kansas City Research Institute, Kansas City, MO, United States;* [4]*Covenant Metabolic Specialists, LLC, Sarasota, FL, United States;* [5]*South Texas Research Institute, Edinburg, TX, United States;* [6]*University of California San Diego, CA, United States;* [7]*MedPace, INC, Cincinnati, OH, United States;* [8]*Akero Therapeutics, South San Francisco, CA, United States*

JHEP Reports **2022.** https://doi.org/10.1016/j.jhepr.2022.100563

Background & Aims: Efruxifermin has shown clinical efficacy in patients with non-alcoholic steatohepatitis (NASH) and F1–F3 fibrosis. The primary objective of the BALANCED Cohort C was to assess the safety and tolerability of efruxifermin in patients with compensated NASH cirrhosis.

Methods: Patients with NASH and stage 4 fibrosis (n = 30) were randomized 2:1 to receive efruxifermin 50 mg (n = 20) or placebo (n = 10) once weekly for 16 weeks. The primary endpoint was safety and tolerability of efruxifermin. Secondary and exploratory endpoints included evaluation of non-invasive markers of liver injury and fibrosis, glucose and lipid metabolism, and changes in histology in a subset of patients who consented to liver biopsy.

Results: Efruxifermin was safe and well-tolerated; most adverse events (AEs) were grade 1 (n = 7, 23.3%) or grade 2 (n = 19, 63.3%). The most frequent AEs were gastrointestinal, including transient, mild to moderate diarrhea, and/or nausea. Significant improvements were noted in key markers of liver injury (alanine aminotransferase) and glucose and lipid metabolism. Sixteen-week treatment with efruxifermin was associated with significant reductions in non-invasive markers of fibrosis including Pro-C3 (least squares mean change from baseline [LSMCFB] −9 μg/L efruxifermin *vs.* −3.4 μg/L placebo; $p = 0.0130$) and ELF score (−0.4 efruxifermin *vs.* +0.4 placebo; $p = 0.0036$), with a trend towards reduced liver stiffness (LSMCFB −5.7 kPa efruxifermin *vs.* −1.1 kPa placebo; n.s.). Of 12 efruxifermin-treated patients with liver biopsy after 16 weeks, 4 (33%) achieved fibrosis improvement of at least one stage without worsening of NASH, while an additional 3 (25%) achieved resolution of NASH, compared to 0 of 5 placebo-treated patients.

Conclusions: Efruxifermin appeared safe and well-tolerated with encouraging improvements in markers of liver injury, fibrosis, and glucose and lipid metabolism following 16 weeks of treatment, warranting confirmation in larger and longer term studies.

Lay summary: Cirrhosis resulting from non-alcoholic steatohepatitis (NASH), the progressive form of non-alcoholic fatty liver disease, represents a major unmet medical need. Currently there are no approved drugs for the treatment of NASH. This proof-of-concept randomized, double-blind clinical trial demonstrated the potential therapeutic benefit of efruxifermin treatment compared to placebo in patients with cirrhosis due to NASH.

Clinical Trial Number: NCT03976401

© 2022 The Author(s). Published by Elsevier B.V. on behalf of European Association for the Study of the Liver (EASL). This is an open access article under the CC BY license (http://creativecommons.org/licenses/by/4.0/).

Introduction

Non-alcoholic steatohepatitis (NASH), the most severe and progressive form of non-alcoholic fatty liver disease (NAFLD), is characterized by abnormal accumulation of fat in the liver (steatosis), hepatocellular ballooning, and inflammation.[1–3] The global prevalence of NAFLD is increasing with recent estimates ranging from 25% to 30% worldwide, with 3% to 5% progressing to NASH with advanced fibrosis or cirrhosis.[4–6]

Fibrosis stage is the strongest predictor of adverse clinical outcomes in NASH.[7–11] In a *post hoc* analysis of phase III interventional studies, an improvement of ≥1 stage from F4 to ≤F3 was associated with a reduction in incidence of liver-related outcomes from 7.2% to 1.1%, corresponding to a greater than 80% reduction in liver-related complications.[12]

In the most advanced stages, NASH may present as cryptogenic cirrhosis or burned-out NASH,[13] which is associated with higher probability of liver failure, hepatocellular carcinoma[14] and/or major adverse cardiac events.[7] NASH is among the leading causes of liver transplantation,[15] a particularly acute need for

Keywords: FGF21; non-alcoholic steatohepatitis/NASH; cirrhosis; clinical trial; liver disease; efruxifermin; nonalcoholic fatty liver disease/NAFLD; histopathology.
Received 25 June 2022; accepted 3 August 2022; available online XXX
* Corresponding author. Address: 601 Gateway Blvd, Suite 350, South San Francisco, CA 94080, United States; Tel.: 415 823 7554
E-mail address: kyale@akerotx.com (K. Yale).

ELSEVIER

EASL The Home of Hepatology

patients with cirrhosis,[9] who have an estimated transplant-free survival rate of approximately 60% over 5 years.[8]

With no approved drugs for treating NASH there is a clear unmet medical need to slow, halt or reverse progression of disease, particularly for patients with cirrhosis.[16] Optimal therapy would improve liver health (steatosis, inflammation, and fibrosis) while restoring whole-body metabolic health by reducing prevalent comorbidities of dyslipidemia, impaired glycemic control, obesity, and hyperuricemia. To date, potential drug candidates have tended to target either the underlying metabolic dysfunction of the liver, or the downstream consequences of hepatocyte stress and injury, inflammation, and fibrosis.[17]

Fibroblast growth factor 21 (FGF21) analogues have emerged as a promising therapeutic target possessing many of the characteristics of an ideal NASH therapy. FGF21 not only addresses the underlying disease driver (*i.e.*, excessive accumulation of fat in the liver), but also the downstream sequelae of inflammation and fibrosis.[18–25] FGF21 appears to act directly and indirectly to reduce pro-fibrogenic signaling.[26] Acting indirectly, FGF21 reduces liver fat and lipotoxicity while simultaneously protecting against cell stress and activation of apoptosis, in turn suppressing activation of Kupffer cells and hepatic stellate cells.[20,21,24,27] Acting directly, FGF21 appears to inhibit activation of hepatic stellate cells into collagen-secreting myofibroblasts.[26] An increasing number of FGF21 analogues are under clinical evaluation with differences evident in their therapeutic profiles. Variation in the extent of stimulation of FGF21's cognate receptors (FGFR1c, FGFR2c, FGFR3c) in the two tissues contributing most to the accumulation of fat in the liver, *i.e.* adipose tissue (via FGFR1c) or liver (via FGFR2c/3c), may at least partly explain these differences.[26] For example, induction of maximal adiponectin response downstream of FGFR1c signaling in adipose tissue appears to be weaker for the PEGylated FGF21 analogue pegbelfermin,[28] than reported for other FGF21 analogues at comparable or lower moles of FGF21.[29] Such differences may in part be attributable to lower exposure of PEGylated analogues in peripheral adipose tissue than the liver, or the differences in half-life of pegbelfermin[30] and Fc-fusion FGF21 proteins.[29]

Efruxifermin is a long-acting FGF21 analogue comprised of a modified sequence of native human FGF21 fused to the Fc region of human IgG1. The mutations in the FGF21 moiety specific to efruxifermin increase half-life in humans to more than three days, and enhance binding affinity to the obligate co-receptor, beta-klotho.[29,31] Efruxifermin has balanced *in vitro* potency across FGF21's receptors, FGFR1c, FGFR2c, and FGFR3c[31] and high systemic exposure *in vivo*,[29] enabling it to act on adipose tissue and the liver, the major sources of liver fat. It is inactive at FGFR4, thereby avoiding an undesirable increase in LDL-cholesterol (LDL-C) levels. In a phase IIa study of patients with F1–F3 NASH, efruxifermin significantly reduced liver fat and markers of liver injury, decreased fibrosis, improved glucose and lipid metabolism, and reduced hyperuricemia, with a trend to weight loss.[32] Notable among these improvements was a 2-stage reversal of fibrosis in 11 of 22 (50%) patients with F2 or F3 NASH after only 16 weeks of treatment.[32]

This study reports results from Cohort C, an expansion cohort of the phase IIa study, in patients with compensated cirrhosis (F4) due to NASH. The primary objective was to assess safety and tolerability of efruxifermin in these patients, who are at the highest risk of progressing to hepatic decompensation and liver failure, and therefore have the greatest unmet medical need. Secondary objectives were to establish proof-of-concept for efficacy of efruxifermin in patients with compensated cirrhosis by evaluating its effects on liver stiffness and biomarkers of liver fibrosis. Exploratory objectives included evaluation of effects on markers of liver injury, function, and histology, as well as whole-body metabolism.

Patients and methods

Study design and participants

Cohort C was a randomized, double-blind, placebo-controlled phase IIa expansion cohort of the BALANCED study (NCT03976401) that evaluated safety and tolerability of efruxifermin in patients with NASH and compensated cirrhosis (F4 fibrosis). This study was conducted at 18 gastroenterology or hepatology clinics in the USA.

Eligible patients were 18 to 80 years of age with biopsy-confirmed compensated cirrhosis due to NASH, as documented by a local pathologist. Participants were required to have a FibroScan measurement >13.1 kPa and enhanced liver fibrosis (ELF) score >9.8, and no evidence of worsening levels of serum alanine aminotransferase (ALT) and aspartate aminotransferase (AST) between screening and randomization. Patients must have had a glomerular filtration rate ≥60 ml/min; hemoglobin A1c (HbA1c) ≤9.5%; hemoglobin ≥11 g/dl; international normalized ratio (INR) ≤1.3; direct bilirubin ≤0.3 mg/dl; total bilirubin ≤1.3 × upper limit of normal (ULN); creatine kinase <3 × ULN; platelet count ≥125,000/μl; serum triglyceride level ≤500 mg/dl; ALT and AST <5 × ULN; alkaline phosphatase (ALP) <2 × ULN; and albumin ≥3.5 g/dl. Patients with decompensated liver disease, liver transplantation, hepatocellular carcinoma or other chronic liver diseases including viral hepatitis were excluded. Patients were allowed to continue with their anti-diabetic medications including glucagon like peptide-1 receptor agonists or sodium-glucose transport protein 2 inhibitors, provided they were on a stable dose for at least 6 months prior to screening. All participants provided written informed consent before enrollment. All inclusion and exclusion criteria are provided in the supplementary information (Table S1).

Eligible participants were randomly assigned in a 1:2 ratio to placebo or efruxifermin 50 mg treatment groups, for a total study period of 26 weeks: 6 weeks for screening, 16 weeks on-treatment, 4 weeks of safety follow-up (Fig. S1). A qualified staff member administered the study drug (1.0 ml s.c. injection) into the abdomen of each participant at weekly intervals. The 50 mg dose of efruxifermin was chosen based on results from the BALANCED main study,[32] as optimally balancing efficacy with safety and tolerability.

Interactive response technology (IRT) was used for centralized randomization and treatment assignment. Study site personnel obtained the treatment assignment from the IRT.

Study drug (efruxifermin or placebo) was prepared by an independent, unblinded, qualified healthcare professional (such as a pharmacist not associated with the study). Efruxifermin and placebo were identical in appearance and administered in a blinded fashion to the participants. All investigators, staff, sponsor, and patients were blinded to treatment assignment.

Study assessments

Safety and tolerability

Safety and tolerability of efruxifermin were assessed through the reporting of adverse events (AEs), treatment-emergent adverse

events (TEAEs), clinical laboratory tests, electrocardiograms, vital sign assessments, body weight, anti-drug antibodies (ADA) including neutralizing activity (NAb), and concomitant medication usage. Markers of liver function (albumin, bilirubin, model for end-stage liver disease [MELD] score, Child-Pugh score [C–P]); hemostasis (INR, fibrinogen, platelets, and plasminogen activator inhibitor-1 [PAI-1]), a marker of systemic inflammation (high-sensitivity C-reactive protein [hs-CRP]), biomarkers of bone turnover (osteocalcin, procollagen type-I N-terminal propeptide [P1NP], C-terminal telopeptide of type 1 collagen [CTX-1], bone specific alkaline phosphatase, parathyroid hormone, and vitamin D), and a marker of hypothalamus-pituitary-adrenal (HPA) axis activation (salivary cortisol) were obtained at baseline and every 4 weeks throughout the study. Dual-energy X-ray absorptiometry (DXA) scan measurements, including bone mineral density of the lumbar spine, femoral neck, total hip, and percent body fat, were performed at baseline and week 16. The safety follow-up visit was performed at week 20, 4 weeks after the last dose of study drug.

Clinical laboratory tests were performed by Medpace Reference Laboratories (Cincinnati, Ohio). ADA and NAb assays were performed by Precision for Medicine Bioanalytical Laboratory (Redwood City, California). Immunogenicity data analysis and reporting were conducted by B2S Life Sciences (Franklin, Indiana).

Non-invasive markers of fibrosis and liver injury

Efficacy was evaluated as change from baseline to week 16 in non-invasive markers of fibrosis, including liver stiffness by transient elastography (FibroScan) and serum markers N-terminal type III collagen propeptide (Pro-C3), ELF score and its components (hyaluronic acid; procollagen type III N-terminal propeptide [P3NP]; and tissue inhibitor of metalloproteinase-1 [TIMP-1]). ALT, AST, bilirubin, gamma-glutamyltransferase (GGT), ALP, and urate were measured every 4 weeks as biomarkers of liver injury.

In response to requests from participants and investigators, the protocol was amended to assess the impact of treatment on NASH histopathology by liver biopsy obtained on a voluntary basis at week 16. Baseline and end-of treatment biopsies were read by a single pathologist (central reader, Pacific Rim Pathology, San Diego, California). For all biopsies, the reader was blinded to treatment, sequence, and participant. Imaging aspects of the study were conducted by Perspectum Diagnostics (Oxford, United Kingdom).

Markers of metabolism

Markers of lipid metabolism (triglycerides, LDL-C, HDL-cholesterol [HDL-C], non-HDL-cholesterol [non-HDL-C]), insulin sensitivity (C-peptide, insulin, adiponectin), glycemic control (HbA1c), and body weight were measured at baseline and at weeks 4, 8 12, 16, and 20.

Outcome measures

The primary endpoint was safety and tolerability of efruxifermin in patients with compensated cirrhosis due to NASH. Secondary endpoints included change from baseline to week 16 in liver stiffness and non-invasive biomarkers of fibrosis: ELF score and serum levels of Pro-C3. Exploratory endpoints included changes in liver histopathology, as well as in markers of liver injury and of glucose and lipid metabolism. A full list of exploratory endpoints is provided in the supplementary information (Table S2).

Statistical analyses

Since the primary endpoint was safety and tolerability, sample size was determined based on clinical considerations. Cross-treatment comparison of secondary and exploratory endpoints was not powered to detect pre-specified differences. Statistical analyses were conducted by Medpace (Cincinnati, Ohio).

Demographic and baseline characteristics were summarized with descriptive statistics. For the primary endpoint, safety and tolerability of efruxifermin, the safety set included all individuals who received at least one dose of study drug. All data collected during treatment through 30 days after the last dose of study drug were included in the safety analyses.

For the secondary endpoint of liver stiffness, analysis of covariance (ANCOVA) was used to determine the absolute and percent change from baseline to week 16, with treatment group as a factor and baseline liver stiffness as a covariate using the full analysis set. Normality of the residuals was tested by the Shapiro-Wilk test.

For the secondary endpoints of non-invasive biomarkers of fibrosis (ELF test score and Pro-C3) and exploratory endpoints including markers of liver injury (AST, ALT, GGT, ALP), liver function (albumin, bilirubin, MELD score, C–P score), hemostasis (platelet count, INR,), HPA (salivary cortisol) and body weight, an ANCOVA model with treatment group as a factor and baseline value as a covariate was used. Exploratory biomarkers of lipid metabolism, insulin sensitivity (C-peptide, insulin, adiponectin), glycemic control (HbA1c), and DXA scan measurements were also analyzed as absolute and percent change from baseline by treatment using an ANCOVA model with treatment group as a factor and baseline value as a covariate. Analysis of urate, fibrinogen, PAI-1 and hs-CRP was performed using mixed-model repeated measures with absolute change from baseline as the dependent variable and treatment group and visit as factors and baseline value as a covariate, as well as interaction of treatment group by visit. For all of the above, least squares (LS) mean, 95% CIs, and *p* values for change from baseline as well as difference from placebo were calculated. The Shapiro-Wilk normality test was performed for residuals from the model; if the *p* value was <0.01, non-parametric analyses were conducted. Non-parametric analyses are presented for the ELF score and for its individual components (P1NP, hyaluronic acid, TIMP-1), where *p* values are from the Wilcoxon rank-sum test.

The liver biopsy analysis set included all patients with week 16 liver biopsies whose stage-4 fibrosis at baseline was confirmed by the central reader. Data were analyzed according to the proportion of individuals at week 16 whose fibrosis regressed by ≥1 stage; fibrosis regressed by ≥1 stage with no worsening in non-alcoholic fatty liver disease activity score (NAS); NAS improved by ≥2 points; NAS improved by ≥1-point in any of steatosis, lobular inflammation, or hepatocellular ballooning; or NASH resolved (*i.e.*, 0- or 1-point inflammation, 0-point ballooning).

Study oversight

The study protocol and all amendments were approved by an institutional review board and independent ethics committee for each site in compliance with the ethical principles of the Declaration of Helsinki, and consistent with the International Conference on Harmonization of Good Clinical Practice and applicable regulatory guidelines. The study was designed and conducted according to the Sponsor's (Akero Therapeutics, Inc) protocol. An independent data monitoring committee reviewed

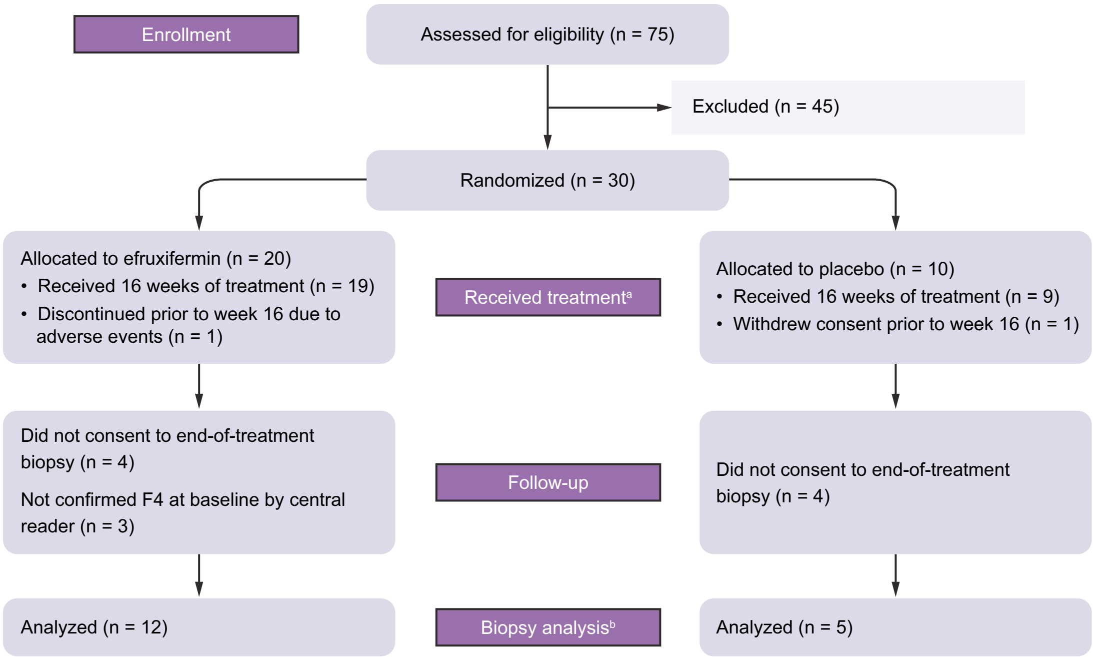

Fig. 1. CONSORT clinical study flow diagram. [a]Comprises the full analysis set and [b]comprises the biopsy analysis set.

progress and provided oversight. Further information regarding the methods is available in the CONSORT statement.

Results

Patient disposition

Between 4 June 2020 and 23 September 2020, 75 patients were screened at 18 sites in the USA. A total of 30 patients with compensated cirrhosis due to NASH were enrolled and randomized to receive either efruxifermin (n = 20) or placebo (n = 10). Follow-up continued until 10 February 2021. All patients started the study, with two patients discontinuing prior to week 16: one placebo patient withdrew consent, and one efruxifermin patient withdrew due to adverse events. The remaining patients (19 efruxifermin and 9 placebo) completed 16 weeks of treatment. Eight patients (4 in each group) did not consent to optional end-of-treatment biopsy. Three efruxifermin patients assessed originally by a local pathologist as F4 were not confirmed as F4 by the central reader, thus were not included in the biopsy analysis set. Of these three patients, two were considered difficult to stage by the central pathologist and one patient was identified as a borderline F3. The remaining 17 patients with end-of-treatment biopsies were confirmed as F4 patients by the central reader and comprised the biopsy analysis set: 12 efruxifermin-treated and 5 in the placebo group (Fig. 1). Non-invasive markers of fibrosis were evaluated in all individuals (full analysis set) and in the liver biopsy analysis set.

Baseline demographics and disease characteristics

The demographics and baseline characteristics of participants in this study are representative of patients with compensated cirrhosis due to NASH and were generally balanced across treatment groups with a few exceptions (Table 1), potentially due to the small sample size. The mean age was 61 and 57 years in efruxifermin and placebo groups, respectively, while baseline body weight was higher in the placebo group. The efruxifermin-treated group was predominantly female (n = 16; 80%), while the placebo group was mostly male (n = 7; 70%). Pathophysiological characteristics reflect the advanced NASH stage of F4 patients, as indicated by mean AST and ALT values in the low thirties, ELF scores ranging between 9.7 and 10.4, and relatively high values for Pro-C3 (22.6 to 25.6 μg/L) and liver stiffness (22.1 to 25.8 kPa).

Mean triglyceride levels are also lower than earlier stages of NASH, likely due to lower capacity for secretion of VLDL resulting from fewer healthy hepatocytes in patients with cirrhosis. Half of the patients had type 2 diabetes, with mean baseline HbA1c values of 6.5–7.3%, compared to 5.7% in patients without diabetes.

In the full analysis set, based on levels of fibrosis biomarkers Pro-C3 and ELF, the efruxifermin-treated group appears to be slightly more advanced compared to the placebo group. However, in the liver biopsy analysis set (n = 17), baseline levels of these fibrosis biomarkers were comparable across both treatment groups as shown in Table S3. The small number of patients in each group likely contributed to the apparent variability across some of the baseline characteristics.

Safety and tolerability

The primary objective was to determine safety and tolerability of efruxifermin in patients with compensated cirrhosis due to NASH. Overall, efruxifermin appeared to be well-tolerated, with a similar safety profile to that observed in patients with fibrosis stage 1-3.[32] No new population-specific safety concerns were noted. Twenty-seven (90%) participants reported one TEAE: 19 (95.0%) in the efruxifermin group and 8 (80.0%) in the placebo group. The majority of TEAEs were grade 1 (n = 7, 23.3%) or grade 2 (n = 19, 63.3%). Table 2 lists all TEAEs reported in ≥15% of patients in either treatment group. Compared to placebo, more patients in the efruxifermin group experienced TEAEs of special interest, which included diarrhea, injection site reactions

JHEP|Reports

Table 1. Baseline demographics and disease characteristics.

Parameter	Placebo (n = 10)	Efruxifermin (n = 20)
Mean age, years (SD)	57.1 (14.4)	61.1 (10.0)
Sex, n (%)		
Male	7 (70.0)	4 (20.0)
Female	3 (30.0)	16 (80.0)
Race or ethnicity, n (%)		
White	10 (100.0)	18 (90.0)
Black or African American	0	1 (5.0)
Native Hawaiian or other Pacific Islander	0	1 (5.0)
Hispanic or Latino	5 (50.0)	8 (40.0)
Metabolic risk factors and parameters, mean (SD)		
Body weight, kg	119.1 (30.5)	97.9 (19.8)
Body mass index, kg/m^2	39.1 (8.2)	36.0 (5.6)
Type 2 diabetes, n (%)	5 (50.0)	10 (50.0)
Markers of liver health, mean (SD)		
Alanine aminotransferase, U/L	32.7 (20.0)	31.7 (16.8)
Aspartate aminotransferase, U/L	28.9 (21.1)	31.4 (13.7)
Gamma-glutamyltransferase, U/L	46.7 (17.7)	75.8 (39.2)
Alkaline phosphatase, U/L	67.2 (19.0)	77.9 (24.6)
Urate, mg/dl	5.9 (1.4)	6.0 (1.2)
High-sensitivity C-reactive protein, mg/L	4.9 (5.8)	6.7 (8.4)
Liver steatosis, CAP score, dB/m	336.9 (48.5)	299.2 (59.3)
Markers of fibrosis, mean (SD)		
Pro-C3, ug/L	22.6 (11.8)	25.6 (27.5)
ELF score	9.7 (0.8)	10.4 (1.2)
Liver stiffness, kPA	25.8 (13.2)	22.1 (10.8)
Markers of liver function, mean (SD)		
Bilirubin, mg/dl	0.8 (0.2)	0.7 (0.3)
Albumin, g/dl	4.3 (0.2)	4.2 (0.3)
Platelets, 10-9/L	166.8 (29.5)	191.7 (34.5)
Fibrinogen, mg/dl	407.0 (93.7)	452.2 (64.6)
International normalized ratio	1.1 (0.1)	1.1 (0.1)
Plasminogen activator inhibitor-1, IU/ml	14.0 (11.8)	14.5 (10.6)
Model for end-stage liver disease score	6.9 (0.9)	7.4 (1.0)
Child-Pugh score	5.0 (0.0)	5.1 (0.2)
NAFLD activity score, mean (SD)	3.3 (2.1)	4.1 (1.7)
Markers of lipid metabolism, mean (SD)		
Total cholesterol, mg/dl	157.1 (42.9)	167.4 (40.4)
Triglycerides, mg/dl	121.7 (59.6)	134.6 (62.8)
LDL-C, mg/dl	89.5 (34.7)	90.1 (34.4)
HDL-C, mg/dl	43.3 (12.4)	50.4 (13.8)
Non-HDL-C, mg/dl	113.8 (43.3)	117.1 (41.2)
Apolipoprotein B, mg/dl	82.8 (26.1)	82.6 (26.5)
Apolipoprotein C-III, mg/dl	6.3 (3.3)	7.8 (4.0)
Lipoprotein-a (nmol/L)	48.7 (71.9)	61.9 (66.7)
Beta -hydroxybutyrate (mmol/L)	0.1 (0.1)	0.1 (0.1)
Bile acids (µmol/L)	10.3 (6.7)	9.5 (8.8)
Markers of glycemic control, mean (SD)		
Fasting serum glucose, mg/dl	122.9 (26.4)	107.3 (16.4)
HbA1c, %	6.6 (1.4)	6.1 (1.0)
Patients with type 2 diabetes	7.3 (1.6), n = 5	6.5 (1.1), n = 10
Patients without type 2 diabetes	5.8 (0.7), n = 4	5.7 (0.5), n = 9
C-peptide, ug/L	5.7 (2.1)	5.1 (1.0)
Insulin, mIU/L	38.7 (21.9)	29.4 (9.3)
HOMA-IR	12.5 (8.1)	8.4 (3.7)
Adiponectin, mg/L	4.8 (2.5)	5.8 (2.9)
Concomitant medications of interest, n (%)		
Statins	3 (30.0)	6 (30.0)
GLP-1 receptor agonists	3 (30.0)	3 (15.0)

CAP, controlled attenuation parameter; HOMA-IR, homeostatic model assessment of insulin resistance.

(including bruising, erythema, pruritus, and rash), and decreased blood glucose. The most frequent AEs were gastrointestinal, predominantly mild or moderate, occurring primarily during the first 4 weeks of treatment, generally transient and resolving without treatment. Injection site reactions were infrequent, transient, and of mild severity (all grade 1). There were no serious AEs in the efruxifermin group. One placebo-treated patient (3.3%) experienced a grade 4 TEAE of pulmonary embolism, considered unrelated to study treatment or procedures. One patient treated with efruxifermin discontinued at week 5 due to adverse events, experiencing abdominal distension (grade 1), constipation (grade 1), diarrhea (grade 1) and pruritus (grade 1). There were no deaths and no cases of hepatic decompensation or drug-induced liver injury. Most hemostasis parameters

Table 2. Safety and tolerability.

Safety overview	Placebo (n = 10)	Efruxifermin (n = 20)
Study discontinuations	1[a]	1[b]
Deaths	0	0
Any treatment-emergent adverse events, n (%)	8 (80)	19 (95)
Treatment-emergent adverse events, n (%)		
Life-threatening	1 (10)	0
Severe	0	0
Moderate	5 (50)	14 (70)
Mild	2 (20)	5 (25)
Study procedure related	1 (10)	7 (35)
Treatment-emergent events leading to discontinuation, n (%)	0	1 (5)
Drug-related treatment-emergent adverse events, n (%)	3 (30)	13 (65)
Serious adverse events	1[c]	0
**Treatment-emergent events occurring in ≥15% of patients in any group, n (%)**		
Gastrointestinal disorders	6 (60)	14 (70)
Diarrhea	1 (10)	10 (50)
Nausea	2 (10)	9 (45)
Vomiting	0	4 (20)
Abdominal pain	2 (20)	3 (15)
Constipation	0	4 (20)
Gastroesophageal reflux disease	2 (20)	0
Nervous system disorders	4 (40)	8 (40)
Headache	1 (10)	4 (20)
General disorders and administration site conditions	1 (10)	10 (50)
Injection site reaction	0	6 (30)
Injection site bruising	1 (10)	4 (20)
Injection site erythema	0	5 (25)
Infections and infestations	2 (20)	7 (35)
Sinusitis	0	4 (20)
Skin and subcutaneous tissue disorders	0	9 (45)
Pruritus	0	3 (15)

[a] Withdrawal of consent.
[b] Abdominal distention, constipation, diarrhea, pruritus.
[c] Pulmonary embolism.

either remained stable (INR) or trended to improve (platelets, PAI-1) (Table S5, Fig. 2). There were no increases in MELD or C–P score, and markers of liver function (*e.g.*, total/direct bilirubin, albumin) were unchanged. A marker of systemic inflammation, hs-CRP, was significantly reduced from baseline at week 8 and trended lower after 12 and 16 weeks of efruxifermin treatment (Fig. 2).

Vital signs including heart rate and blood pressure were unchanged. Bone mineral density was unchanged over 16 weeks of treatment. Consistent with this, levels of CTX-1 (a bone resorption marker) were unchanged relative to baseline (p = 0.2445) and to placebo (p = 0.7114). However, levels of P1NP (a biomarker of type-I collagen synthesis in EFX-treated group any tissue or organ in the body) were approximately 20% lower (p = 0.0211 *vs.* placebo). Levels of salivary cortisol (a marker of HPA axis activation) were unchanged throughout the treatment period.

Of the patients who received efruxifermin, 11 of 20 (55%) tested positive for treatment-emergent ADAs by week 20. The observed titers were low (1:26.01 or lower) and were first detected at week 8. Sensitivity of the ADA assay, at 5.574 ng/ml, is >15-fold more sensitive than the FDA-recommended 100 ng/ml.[33] No patients developed NAbs to efruxifermin. One ADA-positive patient had high triglyceride levels which could have interfered with detection of NAbs. One efruxifermin-treated patient (8.3% of ADA-positive patients) had antibodies which cross-reacted with endogenous FGF21. Utilizing individual patient-level elevation of serum adiponectin or reduction in serum triglycerides as pharmacodynamic markers, there was no discernible attenuation of response to efruxifermin associated with development of anti-efruxifermin antibodies.

Non-invasive markers of fibrosis

Liver stiffness was significantly decreased over 16 weeks of efruxifermin treatment (LS mean absolute change from baseline [CFB] −5.7 kPa, p = 0.0036 *vs.* baseline [LS mean relative CFB −24.4%, p = 0.0007 *vs.* baseline]) compared to a smaller decrease for placebo (LS mean absolute CFB −1.9 kPa, p = 0.4519 *vs.* baseline; LS mean relative CFB of −7.6% [p = 0.3970 *vs.* baseline]) (Fig. 3A). The reduction in liver stiffness in efruxifermin-treated patients over 16 weeks was not statistically significantly different from placebo (p = 0.1326), and was comparable in patients with or without type 2 diabetes.

Serum markers of fibrosis and fibrogenesis, ELF score and Pro-C3, improved significantly following 16 weeks of treatment with efruxifermin. ELF score decreased by 0.4 (median absolute CFB, p = 0.0036 *vs.* placebo) over 16 weeks of treatment with efruxifermin, while placebo was associated with a median absolute CFB of +0.4 (Fig. 3A). Components of the ELF score (Table 3) also appeared to improve, with significant reductions in P3NP in the treated group (−3.2 μg/L; p = 0.0019 *vs.* placebo) compared to placebo (+1.8 μg/L). Significant reductions from baseline were also noted in hyaluronic acid (CFB −19.4 μg/L in the efruxifermin-treated group compared to +16.4 μg/L in the placebo group; p = 0.0139) and in TIMP-1 (CFB -35.7 μg/L in the treated group compared to +17.6 μg/L in placebo; p = 0.0415).

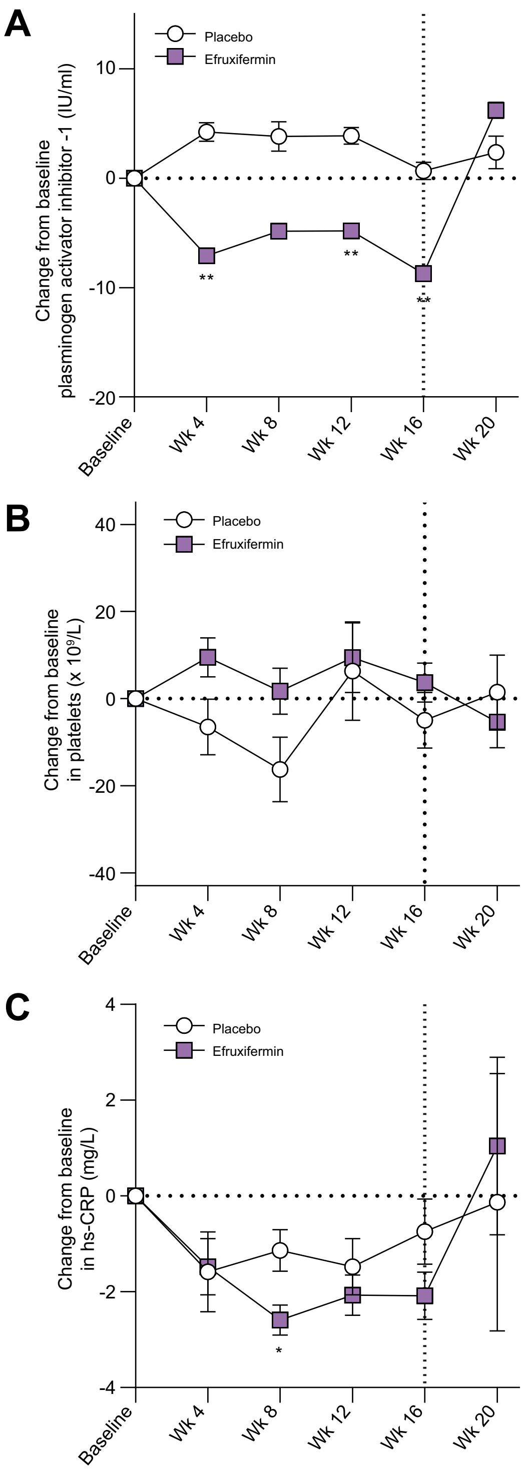

Fig. 2. Time courses for absolute change from baseline in levels of plasminogen activator inhibitor-1, platelets, and high-sensitivity C-reactive protein. Significance is indicated by asterisks: *p <0.05, **p <0.01 *vs.* placebo (MMRM). The vertical line at week 16 indicates the end of the treatment period, with safety follow-up continuing to week 20.

Sixteen weeks of treatment with efruxifermin significantly reduced the Fibroscan-AST or FAST score, a composite biomarker for non-invasively identifying patients at risk of progressive NASH, combining liver stiffness measurement, controlled attenuation parameter (CAP), and AST (Fig. 3B).[34]

Pro-C3 was also significantly decreased over 16 weeks (LS mean absolute CFB −9.0 μg/L, p = 0.0130 *vs.* placebo [LS mean relative CFB -16.2%]) compared to a smaller decrease on placebo (LS mean absolute CFB −3.4 μg/L [LS mean relative CFB +3.9%]) (Fig. 3C, Table 3). Although Pro-C3 declined over time in the placebo group, potentially as a result of implementation of lifestyle intervention and regular clinical evaluation per standard of care, the magnitude of reduction was significantly less than in efruxifermin-treated patients.

Non-invasive markers of fibrosis were also evaluated in the liver biopsy analysis set comprising those individuals with available end-of-treatment biopsies and confirmed cirrhosis at baseline; the results were comparable to those observed in the full analysis set (Table S4).

Exploratory endpoints

Markers of liver injury

ALT levels were significantly decreased over 16 weeks of efruxifermin treatment (LS mean absolute CFB −10.3 U/L [LS mean relative CFB −22.1%]) by comparison with placebo (LS mean absolute CFB −1.3 U/L [LS mean relative CFB +3.0%], p = 0.0098, efruxifermin *vs.* placebo) (Fig. 3D, Table 3). Four weeks after the last dose, ALT had returned to near-baseline levels. AST showed a similar numerical trend to ALT for LS mean absolute CFB (SE) in treated patients of −9.6 (1.6) U/L compared to an LS mean absolute CFB of −4.5 (2.2) U/L for placebo (p = 0.0752, efruxifermin *vs.* placebo). The relative reduction in AST at week 16 (LS mean CFB of −25.8%) for the treated group was significantly (p = 0.0112) greater than −5.5% for placebo (Fig. 3E, Table 3). Of the other markers of liver injury, GGT (Fig. 3F, Table 3) and ALP (Fig. 3G, Table 3) showed numerical but not statistically significant reductions in treated patients as early as week 8 that appeared to be sustained through week 16, compared to increases in the placebo group. For GGT, the LS mean (SE) absolute CFB was -14.5 (13.7) U/L for treated patients, compared to +21.3 (19.3) U/L for placebo (p = 0.1569, efruxifermin *vs.* placebo). For ALP, the LS mean (SE) absolute CFB to week 16 was -2.9 (4.8) U/L for treated patients, compared to +4.6 (6.7) for placebo (p = 0.3748, efruxifermin *vs.* placebo). Levels of urate, another marker of hepatocyte stress, decreased significantly more in treated patients (LS mean absolute CFB of −0.98 mg/dl; p = 0.0357 *vs.* placebo [relative CFB -16.19%]; p = 0.0143 *vs.* placebo), compared to an LS mean absolute CFB of -0.34 mg/dl (relative CFB −4.56%) for the placebo group (Fig. 3H, Table 3).

Histology of liver biopsies.

The apparent reduction in non-invasive markers of fibrosis with efruxifermin treatment was associated with evidence of fibrosis regression in this small cohort of patients with paired biopsies. Four (33%) of 12 efruxifermin-treated patients with confirmed cirrhosis at baseline and available end-of-treatment biopsies achieved fibrosis improvement of ≥1 stage without worsening of NASH. By comparison, in the placebo group, none of the 5 patients with available end-of-treatment biopsies achieved this threshold (Fig. 4A). Three (25%) efruxifermin-treated patients achieved NASH resolution (0 to 1-point inflammation, 0-point ballooning) compared to none in the placebo group (Fig. 4B). Patients whose fibrosis improved without NAS worsening were distinct from those who had NASH resolution. Seven of 12 patients (58%) in the efruxifermin

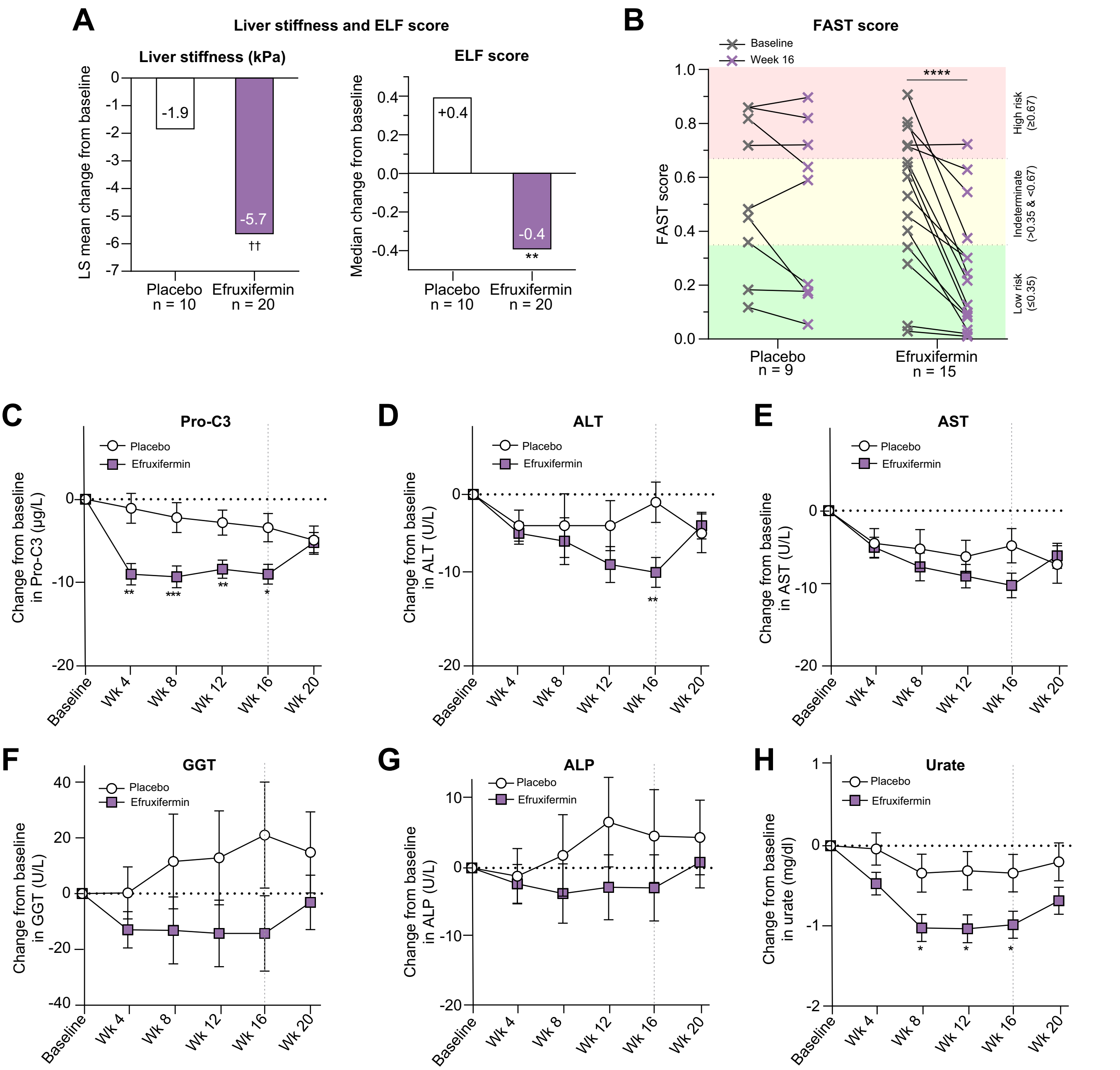

Fig. 3. Absolute change from baseline to week 16 for liver stiffness and ELF score, pre- and post-treatment FAST score, time course for Pro-C3 and liver enzymes, and absolute change in urate from baseline. Values are shown as LS mean CFB values ± SEs, with *p* values from an ANCOVA model for FibroScan liver stiffness, Pro-C3, ALT, AST, GGT, ALP; Wilcoxon rank-sum test for median change in ELF score; and an MMRM for urate. *p* values for FAST score are from repeated-measures two-way ANOVA followed by Sidak's multiple comparisons test. Refer to statistical analysis in methods for details. For ALT, AST, GGT, and ALP, the last observation carried forward method was used for patients with missing values. For urate, only individuals from the full analysis set with non-missing baseline values and the specified visit are included. Significance is indicated by asterisks: $^{*}p<0.05$, $^{**}p<0.01$, $^{***}p<0.001$, $^{****}p<0.0001$ *vs.* placebo; $^{\dagger\dagger}p<0.01$ *vs.* baseline. The vertical line at 16 weeks indicates end-of-treatment period. ALP, alkaline phosphatase; ALT, alanine aminotransferase; ANCOVA, analysis of covariance; AST, aspartate aminotransferase; CFB, change from baseline; ELF, enhanced liver fibrosis; FAST, FibroScan-AST; GGT, gamma-glutamyltransferase; LS, least-square; MMRM, mixed-model repeated measures; Pro-C3, N-terminal type III collagen propeptide.

group at week 16 had a ≥2-point improvement in NAS compared with 1 of 5 patients (20%) in the placebo group. Overall, 9/12 efruxifermin patients (75%) with confirmed cirrhosis at baseline and end-of-treatment biopsies experienced either fibrosis improvement without worsening of NASH, NASH resolution, or improvement in NAS by ≥2-points, compared to 1 of 5 (20%) placebo patients.

Markers of lipid and glucose metabolism

Markers of lipid (Fig. 5A-D) and glucose metabolism (Fig. 5E-G) are consistent with efruxifermin restoring a healthier whole-body metabolic phenotype. Lipoprotein profiles were significantly improved after 16 weeks treatment, with reductions of 29% for triglycerides ($p<0.0001$ *vs.* baseline; $p = 0.0020$ *vs.* placebo), 14% for non-HDL-C ($p = 0.0004$ *vs.* baseline; $p =$

Table 3. Change from baseline to week 16 in non-invasive markers of fibrosis, ELF score and its components, and markers of liver injury.

	Placebo (n = 10)		Efruxifermin (n = 20)		
	Value	*p vs.* baseline	Value	*p vs.* baseline	*p vs.* placebo
Liver stiffness (kPa)					
Baseline mean (SD)	25.8 (13.2)		22.6 (10.9)		
LS mean absolute CFB (95% CI)[1]	−1.9 (−7.0, 3.2)	0.4519	−5.7 (−9.4, −2.0)	0.0036	0.2226
LS mean percent CFB (95% CI)[1]	−7.6 (−25.6, 10.5)	0.3970	-24.4 (−37.5, −11.4)	0.0007	0.1326
Pro-C3 (μg/L)					
Baseline mean (SD)	22.6 (11.8)		28.9 (30.8)		
LS mean absolute CFB (95% CI)[1]	−3.4 (−6.9, 0.2)	0.0620	−9.0 (−11.5, −6.5)	<0.0001	0.0130
LS mean percent CFB (95% CI)[1]	3.9 (−17.7, 25.4)	0.7168	−16.2 (−31.4, −1.0)	0.0379	0.1309
ELF score					
Baseline, median	9.5		10.4		
Median absolute CFB (IQR)[2]	0.4 (0.0, 0.6)	NC	−0.4 (−0.9, −0.0)	NC	
Median difference *vs.* placebo[2]			−0.8 (−1.3, −0.4)		0.0036
Hyaluronic acid, μg/L					
Baseline, median	63.8		116.9		
Median absolute CFB (IQR)[2]	16.4 (1.8, 69.9)	NC	−19.4 (−58.3, 6.4)	NC	
Median difference *vs.* placebo[2]			−59.3 (−86.6, −11.1)		0.0139
P3NP, μg/L					
Baseline, median	8.2		11.6		
Median absolute CFB (IQR)[2]	1.8 (0.3, 3.4)	NC	−3.2 (−5.7, −1.2)	NC	
Median difference *vs.* placebo[2]			−5.9 (−9.0, −2.7)		0.0019
TIMP-1, μg/L					
Baseline, median	230.2		279.5		
Median absolute CFB (IQR)[2]	17.6 (−14.3, 43.0)	NC	−35.7 (−74.1, 3.6)	NC	
Median difference *vs.* placebo[2]			−52.3 (−112.9, −6.9)		0.0415
ALT, U/L					
Baseline mean (SD)	32.7 (20.0)		31.8 (17.2)		
LS mean absolute CFB (95% CI)[1]	−1.3 (−6.7, 4.1)	0.6289	−10.3 (−14.3, −6.4)	<0.0001	0.0098
LS mean percent CFB (95% CI)[1]	3.0 (−14.5, 20.4)	0.7301	−22.1 (−34.8, −9.5)	0.0014	0.0244
AST, U/L					
Baseline mean (SD)	28.9 (21.1)		31.6 (14.0)		
LS mean absolute CFB (95% CI)[1]	−4.5 (−9.0, 0.1)	0.0566	−9.6 (−12.9, −6.2)	<.0001	0.0752
LS mean percent CFB (95% CI)[1]	−5.5 (−17.9, 6.8)	0.3654	−25.8 (−34.8, −16.9)	<.0001	0.0112
GGT, U/L					
Baseline mean (SD)	46.7 (17.7)		76.2 (40.2)		
LS mean absolute CFB (95% CI)[1]	21.3 (−18.4, 61.0)	0.2808	−14.5 (−42.6, 13.6)	0.2996	0.1569
LS mean percent CFB (95% CI)[1]	42.2 (−16.1, 100.5)	0.1487	−23.0 (−64.2, 18.3)	0.2624	0.0817
ALP, U/L					
Baseline mean (SD)	67.2 (19.0)		77.9 (24.6)		
LS mean absolute CFB (95% CI)[1]	4.6 (−9.2, 18.4)	0.4988	−2.9 (−12.9, 7.0)	0.5468	0.3748
LS mean percent CFB (95% CI)[1]	6.5 (−10.6, 23.7)	0.4412	−2.4 (−14.7, 10.0)	0.6956	0.3999
Urate, mg/dl					
Baseline mean (SD)	5.87 (1.41)		6.04 (1.22)		
LS mean absolute CFB (95% CI)[3]	−0.34 (−0.82, 0.14)	0.1526	-0.98 (−1.32, −0.63)	<.0001	0.0357
LS mean percent CFB (95% CI)[3]	−4.56 (−11.95, 2.83)	0.2164	−16.19 (−21.52, −10.86)	<.0001	0.0143

ALP, alkaline phosphatase; ALT, alanine aminotransferase; AST, aspartate aminotransferase; CFB, change from baseline; ELF, enhanced liver fibrosis; GGT, gamma-glutamyltransferase; NC, not calculated; LS, least squares; Pro-C3, N-terminal type III collagen propeptide; P3NP, procollagen type III N-terminal propeptide; TIMP-1, tissue inhibitor of metalloproteinase-1.
[1] *p* values from ANCOVA.
[2] *p* values from Wilcoxon rank-sum test.
[3] *p* values from mixed-effects model of repeated measure.

0.0002 *vs.* placebo), and 8% for LDL-C (p =0.0961 *vs.* baseline; *p* = 0.0056 *vs.* placebo), compared to placebo patients, who experienced increases of 0.8% for triglycerides (*p* = 0.9289 *vs.* baseline), 11.8% for non-HDL-C (*p* = 0.0211 *vs.* baseline), and 16% for LDL-C (*p* = 0.0198 *vs.* baseline). Consistent with restoration of a healthier lipoprotein profile, HDL-C increased by 33% (p <0.0001 *vs.* baseline; *p* = 0.0062 *vs.* placebo) in efruxifermin-treated patients compared to 3% for placebo (*p* = 0.7581 *vs.* baseline). Among other evaluated markers of lipid metabolism, efruxifermin treatment appeared to significantly (*p* = 0.0004 *vs.* placebo) reduce apolipoprotein B levels (LS mean CFB: -11.1%) compared to an increase of 7.1% for placebo, but apolipoprotein C-III was unchanged (LS mean CFB +15% for the treated group compared to +20% for placebo; *p* = 0.7220), and lipoprotein-a levels appeared to increase significantly (LS mean CFB +32.7% for treated group compared to +8.4% for placebo, *p* = 0.0203). Efruxifermin treatment was associated with a numerically higher serum concentration of beta-hydroxybutyrate (LS mean CFB +68.4% for the treated group compared to +37.8% for placebo, *p* = 0.4448). Levels of bile acids in the treated group did not appear to be different from placebo (LS mean CFB +39% for the treated group compared to +54% for placebo, *p* = 0.7083).

After 16 weeks of treatment, efruxifermin elicited statistically significant and potentially clinically meaningful improvements in multiple serum markers of glucose metabolism. Mean

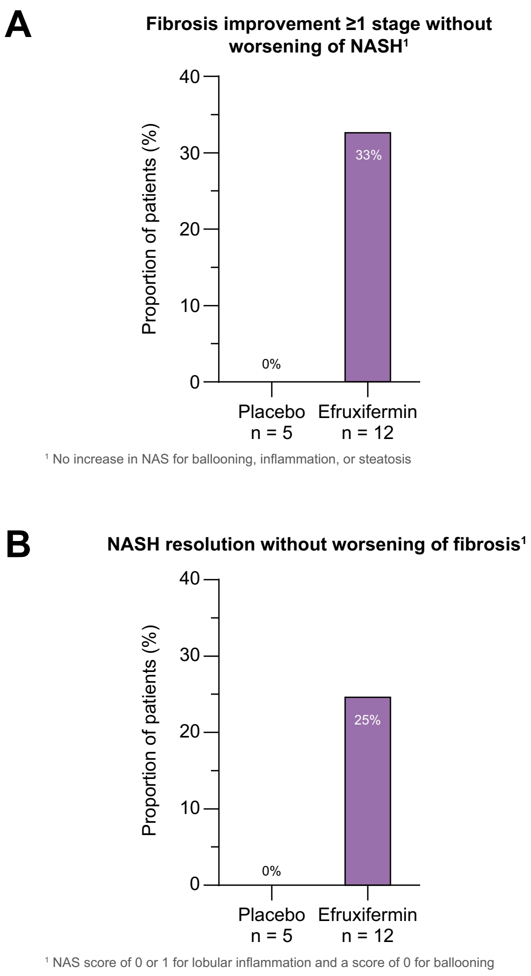

Fig. 4. Proportion of patients with NASH cirrhosis achieving an improvement in fibrosis stage or NASH resolution after 16 weeks. The study was not powered to assess statistical significance of histological endpoints. NAS, non-alcoholic fatty liver disease activity score; NASH, non-alcoholic steatohepatitis.

HbA1c declined by 0.4% (absolute CFB, p = 0.0011 *vs.* placebo) across all treated patients, compared with an increase of 0.1% for placebo. For the subset of patients with type 2 diabetes, all of whom remained on their existing anti-diabetic medications, the absolute change from baseline in HbA1c was -0.5% (p = 0.0013 *vs.* baseline; p = 0.0579 *vs.* placebo) compared to a 0.1% decrease for the corresponding subset of placebo patients. In patients without type 2 diabetes, efruxifermin was associated with a decrease of 0.3% (p = 0.0019 *vs.* baseline; p = 0.0023 *vs.* placebo) compared to an increase of 0.2% for placebo. Efruxifermin appeared to improve glucose control by enhancing peripheral insulin sensitivity, as indicated by a 20% LS mean relative reduction in C-peptide (p = 0.0073 *vs.* baseline) compared to a decrease of 7% for placebo (p = 0.2817 *vs.* placebo). Reflecting the enhanced insulin sensitivity associated with efruxifermin treatment, adiponectin increased by 95.2% (p <0.0001 *vs.* baseline) compared to an increase of 4.1% for placebo (p <0.0001 *vs.* placebo).

As shown in Fig. 5A,B, changes in metabolic parameters (triglycerides, adiponectin, and HbA1c) occurred early and were sustained throughout 16 weeks of treatment.

In contrast to the insulin-sensitizing peroxisome proliferator activating receptor gamma class of oral anti-diabetic medications, efruxifermin treatment was associated with a trend toward reduction in body weight (Fig. 5H) of −2.2 kg (p = 0.0814 *vs.* baseline; p = 0.1325 *vs.* placebo) or a relative loss of 2.2% (p = 0.0628 *vs.* baseline; p = 0.1490 *vs.* placebo), as opposed to a trend toward body weight gain observed in the placebo group of 1.2 kg (absolute CFB, p = 0.5004 *vs.* baseline) or 0.9% (relative CFB, p = 0.6124 *vs.* baseline).

Significant improvement was also noted in liver steatosis assessed by CAP (FibroScan) with an absolute change from baseline (LS mean) of −37.6 dB/m, p = 0.0027 *vs.* placebo (relative CFB: −10.5%; p = 0.0037 *vs.* placebo) in the efruxifermin-treated group compared to an increase of 12.1 dB/m (relative CFB: 4.6%) for the placebo group.

Discussion

Up to 20% of patients with NASH develop cirrhosis (F4) within 10 years.[35] Patients with NASH cirrhosis have a poor prognosis, carrying a high risk of liver decompensation and progression to end-stage liver disease, including ascites, variceal hemorrhage, hepatic encephalopathy, liver failure, and liver cancer,[35] as well as liver-related and all-cause mortality.[9,17] In a recent *post hoc* analysis of two interventional studies, reversal of cirrhosis in patients with NASH, *i.e.* 1-stage improvement of fibrosis, was associated with a greater than 80% reduction in relative risk of liver-related clinical events.[12] Such a reduction in risk represents significant potential medical benefit for patients who may otherwise progress to decompensation and end-stage liver disease. Treatment of this population should aim to prevent hepatic decompensation and liver-related and all-cause mortality, reverse cirrhosis, and ultimately restore liver function. To date, experimental medicines with diverse modes of action have failed to meaningfully improve fibrosis in patients with compensated cirrhosis due to NASH, despite showing promising results in less advanced NASH in prior clinical studies (simtuzumab,[35] selonsertib,[36] cilofexor,[37] firsocostat,[37] semaglutide [NCT03987451], pegbelfermin [Abdelmalek, 2021]).

While patients with compensated cirrhosis due to NASH suffer from more advanced disease, efruxifermin appeared to demonstrate a tolerability and safety profile similar to that reported for patients with less advanced disease (F1–F3 NASH) in the BALANCED main study.[32] Although the trial's duration was not long enough to evaluate clinical outcomes, there were no decompensation events reported for patients with cirrhosis treated with efruxifermin. Circulating biomarkers of liver function (albumin, bilirubin, MELD, C–P scores) appeared unchanged, while most markers of hemostasis were either preserved (platelets, clotting time) or showed a trend towards improvement (PAI-1).

Although this study was not designed to evaluate histologic improvements, an exploratory analysis demonstrated a numeric difference between treated patients compared to placebo following 16 weeks of treatment. Four of 12 patients (33%) treated with 50 mg efruxifermin who opted to receive an end-of-study biopsy showed an improvement in fibrosis stage without worsening of NASH, and an additional 25% showed NASH resolution. Consistent with the apparent improvement in histopathology, liver stiffness was reduced by 26%, a magnitude previously associated with a 1-stage improvement in fibrosis in F3–F4 patients.[36,38] The association between liver stiffness or

JHEP|Reports

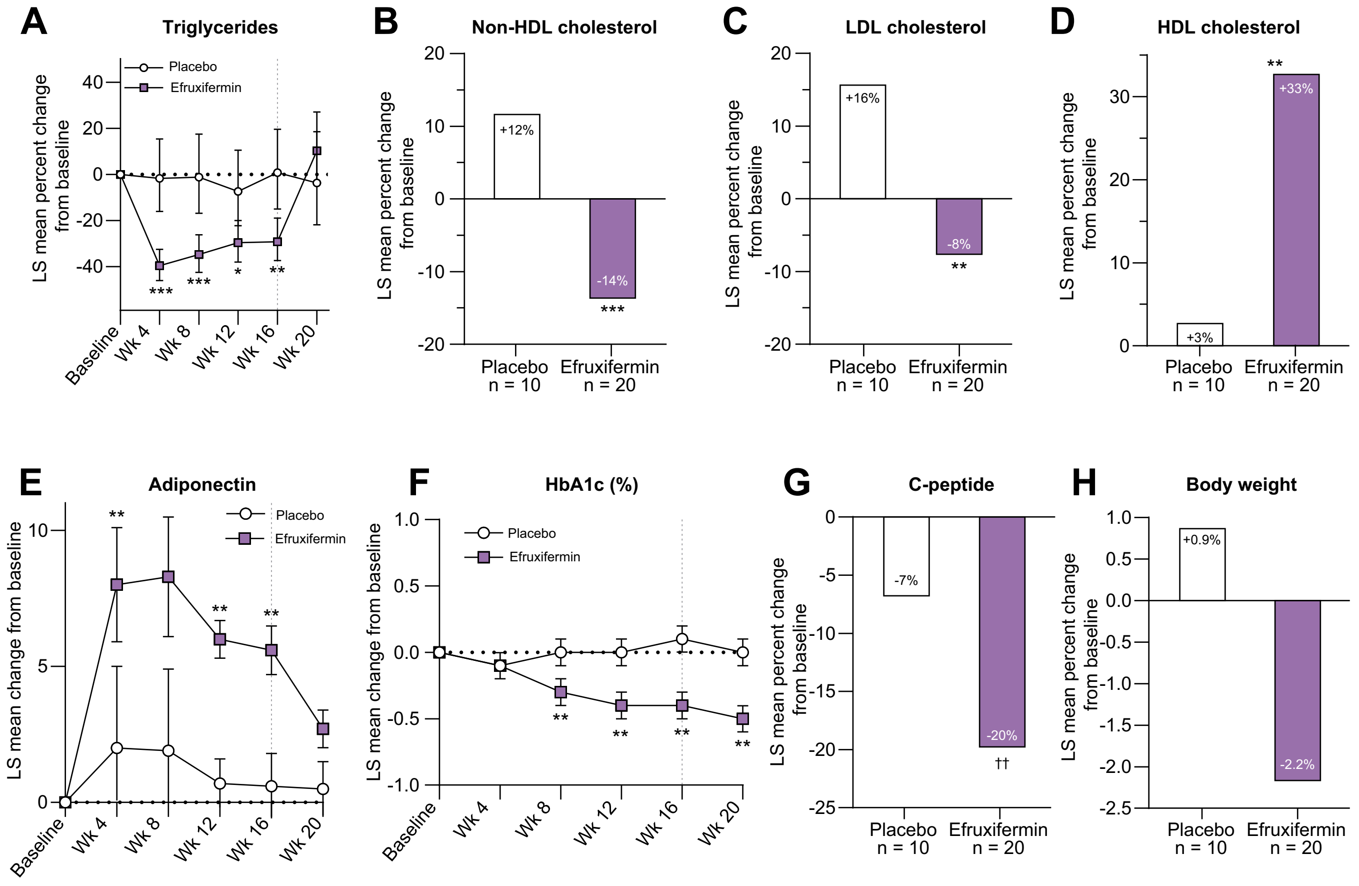

Fig. 5. Mean change (%) from baseline to week 16 of markers of lipid metabolism, glycemic control, and body weight. Relative (A, B, C, D, G, H) or absolute (E, F) change from baseline. ***p <0.0001, **p <0.01, *p <0.05 *vs.* placebo (ANCOVA); ††p <0.01 *vs.* baseline (ANCOVA). ANCOVA, analysis of covariance; HbA1c, hemoglobin A1c.

other non-invasive tests and resolution of features of NASH histopathology remains to be validated.

Along with regression of fibrosis in this small patient cohort, reductions were observed in markers of liver injury and inflammation (ALT, urate, hs-CRP) as well as liver-specific biomarkers of fibrogenesis and fibrosis (Pro-C3, ELF). While decreases over time were also noted in the placebo group for Pro-C3, it is not unusual to see clinical improvement in individuals receiving placebo, possibly due to them adopting a healthier lifestyle when receiving regular clinical evaluation (Hawthorne effect). However, the magnitude of changes in Pro-C3 in efruxifermin-treated patients were significantly larger than for placebo. Another biomarker of fibrogenesis in both soft tissues and bone, P1NP, which is elevated in patients suffering from cirrhosis,[39] was also significantly reduced. As with patients with F1–F3 NASH, the decline in serum markers of fibrogenesis or fibrosis was rapid, reaching a nadir after as few as 4-8 weeks treatment.[32]

In F1–F3 patients, the reduction in serum markers of fibrogenesis and fibrosis preceded the maximal decrease in liver fat,[32] suggesting part of the reduction may be ascribable to a direct antifibrotic action of efruxifermin, as opposed to indirect effects mediated by improvements in liver metabolic health. There is preclinical evidence of direct inhibition of fibrogenesis by FGF21 *in vitro*[26,40,41] and *in vivo*,[42] independent of metabolic improvement. Direct antifibrotic activity is also consistent with the high proportion (50%) of patients with F2/F3 NASH achieving 2-stage regression of fibrosis,[32] as well as reversal of cirrhosis in 33% of patients with cirrhotic NASH after only 16 weeks efruxifermin treatment, despite high baseline levels of collagen present in cirrhosis.[43]

The magnitude of improvement in markers of liver injury (ALT, AST, ALP, GGT, urate) associated with efruxifermin treatment was somewhat smaller for Cohort C than for the BALANCED main study.[32] This may be ascribed to a declining population of hepatocytes as fibrosis progresses to cirrhosis,[14] consistent with the lower baseline levels of these markers in Cohort C compared to the main study. Nevertheless, the overall pattern of improvement in liver histopathology, as well as whole-body and liver metabolic health, is consistent with that observed in patients with F1–F3 NASH.[32]

Improved lipid and glucose metabolism were also observed in an earlier study in patients with type 2 diabetes treated with efruxifermin for 4 weeks.[29] The consistency of responses across three distinct patient populations highlights the reproducible effects of efruxifermin in addressing components of metabolic syndrome. In patients with or without type 2 diabetes, efruxifermin treatment has consistently improved markers of glucose metabolism and insulin sensitivity while restoring a healthy lipoprotein profile without increasing body weight. Should these broad improvements be confirmed with longer term treatment in a larger patient population, efruxifermin could be of

considerable therapeutic utility since type 2 diabetes is frequently inadequately controlled and highly prevalent among patients with NASH, increasing from 30-40% in F1/F2[44–46] to 70-90% in F3/4 NASH.[47]

The time courses for changes in serum markers of metabolism and liver health during 16 weeks of treatment with efruxifermin appeared to reach a maximum after 4-8 weeks, which was maintained through 16 weeks in both F1-3[32] and F4 patients with NASH; this durability will be further assessed in longer-duration, ongoing clinical trials. This pattern contrasts with the waning magnitude of responses for adiponectin and triglyceride during treatment for 24-to-48 weeks with the FGF21 analogue pegbelfermin (Sanyal 2021). A potential explanation could be insufficient agonism by pegbelfermin of the FGF21 receptors (FGFR1c/2c/3c) throughout the dosing interval. Biologically active (*i.e.*, intact C-terminal) pegbelfermin has a reported half-life of approximately one day which appears unlikely to sustain high levels of receptor agonism with once-weekly dosing.[30] In contrast, efruxifermin has a 3–4-day half-life and 2–3.5-day T_{max}.[29] Maintaining high levels of agonism of FGF21's receptors between doses of FGF21 analogues appears necessary to maximize the full range of metabolic improvements. Dosing either efruxifermin or pegozafermin (BIO89-100), which have similar half-lives, once-every-two-weeks was associated with diminished metabolic responses relative to once-weekly dosing.[29,48]

Based on preclinical reports of effects of FGF21 on bone health, the impact of efruxifermin on biomarkers of bone metabolism was evaluated. No changes were observed in CTX-1, a widely reported biomarker of type-I collagen degradation linked to bone resorption. However, decreases were observed in P1NP, a biomarker of type-I collagen synthesis linked to bone formation. While P1NP and CTX-1 have been proposed to be standard markers for evaluating bone health,[39,49,50] type-I collagen is a ubiquitously expressed protein not specific to bone. Active fibrogenesis in soft tissues such as the liver, lungs, or heart contributes significantly to circulating levels of P1NP and C-terminal type-1 collagen extension peptide.[39] In this study, baseline PINP levels were similar in F4 (56.00 ug/L), and F1-3 BALANCED[32] (55.14 ug/L) patients. Given the reduction of liver fibrosis by efruxifermin, as indicated by histology and serum-based markers of soft tissue fibrosis, *i.e.*, ELF score and Pro-C3, decreases in P1NP may be attributable to reduced synthesis of fibrils (type-I and -III collagen) and extracellular matrix (type-I and -IV collagen) in the liver.[39,51] The reduction of P1NP levels associated with efruxifermin may therefore not reflect altered bone turnover, especially in the context of no change in CTX-1.

Limitations to this study include relatively short (16-week) duration of treatment and a small sample size. The observations of acceptable tolerability and safety and encouraging albeit preliminary signs of efficacy will require confirmation in larger and longer-duration studies of efruxifermin. While liver biopsies were obtained from 12 out of 20 efruxifermin- and 5 out of 10 placebo-treated patients, (*i.e.*, all of those who consented to biopsy), the small sample size for evaluating histology precluded statistical analysis. Despite the relatively short treatment period, the pattern and extent of improvement in histopathology was consistent with that seen with F1–F3 patients treated over the same duration of treatment. Moreover, the pattern of improvements in markers of liver health, of liver fibrosis, and of whole-body metabolism was also consistent with that observed in F1–3 patients with NASH.

The breadth of desirable effects in patients with cirrhosis, if confirmed in larger and longer term studies, potentially sets efruxifermin apart from other treatments under development for NASH. Efruxifermin was generally safe and well-tolerated. Improvements in non-invasive markers of liver injury and fibrosis appeared consistent with a trend towards inhibition of fibrogenesis and reversal of cirrhosis, as indicated by a greater number of patients whose fibrosis improved by ≥1-stage on end-of-treatment biopsy. The observed results are encouraging, and further evaluation of efruxifermin as a treatment for compensated cirrhosis due to NASH, in sufficiently powered, longer-duration studies, is warranted.

Abbreviations

ADA(s), anti-drug antibody(ies); AE, adverse event; ALP, alkaline phosphatase; ALT, alanine aminotransferase; ANCOVA, analysis of covariance; AST, aspartate aminotransferase; CFB, change from baseline; C–P, Child-Pugh; CTX-1, C-terminal telopeptide of type 1 collagen; DXA, dual-energy X-ray absorptiometry; ELF, enhanced liver fibrosis; FGF21, fibroblast growth factor-21; FGFR, fibroblast growth factor receptor; GGT, gamma-glutamyltransferase; HbA1c, hemoglobin A1c; HDL-C, HDL-cholesterol; HPA, hypothalamic-pituitary-adrenal; HOMA-IR, homeostatic model assessment of insulin resistance; hs-CRP, high-sensitivity C-reactive protein; INR, international normalized ratio; IRT, interactive response technology; LDL-C, LDL-cholesterol; LS, least squares; MELD, model for end-stage liver disease; NAb, neutralizing antibody; NAFLD, non-alcoholic fatty liver disease; NAS, NAFLD activity score; NASH, non-alcoholic steatohepatitis; Non-HDL-C, non-HDL-cholesterol; PAI-1, plasminogen activator inhibitor-1; P1NP, procollagen type-I N-terminal propeptide; P3NP, procollagen type III N-terminal propeptide; Pro-C3, N-terminal type III collagen propeptide; TEAE, treatment-emergent adverse event; TIMP-1, tissue inhibitor of metalloproteinase-1; ULN, upper limit of normal.

Financial support

This study was funded by Akero Therapeutics.

Conflict of interest

Stephen A. Harrison holds a leadership or fiduciary role at, advises, consults for, receives grants or contracts from Northsea Therapeutics. He advises, consults for, receives grants or contracts, support for attending meetings and/or travel from Madrigal Pharmaceuticals, Inc. He advises, consults for, and receives grants or contracts from Akero Therapeutics, Inc. He advises, consults for, and receives grants or contracts from Axcella Health, Inc., Cymabay Therapeutics, Inc., Galectin Therapeutics, Inc., Hepion Pharmaceuticals, Inc., Hightide Therapeutics, Inc., Intercept Pharmaceuticals, Inc., Metacrine Inc., NGM Biopharmaceuticals Inc., Genfit Corp, Novo Nordisk, Poxel, Sagimet Biosciences. He advises and receives grants or contracts from Gilead Sciences, Inc., Galmed Research & Dev. LTD., and Novartis Pharmaceuticals Corp. He consults for and receives grants or contracts from Viking Therapeutics, Inc. and Enyo Pharma S.A. He advises and consults for Altimmune, Echosens North America Inc. Foresite Labs, LLC, HistoIndex PTE LTD, Medpace Inc., Prometic, Pharma SMT LTD, Ridgeline and Sonic Incytes Medical Corp, Terns Inc. He advises for 89bio, Arrowhead, Chronwell, CiVi, Indalo, PathAI, and Ther-atechnologies. He consults for AgomAB, Alentis Therapeutics AG, Alimentiv, Inc, Boston Pharmaceuticals, B Riley FBR Inc. BVF Partners LP, Cohbar, Inc. Canfite, Corcept Therapeutics, Inc, Fibronostics, Fortress Biotech, Inc GNS, Inipharm, Ionis, Kowa Research Institute, Microba, Nutrasource, Perspectum Diagnostics, and Piper Sandler. He receives

grants or contracts from Cirius Therapeutics, Inc., and CiVi Biopharma Inc. He holds stock or stock options at Akero Therapeutics, Inc., Chronwell Inc., Cirius Therapeutics, Inc, Galectin Therapeutics, Inc., Genfit Corp, Hepion Pharmaceuticals Inc., HistoIndex PTE LTD, Metacrine Inc., NGM Biopharmaceuticals., Northsea Therapeutics B.V, and Sonic Incytes Medical Corp. Peter J. Ruane: none. Bradley Freilich: none. Guy Neff has received payment or honoraria from Intercept Pharmaceuticals. Rashmee Patil has received grants to institution from 89 Bio, AltImmune, Boehringer Ingelheim, Bristol Myers Squibb, Corcept Therapeutics, Fibronostics, Galectin Therapeutics, Genentech, Gilead, Helio Health, Hepagene, Madrigal Pharmaceuticals, NGMBio, NorthSea Therapeutics, Poxel, Sagimet Biosciences, and Viking Therapeutics. He consults for Intercept Pharmaceuticals. Cynthia Behling has received payment via Pacific Rim Pathology for liver biopsy scoring. She has received grants as a co-investigator under N100 K U01 DK61734. She has received honoraria for lectures from Pfizer and Alimentev. She is a co-chair on NASH CRN Pathology Committee. Chen Hu: none. Reshma Shringarpure, Brittany de Temple, Erica Fong, Erik J. Tillman, Timothy Rolph, Andrew Cheng, and Kitty Yale are employees of Akero Therapeutics and own stock and/or stock options of Akero Therapeutics.

Please refer to the accompanying ICMJE disclosure forms for further details.

Authors' contributions

The protocol was designed by KY, TR, BdT, EF, AC, HC, SH. Data were acquired by SH, PJR, BF, GF, RP, CB.The data were analyzed by KY, TR, BdT, EF, AC, SH, EJT, RS, HC. A statement indicating that at least one author had access to all of the data and can vouch for the integrity of the data analyses [KY].

Data availability statement

The datasets generated and/or analyzed in this study are considered commercially sensitive and, therefore, are not publicly available. Requests for data supporting findings in the manuscript should be made to the corresponding author and will be reviewed individually. Data might be shared in the form of aggregate data summaries and via a data transfer agreement. Individual participant-level raw data containing confidential or identifiable patient information are subject to patient privacy and cannot be shared.

Acknowledgements

The initial draft of the manuscript was prepared by Andrea R. Gwosdow, Ph.D. of Gwosdow Associates Science Consultants, LLC on behalf of Akero Therapeutics.

Supplementary data

Supplementary data to this article can be found online at https://doi.org/10.1016/j.jhepr.2022.100563.

References

[1] Matteoni CA, Younossi ZM, Gramlich T, Boparai N, Liu YC, McCullough AJ. Nonalcoholic fatty liver disease: a spectrum of clinical and pathological severity. Gastroenterology 1999;116:1413–1419.
[2] Takahashi Y, Fukusato T. Histopathology of nonalcoholic fatty liver disease/nonalcoholic steatohepatitis. World J Gastroenterol 2014;20:15539–15548.
[3] Chalasani N, Younossi Z, Lavine JE, Charlton M, Cusi K, Rinella M, et al. The diagnosis and management of nonalcoholic fatty liver disease: practice guidance from the American Association for the Study of Liver diseases. Hepatology 2018;67:328–357.
[4] Murag S, Ahmed A, Kim D. Recent epidemiology of nonalcoholic fatty liver disease. Gut Liver 2021;15:206–216.
[5] Younossi ZM, Blissett D, Blissett R, Henry L, Stepanova M, Younossi Y, et al. The economic and clinical burden of nonalcoholic fatty liver disease in the United States and Europe. Hepatology 2016;64:1577–1586.
[6] Le MH, Yeo YH, Li X, Li J, Zou B, Wu Y, et al. 2019 global NAFLD prevalence: a systematic review and meta-analysis. Clin Gastroenterol Hepatol 2021. S1542-3565(21)01280-01285, https://doi.org/10.1016/j.cgh.2021.12.002.
[7] Ekstedt M, Hagström H, Nasr P, Fredrikson M, Stål P, Kechagias S, et al. Fibrosis stage is the strongest predictor for disease-specific mortality in NAFLD after up to 33 years of follow-up. Hepatology 2015;61:1547–1554.
[8] Angulo P, Kleiner DE, Dam-Larsen S, Adams LA, Bjornsson ES, Charatcharoenwitthaya P, et al. Liver fibrosis, but no other histologic features, is associated with long-term outcomes of patients with nonalcoholic fatty liver disease. Gastroenterology 2015;149:389–397.
[9] Dulai PS, Singh S, Patel J, Soni M, Prokop LJ, Younossi Z, et al. Increased risk of mortality by fibrosis stage in nonalcoholic fatty liver disease: systematic review ad meta-analysis. Hepatology 2017;65:1557–1565.
[10] Sanyal AJ. Past, present and future perspectives in nonalcoholic fatty liver disease. Nat Rev Gastroenterol Hepatol 2019;16:377–386.
[11] Taylor RS, Taylor RJ, Bayliss S, Hagström H, Nasr P, Schattenberg JM, et al. Association between fibrosis stage and outcomes of patients with nonalcoholic fatty liver disease: a systematic review and meta-analysis. Gastroenterology 2020;158:1611–1625.
[12] Sanyal AJ, Anstee QM, Trauner M, Lawitz EJ, Abdelmalek MF, Ding D, et al. Cirrhosis regression is associated with improved clinical outcomes in patients with nonalcoholic steatohepatitis. Hepatology 2022;75:1235–1246.
[13] van der Poorten D, Samer CF, Ramezani-Moghadam M, Coulter S, Kacevs ka M, Schrijnders D, et al. Hepatic fat loss in advanced nonalcoholic steatohepatitis: are alterations in serum adiponectin the cause? Hepatology 2013;57:2180–2188.
[14] Heyens LJM, Busschots D, Koek GH, Robaeys G, Francque S. Liver fibrosis in non-alcoholic fatty liver disease: from biopsy to non-invasive biomarkers in diagnosis and treatment. Front Med 2021;8:615978.
[15] Noureddin M, Vipani A, Bresse C, Todo T, Kim IK, Alkhouri N, et al. NASH leading cause of liver transplant in women: updated analysis of indications for liver transplant and ethnic and gender variances. Am J Gastroenterol 2018;113:1649–1659.
[16] FDA Guidance. Nonalcoholic steatohepatitis with compensated cirrhosis: developing drugs for treatment guidance for industry. 2019; https://www.fda.gov/regulatory-information/search-fda-guidance-documents/nonalcoholic-steatohepatitis-compensated-cirrhosis-developing-drugs-treatment-guidance-industry. [Accessed 26 August 2022].
[17] Albhaisi S, Sanyal A. Recent advances in understanding and managing non-alcoholic fatty liver disease. F1000 Res 2018;7:720.
[18] Lin Z, Tian H, Lam KSL, Lin S, Hoo RCL, Konishi M, et al. Adiponectin mediates the metabolic effects of FGF21 on glucose homeostasis and insulin sensitivity in mice. Cell Metab 2013;17:779–789.
[19] Camporez JPG, Jornayvaz FR, Petersen MC, Pesta D, Guigni BA, Serr J, et al. Cellular mechanisms by which FGF21 improves insulin sensitivity in male mice. Endocrinology 2013;154:3099–3109.
[20] Mu J, Pinkstaff J, Li Z, Skidmore L, Li N, Myler H, et al. FGF21 analogs of sustained action enabled by orthogonal biosynthesis demonstrate enhanced antidiabetic pharmacology in rodents. Diabetes 2012;61:505–512.
[21] Xu J, Lloyd DJ, Hale C, Stanislaus S, Chen M, Sivits G, et al. Fibroblast growth factor 21 reverses hepatic steatosis, increases energy expenditure, and improves insulin sensitivity in diet-induced obese mice. Diabetes 2009;58:250–259.
[22] Coskun T, Bina HA, Schneider MA, Dunbar JD, Hu CC, Chen Y, et al. Fibroblast growth factor 21 corrects obesity in mice. Endocrinology 2008;149:6018–6027.
[23] Kim AM, Somayaji VR, Dong JQ, Rolph TP, Weng Y, Chabot JR, et al. Once-weekly administration of a long-acting fibroblast growth factor 21 analogue modulates lipids, bone turnover markers, blood pressure and body weight differently in obese people with hypertriglyceridaemia and in non-human primates. Diabetes Obes Metab 2017;19:1762–1772.
[24] Wu G, Li H, Fang Q, Zhang J, Zhang M, Zhang L, et al. Complementary role of fibroblast growth factor 21 and cytokeratin 18 in monitoring the different stages of nonalcoholic fatty liver disease. Sci Rep 2017;7:5095.
[25] Talukdar S, Zhou Y, Li D, Rossulek M, Dong J, Somayaji V, et al. A long-acting FGF21 molecule, PF-05231023, decreases body weight and improves lipid profile in non-human primates and type 2 diabetic subjects. Cell Metab 2016;23:427–440.
[26] Tillman EJ, Rolph T. FGF21: an emerging therapeutic target for non-alcoholic steatohepatitis and related metabolic diseases. Front Endocrinol 2020;11:601290.
[27] Fisher FM, Chui PC, Nasser IA, Popov Y, Cunniff JC, Lundasen T, et al. Fibroblast growth factor 21 limits lipotoxicity by promoting hepatic fatty

acid activation in mice on methionine and choline-deficient diets. Gastroenterology 2014;147:1073–1083.
[28] Charles ED, Neuschwander-Tetri BA, Pablo Frias J, Kundu S, Luo Y, Tirucherai GS, et al. Pegbelfermin (BMS-986036), PEGylated FGF21, in patients with obesity and Type 2 diabetes: results from a randomized phase 2 study. Obesity (Silver Spring) 2019;27:41–49.
[29] Kaufman A, Abuqayyas L, Denney WS, Tillman EJ, Rolph T. AKR-001, an Fc-FGF21 analog, showed sustained pharmacodynamic effects on insulin sensitivity and lipid metabolism in type 2 diabetes patients. Cell Rep Med 2020;1:100057.
[30] Tirucherai GS, Mora J, Revankar R, Charles ED. Pharmacokinetics and safety of pegbelfermin (BMS-986036) administered in the abdomen and upper arm to normal, overweight, and obese healthy participants. J Hepatol 2019;70:E798.
[31] Stanislaus S, Hecht R, Yie J, Hager T, Hall M, Spahr C, et al. A novel Fc-FGF21 with improved resistance to proteolysis, increased affinity toward β-Klotho, and enhanced efficacy in mice and cynomolgus monkeys. Endocrinology 2017;158:1314–1327.
[32] Harrison SA, Ruane PJ, Freilich BL, Neff G, Patil R, Behling CA, et al. Efruxifermin in non-alcoholic steatohepatitis: a randomized, double-blind, placebo-controlled phase 2a trial. Nat Med 2021;27:1262–1271.
[33] FDA Guidance. Immunogenicity testing of therapeutic protein products-developing and validating assays for anti-drug antibody detection. 2019; https://www.fda.gov/regulatory-information/search-fda-guidance-documents/immunogenicity-testing-therapeutic-protein-products-developing-and-validating-assays-anti-drug. [Accessed 26 August 2022].
[34] Newsome PN, Sasso M, Deeks JJ, Paredes A, Boursier J, Chan W-K, et al. FibroScan-AST (FAST) score for the non-invasive identification of patients with non-alcoholic steatohepatitis with significant activity and fibrosis: a prospective derivation and global validation study. Lancet Gastroenterol Hepatol 2020;5:362–373.
[35] Harrison SA, Abdelmalek MF, Caldwell C, Shiffman ML, Diehl AM, Ghalib R, et al. Simtuzumab is ineffective for patients with bridging fibrosis or compensated cirrhosis caused by nonalcoholic steatohepatitis. Gastroenterology 2018;155:1140–1153.
[36] Harrison SA, Wong VW-S, Okanoue T, Bzowej N, Vuppalanchi R, Younes Z, et al. Selonsertib for patients with bridging fibrosis or compensated cirrhosis due to NASH: results from randomized phase III STELLAR trials. J Hepatol 2020;73:26–39.
[37] Dufour J-F, Caussy C, Loomba R. Combination therapy for non-alcoholic steatohepatitis: rationale, opportunities and challenges. Gut 2020;69:1877–1884.
[38] Anstee QM, Lawitz EJ, Alkhouri N, Wong VW-S, Romero-Gomez M, Okanoue T, et al. Noninvasive tests accurately identify advanced fibrosis due to NASH: baseline data from the STELLAR trials. Hepatology 2019;70:1521–1530.
[39] Szulc P. Bone turnover: biology and assessment tools. Best Prac Res Clin Endocrinol Metabol 2018;32:725–738.
[40] Xu P, Zhang Y, Liu Y, Yuan Q, Song L, Liu M, et al. Fibroblast growth factor 21 attenuates hepatic fibrogenesis through TGF-β/smad2/3 and NF-κB signaling pathways. Toxicol Appl Pharmacol 2016;290:43–53.
[41] Opoku YK, Liu Z, Afrifa J, Kumi AK, Liu H, Ghartey-Kwansah G, et al. Fibroblast Growth Factor-21 ameliorates hepatic encephalopathy by activating the $STAT_3$-$SOCS_3$ pathway to inhibit activated hepatic stellate cells. EXCLI J 2020;19:567–581.
[42] Zhang S, Yu D, Wang M, Huang T, Wu H, Zhang Y, et al. FGF_{21} attenuates pulmonary fibrogenesis through ameliorating oxidative stress in vivo and in vitro. BioMed Pharmacother 2018;103:1516–1525.
[43] Masugi Y, Abe T, Tsujikawa H, Effendi K, Hashiguchi A, Abe M, et al. Quantitative assessment of liver fibrosis reveals a nonlinear association with fibrosis stage in nonalcoholic fatty liver disease. Hepatol Comm 2018;2:58–68.
[44] Harrison SA, Neff G, Guy CD, Bashir MR, Paredes AH, Frias JP, et al. Efficacy and safety of aldafermin, an engineered FGF19 analog, in a randomized, double-blind, placebo-controlled trial of patients with nonalcoholic steatohepatitis. Gastroenterology 2021;160:219–231.
[45] Francque SM, Bedossa P, Ratziu V, Anstee QM, Bugianesi E, Sanyal AJ, et al. A randomized, controlled trial of the Pan-PPAR agonist lanifibranor in NASH. N Engl J Med 2021;385:1547–1558.
[46] Harrison SA, Gawrieh S, Roberts K, Lisanti CJ, Schwope RB, Cebe KM, et al. Prospective evaluation of the prevalence of non-alcoholic fatty liver disease and steatohepatitis in a large middle-aged US cohort. J Hepatol 2021;75:284–291.
[47] Abdelmalek MF, Charles ED, Sanyal AJ, Harrison SA, Neuschwander-Tetri BA, Goodman Z, et al. The FALCON program: two phase 2b randomized, double-blind, placebo-controlled studies to assess the efficacy and safety of pegbelfermin in the treatment of patients with nonalcoholic steatohepatitis and bridging fibrosis or compensated cirrhosis. Contemp Clin Trials 2021;104:106335.
[48] Frias JP, Lawitz EJ, Ortiz-LaSanta G, Franey B, Morrow L, Chen CY, et al. BIO89-100 demonstrated robust reductions in liver fat and liver fat volume (LFV) by MRI-PDFF, favorable tolerability and potential for weekly (QW) or every 2 weeks (Q2W) dosing in a phase 1b/2a placebo-controlled, double-blind, multiple ascending dose study in NASH. J Endocr Soc 2021;5(Suppl 1):A5–A6.
[49] Chavassieux P, Portero-Muzy N, Roux JP, Garnero P, Chapurlat R. Are biochemical markers of bone turnover representative of bone histomorphometry in 370 postmenopausal women? J Clin Endocrinol Metab 2015;100:4662–4668.
[50] Bhattoa HP, Cavalier E, Eastell R, Heijboer AC, Jørgensen NR, Makris K, et al. Analytical considerations and plans to standardize or harmonize assays for the reference bone turnover markers PINP and β-CTX in blood. Clin Chim Acta 2021;515:16–20.
[51] Veidal SS, Vassiliadis E, Bay-Jensen AC, Tougas G, Vainer B, Karsdal MA. Procollagen type I N-terminal propeptide (PINP) is a marker for fibrogenesis in bile duct ligation-induced fibrosis in rats. Fibrogen Tissue Repair 2010;3:5. https://doi.org/10.1186/1755-1536-3-5.

Journal of Hepatology, Volume ■

Supplemental information

A randomized, double-blind, placebo-controlled phase IIa trial of efruxifermin for patients with compensated NASH cirrhosis

Stephen A. Harrison, Peter J. Ruane, Bradley Freilich, Guy Neff, Rashmee Patil, Cynthia Behling, Chen Hu, Reshma Shringarpure, Brittany de Temple, Erica Fong, Erik J. Tillman, Timothy Rolph, Andrew Cheng, and Kitty Yale

A randomized, double-blind, placebo-controlled phase IIa trial of efruxifermin for patients with compensated NASH cirrhosis

Stephen A. Harrison, Peter Ruane, Bradley Freilich, Guy Neff, Rashmee Patil, Cynthia Behling, Chen Hu, Reshma Shringarpure, Brittany de Temple, Erica Fong, Erik J Tillman, Timothy Rolph, Andrew Cheng, Kitty Yale

Table of contents

Fig. S1 2

Table S1 3

Table S2 8

Table S3 10

Table S4 11

Table S5 12

Supplementary Figures

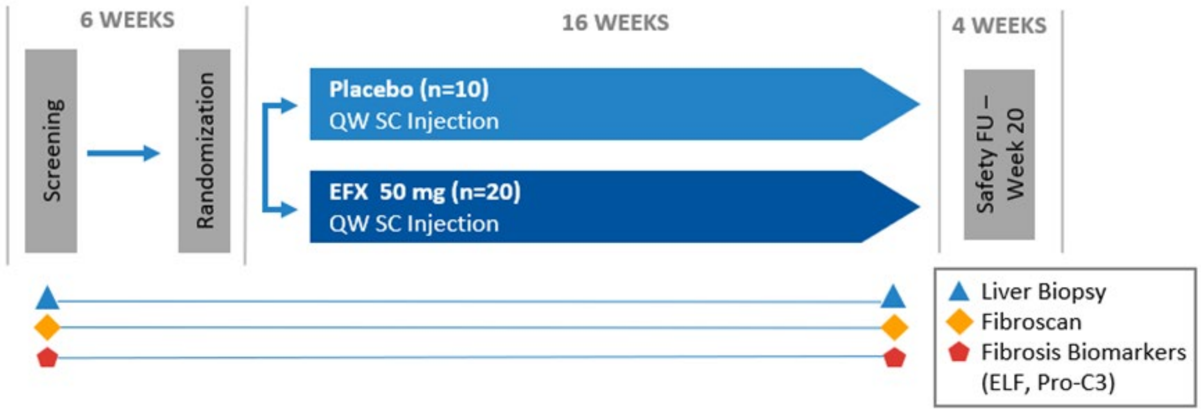

Fig. S1. Cohort C study design.

Table S1. Patient Inclusion and Exclusion Criteria

Inclusion Criteria	Exclusion Criteria
Males or non-pregnant, non-lactating females between 18 to 80 years of age inclusive, based on the date of the screening visit	Weight gain or loss >5% in the 3 months prior to randomization or >10% in the 6 months prior to screening
Documented compensated cirrhosis (F4) due to NASH within 24 months of screening with no evidence of competing etiology, and at least 1 co-existing or historic metabolic comorbidity. If a historical biopsy is not available within 24 months of screening, the participant must undergo a liver biopsy that was assessed by the local or central reader and determined to have compensated cirrhosis (F4).	Type 1 and insulin-dependent Type 2 diabetes
FibroScan measurement >13.1 kPa	Prior history of decompensated liver disease including ascites, hepatic encephalopathy, or variceal bleeding
Enhanced liver fibrosis (ELF) Test score of >9.8	Poorly controlled hypertension, blood pressure >160/100
Must have no evidence of worsening of ALT and AST (within 50%) measurements at the screening (-6 weeks) and pre-baseline (-2 weeks) visits	Chronic hepatitis B virus infection (hepatitis B surface antigen positive)
Screening laboratory parameters, as determined by the central laboratory:<br>○ Estimated glomerular filtration rate (eGFR) ^{3}60 mL/min, as calculated by the Cockcroft-Gault equation;<br>○ HbA1c) ≤9.5% (or serum fructosamine ≤381 µmol if HbA1c measurement was unavailable);	Chronic hepatitis C virus (HCV) infection (HCV antibody and HCV ribonucleic acid [RNA] positive). Participants cured of HCV infection less than 2 years prior (based on date of RNA PCR negative confirmation following conclusion of treatment) to the screening visit were not eligible

○ Hemoglobin ≥11 g/dL; International normalized ratio (INR) ≤1.3, unless due to therapeutic anticoagulation;<br>○ Direct bilirubin ≤0.3 mg/dL;<br>○ Total bilirubin ≤1.3 × upper limit of normal (ULN), unless due to an alternate etiology such as Gilbert's syndrome or hemolytic anemia;<br>○ Creatinine kinase <3 × ULN;<br>○ Platelet count ≥125,000/µL;<br>○ Serum triglyceride level ≤500 mg/dL;<br>○ Alanine aminotransferase (ALT) <5 × ULN;<br>○ Aspartate aminotransferase (AST) <5 × ULN;<br>○ Alkaline phosphatase (ALP) <2 × ULN;<br>○ Albumin ≥3.5 g/dL	
Participants on Vitamin E ≥400 IU/day, thiazolidinediones (including, but not limited to, pioglitazone, rosiglitazone, and lobeglitazone), glucagon-like peptide 1 (GLP1), or sodium-glucose transport protein 2 (SGLT2) must be on a stable dose (defined as no changes in prescribed dose, new medications, or discontinuation) for at least 6 months prior to screening	Prior or planned (during the study period) bariatric surgery (eg, gastroplasty, Roux-en-Y gastric bypass). Surgery reversal or removal of intragastric balloon >2 years prior to enrollment would be eligible
Participants on antidiabetic, weight loss, or lipid-modifying medication(s) must be on stable dose(s) for at least 3 months prior to screening	Other causes of liver disease based on medical history and/or centralized review of liver histology, including but not limited to: alcoholic liver disease, autoimmune disorders (eg, primary biliary cholangitis, primary sclerosing cholangitis, autoimmune

	hepatitis), drug-induced hepatotoxicity, Wilson disease, clinically significant iron overload, or alpha-1-antitrypsin deficiency requiring treatment
Willing and able to give written informed consent prior to any study specific procedures being performed	Participants with osteoporosis, defined as a T-score of -2.5 or lower at screening
Female participants of childbearing potential must have a negative pregnancy test at screening and baseline/day 1	History of liver transplantation
Male and female participants of childbearing potential who engaged in heterosexual intercourse must agree to use protocol specified method(s) of contraception as described in Appendix C of the Study Protocol	Current or prior history of hepatocellular carcinoma
	Alcohol intake above an average limit of 2 drinks per day for women and 3 drinks per day for men as assessed by the AUDIT-C questionnaire
	Human immunodeficiency virus infection
	Unstable cardiovascular disease. Any acute cardiovascular event in the six months prior to screening (eg, myocardial infarction, cerebral vascular accident, symptomatic rhythm disturbance [stable, controlled atrial fibrillation is allowed]); or an emergency room visit or hospitalization for confirmed cardiovascular disease
	Life expectancy less than 2 years
	Participants taking fibrates and peroxisome proliferator-activated receptor alpha agents

	(clofibrate, gemfibrozil, ciprofibrate, bezafibrate, or fenofibrate)
	Use of any investigational medication within 30 days or within 5 half-lives prior to screening
	Participants with a history of (12 months prior to screening) or current use of prescription drugs associated with liver steatosis (eg, methotrexate, amiodarone, high-dose estrogen, tamoxifen, systemic steroids, anabolic steroids, valproic acid). Short courses of corticosteroids (less than 2 weeks) may be allowed prior to screening if in the investigator's opinion they are not associated with liver steatosis or clinically relevant
	Positive urine drug screen for amphetamines, cocaine, or opiates (eg, fentanyl, heroin, morphine) at screening. Subjects on stable methadone or buprenorphine maintenance treatment for at least 6 months prior to screening may be included in the study. Participants with a positive urine drug screen due to prescription medication (eg, opiates, methylphenidate) are eligible if the prescription and diagnosis are reviewed and approved by the investigator
	Concurrent participation in another therapeutic clinical study

	Presence of any laboratory abnormality or significant systemic or major illnesses (other than liver disease) that, in the opinion of the investigator, compromise the participant's ability to safely participate in and complete the study including:<br>○ Pulmonary disease, renal failure, organ transplantation, serious psychiatric disease, malignancy, history of substance abuse and/or a psychiatric condition requiring hospitalization and/or emergency room visit within six months of screening
	Unavailable for follow-up assessment or concern for participant's compliance with the protocol procedures
	History of significant pancreatic disorders (acute pancreatitis, chronic pancreatitis, hereditary pancreatitis, pancreatic cancer)
	Child-Pugh score >6 (Class B or C), as determined at screening, unless due to therapeutic anti-coagulation
	Model for End Stage Liver Disease score >12, as determined at screening, unless due to therapeutic anti-coagulation

Table S2. Exploratory Endpoints

Change from baseline in markers of liver injury and function, including alanine aminotransferase (ALT), aspartate aminotransferase (AST), alkaline phosphatase (ALP), gamma glutamyl transferase (GGT), bilirubin, International Normalized Ratio (INR), albumin, Model for End-Stage Liver Disease (MELD) score, Child-Pugh (C-P) score, and urate at weeks 12, 16, and 20
Change from baseline in body weight at weeks 12, 16, and 20
Change from baseline in bone mineral density of the lumbar spine, femoral neck, and total hip as measured by dual energy x-ray absorptiometry (DXA) at week 16
The proportion of participants whose fibrosis regressed by ≥1 stage according to the NASH Clinical Research Network (CRN) classification at week 16
The proportion of participants who had no worsening in NAS with improvement in fibrosis stage at week 16
The proportion of participants who had ≥2-point improvement in NAS at week 16
Change from baseline in platelets at weeks 12, 16, and 20
The proportion of participants who had NASH resolution (0- to 1-point inflammation, 0-point ballooning) at week 16
The proportion of participants who had fibrosis improvement ≥2 stages at week 16
Change from baseline in neo-epitope of matrix metalloproteinase-9 mediated degradation of type III collagen (C3M)
Change from baseline in lipoprotein profile and markers of lipid metabolism, insulin sensitivity, and glycemic control at weeks 12, 16, and 20
The incidence and severity of any decompensated liver disease events including ascites, hepatic encephalopathy, or variceal bleeding
Change from baseline in liver steatosis as evaluated by FibroScan (CAP) at week 16
The following endpoints are not reported in this publication and may be included in a future publication:
Change from baseline in body fat distribution
Plasma concentrations of efruxifermin
Change from baseline in health-related quality of life (HRQoL) at weeks 12 and 16
Change from baseline in PRO-C3/C3M ratio

The proportion of participants who had ≥1-point improvement in NAS, fibrosis, steatosis, lobular inflammation, or hepatocellular ballooning at week 16
Change from baseline in waist-to-hip ratio at weeks 12, 16, and 20

Table S3. Baseline demographics and key disease characteristics in the Liver Biopsy Analysis Set (LBAS; N=17)

Parameter	Placebo (n=5)	Efruxifermin (n=12)
**Mean age, years (SD)**	56.4 (9.8)	62.7 (8.5)
**Sex, n (%)**		
Male	3 (60.0)	3 (25.0)
**Race or ethnicity, n (%)**		
White	5 (100.0)	12 (100.0)
Black or African American	0	0
Native Hawaiian or other Pacific Islander	0	0
Hispanic or Latino	0	6 (50.0)
**Metabolic risk factors and parameters, mean (SD)**		
Body weight, kg	118.1 (8.9)	94.0 (18.4)
Body mass index, kg/m^2	39.0 (4.1)	34.7 (4.3)
Type 2 diabetes, n (%)	2 (40.0)	6 (50.0)
**Markers of liver health, mean (SD)**		
Alanine aminotransferase (ALT), U/L	41.2 (25.5)	31.9 (19.1)
Aspartate aminotransferase (AST), U/L	38.6 (27.4)	32.9 (17.0)
Gamma glutamyl transferase (GGT), U/L	49.0 (17.1)	77.3 (38.8)
Liver steatosis, CAP[a] score, dB/m	346.8 (37.0)	282.3 (53.0)
**Markers of fibrosis, mean (SD)**		
Pro-C3, ug/L	28.7 (13.6)	28.4 (35.1)
ELF score	10.1 (1.0)	10.5 (1.2)
Liver stiffness, kPA	24.4 (5.6)	25.4 (12.7)
**Markers of liver disease severity, mean (SD)**		
Model for end stage liver disease (MELD) score	6.8 (1.1)	7.6 (1.2)
Child-Pugh (C-P) score	5.0 (0.0)	5.1 (0.3)
**Markers of lipid metabolism, mean (SD)**		
Total cholesterol, mg/dL	182.4 (34.0)	158.8 (46.2)
Triglycerides, mg/dL	135.3 (82.0)	127.7 (62.8)
LDL-C, mg/dL	111.1 (20.8)	86.4 (39.9)
HDL-C, mg/dL	44.2 (12.6)	46.8 (8.9)
Non-HDL-C, mg/dL	138.2 (36.1)	112.0 (46.9)
**Markers of glycemic control, mean (SD)**		
HbA1c, %	6.6 (1.8)	5.9 (0.7)
**Concomitant medications of interest, n (%)**		
Statins	0	4 (33.3)

[a]CAP: Controlled Attenuation Parameter;

Table S4. Change from baseline to week 16 in noninvasive markers of fibrosis in the Liver Biopsy Analysis Set (LBAS; N=17)

	**Placebo (n=5)**		**Efruxifermin (n=12)**		p vs placebo
	Value	p vs baseline	Value	p vs baseline	
**Liver Stiffness (kPa)**					
Baseline mean (SD)	24.4 (5.6)		25.4 (12.7)		
LS mean absolute CFB (95% CI)	**0.3** (-8.5, 9.2)	0.9369	**-5.3** (-11.0, 0.4)	0.0679	0. 2734
LS mean percent CFB (95% CI)	**-0.0** (-26.2, 26.2)	0.9976	**-20.6** **(-37.6, -3.7)**	0.0203	0.1788
**Pro-C3 (µg/L)**					
Baseline mean (SD)	28.7 (13.6)		28.4 (35.1)		
LS mean absolute CFB (95% CI)	**1.3** (-3.8, 6.4)	0.5952	**-13.5** (-16.8, -10.2)	<0.0001	0.0001
LS mean percent CFB (95% CI)	**12.3** (-5.4, 30.0)	0.1584	**-23.0** (-34.4, -11.6)	0.0007	0.0029
**ELF Score**					
Baseline, mean (SD)	**10.1 (1.0)**		**10.5 (1.2)**		
LS Mean absolute change from baseline (95% CI)	0.2 **(-0.3, 0.7)**	0.4638	- 0.6 **(-0.9, -0.3)**	0.0015	0.0162

Table S5. Change from baseline for liver function and hemostasis parameters after 16 weeks of efruxifermin treatment.

Parameter	Placebo			Efruxifermin			
	**Baseline Mean (SD) N=10**	**Absolute CFB[a,b] N=10**	**p vs baseline**	**Baseline Mean (SD) N=20**	**Absolute CFB[a,b] N=19**	**p vs baseline**	**p vs placebo**
Platelets	**166.8 (29.5)**	**-5.0 (6.4)**	**0.4433**	**193.1 (34.8)**	**3.7 (4.5)**	**0.4252**	**0.2943**
Bilirubin	**0.8 (0.2)**	**0.0 (0.1)**	**0.8360**	**0.7 (0.3)**	**0.0 (0.0)**	**0.5702**	**0.8694**
Albumin	**4.3 (0.2)**	**0.0 (0.1)**	**0.5351**	**4.2 (0.3)**	**-0.1 (0.0)**	**0.0178**	**0.0608**
Fibrinogen	**380.2 (42.6)**	**-33.66**	**0.1228**	**458.7 (59.2)**	**29.3**	**0.0580**	**0.0234**
INR[c]	**1.1 (0.1)**	**0.0 (0.0)**	**0.6777**	**1.1 (0.1)**	**0.0 (0.0)**	**0.9816**	**0.7278**
PAI-1[d]	**13.7 (12.4)**	**0.67 (2.4)**	**0.7806**	**15.1 (10.5)**	**-8.74 (1.7)**	**<0.0001**	**0.0033**
MELD[e]	**6.9 (0.9)**	**0.3 (0.4)**	**0.4022**	**7.4 (1.1)**	**0.0 (0.3)**	**0.8884**	**0.5527**
Child-Pugh	**5.0 (0)**	**0.0**	**NE[f]**	**5.1 (0.2)**	**0.0**	**NE**	**NE**

[a]CFB: Change from baseline; [b]Data are LS means (SE); [c]INR: International Normalized Ratio; [d]PAI-1: Plasminogen-Activator Inhibitor-1; [e]MELD: Model for End Stage Liver Disease; [f]NE: Not estimable
