# Supplementary material for: A randomized, double-blind, placebo-controlled phase IIa trial of efruxifermin for patients with compensated NASH cirrhosis
Source: JHEP Rep. 2022 Aug 23;5(1):100563. doi: 10.1016/j.jhepr.2022.100563 (PMC9832280; doi:10.1016/j.jhepr.2022.100563)
Supplement: Multimedia component 3 [file mmc3.pdf]

CLINICAL STUDY PROTOCOL:
A PHASE 2A, RANDOMIZED, DOUBLE-BLIND, PLACEBO-CONTROLLED STUDY EVALUATING THE SAFETY AND EFFICACY OF EFRUXIFERMIN IN SUBJECTS WITH NONALCOHOLIC STEATOHEPATITIS (NASH)

Protocol Number:	AK-US-001-0101
IND Number:	140307
EudraCT Number:	Not Applicable

CRO Medical Monitor: David Schneider, MD MBA
Vice President Medical, Medical Department
Medpace
5365 Medpace Way
Cincinnati, Ohio 45227
Tel: +1.513.579.9911, ext. 16244
Mobile: +1.513.846.2500
Fax: +1.513.579.0444
E-mail: d.schneider@medpace.com

Sponsor: Akero Therapeutics, Inc
601 Gateway Blvd., Suite 350
South San Francisco, CA 94080

Version and Date: Amendment 6, 08 October 2020

Confidentiality Statement

Information contained in this protocol is proprietary and confidential in nature and may not be used, divulged, published, or otherwise disclosed to others except to the extent necessary to obtain approval of the Institutional Review Board or as required by law. Persons to whom this information is disclosed should be informed that this information is confidential and may not be further disclosed without the express permission of Akero Therapeutics, Inc.

STUDY ACKNOWLEDGEMENT SIGNATURE PAGE

A Phase 2a, Randomized, Double-Blind, Placebo-Controlled Study Evaluating the Safety and Efficacy of Efruxifermin in Subjects with Nonalcoholic Steatohepatitis (NASH)

AK-US-001-0101, Amendment 6, 08 October 2020

This clinical study protocol was subject to critical review and has been approved by the Sponsor.

Kitty Yale — October 9th 2020

Kitty Yale
Chief Development Officer, Akero Therapeutics, Inc.

Date

INVESTIGATOR STATEMENT

I have read the protocol, including all appendices, and I agree that it contains all necessary details for me and my staff to conduct this study as described. I will conduct this study as outlined herein and will make a reasonable effort to complete the study within the time designated.

I will provide all study personnel under my supervision copies of the protocol and access to all information provided by Akero Therapeutics, Inc. I will discuss this material with them to ensure that they are fully informed about the drugs and the study.

Principal Investigator Name (Printed) ____________________

Signature ____________________

Date ____________________

SYNOPSIS

Name of Sponsor Company: Akero Therapeutics, Inc.

Name of Test Product and Dosage: EFX 70 mg, 50 mg, and 28 mg

Title of Study:

A Phase 2a, Randomized, Double-Blind, Placebo-Controlled Study Evaluating the Safety and Efficacy of EFX in Subjects with Nonalcoholic Steatohepatitis (NASH)

Investigator and Study Centers:

Approximately 30 centers in the United States (U.S.)

Study Period:

Main Study:

Participation can last up to 30 weeks, which includes:

- Screening: 6 weeks
- On-Treatment: 16 weeks
- Safety Follow-up: 4 weeks
- Repeat Liver Biopsy: 4 weeks (in eligible subjects)

Cohort C:

Participation can last up to 26 weeks, which includes:

- Screening: 6 weeks
- On-Treatment: 16 weeks
- Safety Follow-up: 4 weeks

Phase of Development:

Phase 2a

Objectives:

Main Study:

The primary objective of this study is:

- To evaluate absolute change from baseline in hepatic fat fraction assessed by Magnetic Resonance Imaging - Proton Density Fat Fraction (MRI-PDFF) at Week 12

The secondary objectives of this study are:

- To evaluate percent change from baseline in hepatic fat fraction assessed by MRI-PDFF at Week 12
- To evaluate the responder: subjects who achieved a clinically meaningful relative reduction of at least 30% in liver fat content as measured by MRI-PDFF at Week 12
- To evaluate the responder based on NAFLD Activity Score (NAS) system: subjects who had a decrease of ≥ 2 points in NAS with at least a 1-point reduction in either lobular inflammation or hepatocellular ballooning and with no concurrent worsening of fibrosis stage
- To evaluate the change from baseline in ALT at Week 12
- To assess the safety and tolerability of EFX in subjects with NASH

Cohort C:

The primary objective of this study is:

- To assess the safety and tolerability of EFX in NASH subjects with compensated cirrhosis

The secondary objectives of this study are:

- To evaluate the change from baseline in liver stiffness as evaluated by FibroScan® at Week 16
- To evaluate the change from baseline in non-invasive plasma (serum) fibrosis biomarkers including liver fibrosis by ELF™ Test score and pro-C3 levels

Study Design:

This is a Phase 2a, randomized, double-blind, placebo-controlled study evaluating the safety and efficacy of EFX in subjects with NASH.

Main Study:

Subjects meeting the study's entry criteria will be randomly assigned in 1:1:1:1 ratio into 4 treatment groups as shown in the figure below:

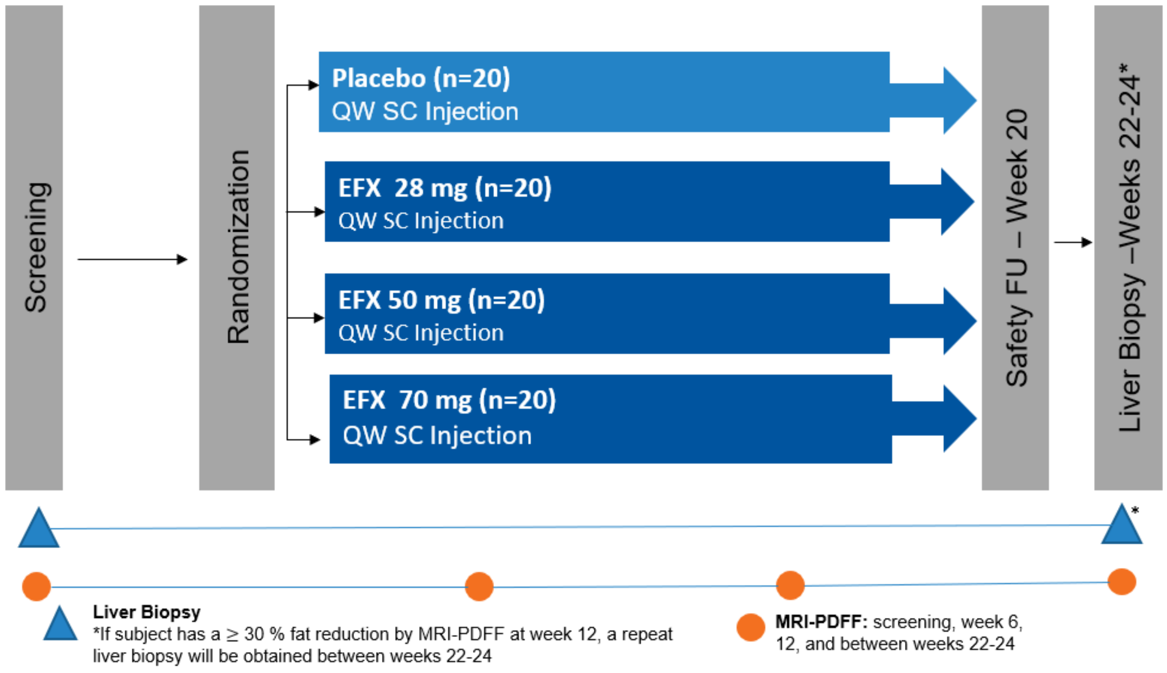

Cohort C:

Subjects meeting the study's entry criteria will be randomly assigned in 1:2 ratio into 2 treatment groups as shown in the figure below:

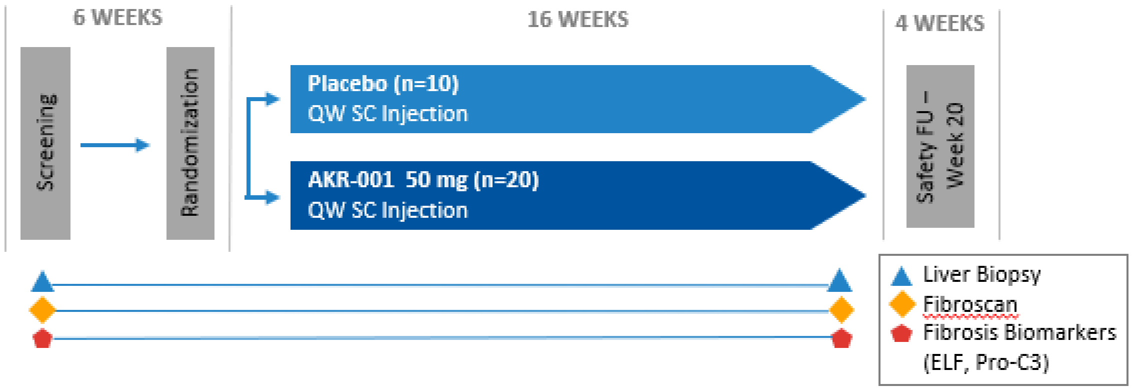

Following 16 weeks of treatment, subjects will be given the option to collect an end of treatment biopsy.

Randomization:

Main Study:

Randomization will be stratified by baseline hepatic fat fraction (< 15% vs ≥ 15%) and F1 fibrosis score.

Study drug will be administered subcutaneously once weekly (QW) for a total of 16 weeks.

Eligible subjects will be randomized to one of four treatment groups with a ratio 1:1:1:1:

Group A: Placebo

Group B: 28 mg EFX

Group C: 50 mg EFX

Group D: 70 mg EFX

After 16 weeks of treatment, a Safety Follow-up Visit will occur at Week 20. Subjects with ≥ 30% relative fat reduction on MRI-PDFF at Week 12 will be required to return between Weeks 22 - 24 for a repeat liver biopsy and MRI-PDFF.

Cohort C:

Study drug will be administered subcutaneously once weekly (QW) for a total of 16 weeks.

Eligible subjects will be randomized to one of two treatment groups with a ratio 1:2:

Group A: Placebo

Group B: 50 mg EFX

After 16 weeks of treatment, a Safety Follow-up Visit will occur at Week 20.

Number of Subjects Planned:

Total 110: Approximately 80 subjects in the Main Study and approximately 30 subjects in Cohort C.

Target Population:

Males and non-pregnant, non-lactating females between 18 – 80 years of age with biopsy proven F1 - F3 NASH. Limit F1 fibrosis to ≤ 20% of total subject population.

Diagnosis and Main Criteria for Inclusion:

Key Inclusion Criteria for the Main Study:

2. Body mass index (BMI) > 25 kg/m^2 (unless the subject has biopsy-proven NASH documented within the last 2 years).
3. Must have confirmation of ≥ 10% liver fat content on MRI-PDFF at screening.
4. Biopsy-proven NASH. Must have had a liver biopsy within 180 days of randomization with fibrosis stage 1 to 3 and a non-alcoholic fatty liver disease (NAFLD) activity score (NAS) of ≥ 4 with at least a score of 1 in each of the following NAS components:
    a. Steatosis (scored 0 to 3),
    b. Ballooning degeneration (scored 0 to 2), and
    c. Lobular inflammation (scored 0 to 3).
5. Must have no evidence of worsening of ALT and AST (within 50%) measurements at the screening (- 6 weeks) and pre-baseline (-2 weeks) visits.
6. Screening laboratory parameters, as determined by the central laboratory:
    a. Estimated glomerular filtration rate (eGFR) ≥ 60 mL/min, as calculated by the Cockcroft-Gault equation;
    b. HbA1c ≤ 9.5% (or serum fructosamine ≤ 381 μmol if HbA1c is unable to be resulted);
    c. Hemoglobin ≥ 11 g/dL;
    d. INR ≤ 1.3, unless due to therapeutic anticoagulation;
    e. Direct bilirubin ≤ 0.3 mg/dL;
    f. Total bilirubin ≤ 1.3 x upper limit of normal (ULN), unless due to an alternate etiology such as Gilbert's syndrome or hemolytic anemia;
    g. Creatinine kinase < 3 x ULN;
    h. Platelet count ≥ 150,000/μL;
    i. Serum triglyceride level ≤ 500 mg/dL;
    j. ALT < 5 x ULN;
    k. AST < 5 x ULN;
    l. AST > 20u/L (only required for subjects without a historical biopsy in the last 2 years confirming fibrosis 1-3 and a NAS ≥ 4);
    m. ALP < 2 x ULN.
7. FibroScan® measurement > 7.0 kPa.

Key Inclusion Criteria for Cohort C:

2. Compensated cirrhosis (Fibrosis Stage 4) due to NASH documented by a local pathologist within 24 months of Screening with no evidence of competing etiology, and at least one co-existing or historic metabolic comorbidity. If a historical biopsy is not available within 24 months of Screening, the subject may undergo a liver biopsy to be assessed by the local or central reader and confirm to have compensated cirrhosis (Fibrosis Stage 4).
3. FibroScan® measurement > 13.1 kPa.
4. Enhanced Liver Fibrosis (ELF) score of > 9.8.
5. Must have no evidence of worsening of ALT and AST (within 50%) measurements at the screening (-6 weeks) and pre-baseline (-2 weeks) visits.
    a. A single retest of the pre-baseline labs is permitted only if there is reason to believe the initial value was erroneous due to a sample processing error or due to an extenuating circumstance. An average of the screening and pre-baseline value will be used and compared to the retest value. The retest value must have no evidence of worsening within 50% of the average of the screening and pre-baseline value.

6. Screening laboratory parameters, as determined by the central laboratory:
    a. Estimated glomerular filtration rate (eGFR) ≥ 60 mL/min, as calculated by the Cockcroft-Gault equation;
    b. HbA1c ≤ 9.5% (or serum fructosamine ≤ 381 μmol if HbA1c is unable to be resulted);
    c. Hemoglobin ≥ 11 g/dL;
    d. INR ≤ 1.3, unless due to therapeutic anticoagulation;
    e. Direct bilirubin ≤ 0.3 mg/dL;
    f. Total bilirubin ≤ 1.3 x upper limit of normal (ULN), unless due to an alternate etiology such as Gilbert's syndrome or hemolytic anemia;
    g. Creatinine kinase < 3 x ULN;
    h. Platelet count ≥ 125,000/μL;
    i. Serum triglyceride level ≤ 500 mg/dL;
    j. ALT ≤ 5 x ULN;
    k. AST < 5 x ULN;
    l. ALP < 2 x ULN;
    m. Albumin ≥ 3.5 g/dL.

<u>Key Exclusion Criteria for the Main Study</u>:

1. Weight gain or loss > 5% in the 3 months prior to randomization or > 10% in the 6 months prior to screening.
2. Type 1 and insulin-dependent Type 2 diabetes.
3. Presence of cirrhosis on liver biopsy (stage 4 fibrosis).
4. Poorly controlled hypertension (blood pressure [BP] > 160/100).
5. Prior history of decompensated liver disease including ascites, hepatic encephalopathy (HE), or variceal bleeding.
6. Chronic hepatitis B virus (HBV) infection (hepatitis B surface antigen [HBsAg] positive).
7. Chronic hepatitis C virus (HCV) infection (HCV antibody [Ab] and HCV ribonucleic acid [RNA] positive). Subjects cured of HCV infection less than 2 years prior (based on date of RNA polymerase chain reaction [PCR] negative confirmation following conclusion of treatment) to the screening visit are not eligible.
8. Prior or planned (during the study period) bariatric surgery (e.g., gastroplasty, roux-en-Y gastric bypass), surgery reversal or removal of intragastric balloon > 2 years prior to enrollment would be eligible.
9. Other causes of liver disease based on medical history and/or centralized review of liver histology, including but not limited to: alcoholic liver disease, autoimmune disorders (e.g., primary biliary cholangitis [PBC], primary sclerosing cholangitis [PSC], autoimmune hepatitis), drug-induced hepatotoxicity, Wilson disease, clinically significant iron overload, or alpha-1-antitryspin deficiency requiring treatment.
10. Subjects with osteoporosis, defined as a T-score of -2.5 or lower at screening.
11. History of liver transplantation.
12. Current or prior history of hepatocellular carcinoma (HCC).
13. Alcohol intake above an average limit of 2 drinks per day for women and 3 drinks per day for men assessed by alcohol use disorders identification test (AUDIT-C) questionnaire.
14. Human immunodeficiency virus (HIV) infection.
15. Unstable cardiovascular disease in the 6 months prior to screening.
16. Life expectancy less than 2 years.

<u>Key Exclusion Criteria for Cohort C</u>:

1. Weight gain or loss > 5% in the 3 months prior to randomization or > 10% in the 6 months prior to screening.
2. Type 1 and insulin-dependent Type 2 diabetes.
3. Prior history of or current decompensated liver disease including ascites, hepatic encephalopathy (HE), or variceal bleeding.

4. Child-Pugh Score > 6 (Class B or C), as determined at screening, unless due to therapeutic anti-coagulation.
5. MELD score > 12, as determined at screening, unless due to therapeutic anti-coagulation.
6. Poorly controlled hypertension (blood pressure [BP] > 160/100).
7. Chronic hepatitis B virus (HBV) infection (hepatitis B surface antigen [HBsAg] positive).
8. Chronic hepatitis C virus (HCV) infection (HCV antibody [Ab] and HCV ribonucleic acid [RNA] positive). Subjects cured of HCV infection less than 2 years prior (based on date of RNA polymerase chain reaction [PCR] negative confirmation following conclusion of treatment) to the screening visit are not eligible.
9. Prior or planned (during the study period) bariatric surgery (e.g., gastroplasty, roux-en-Y gastric bypass), surgery reversal or removal of intragastric balloon > 2 years prior to enrollment would be eligible.
10. Other causes of liver disease based on medical history and/or centralized review of liver histology, including but not limited to: alcoholic liver disease, autoimmune disorders (e.g., primary biliary cholangitis [PBC], primary sclerosing cholangitis [PSC], autoimmune hepatitis), drug-induced hepatotoxicity, Wilson disease, clinically significant iron overload, or alpha-1-antitryspin deficiency requiring treatment.
11. Subjects with osteoporosis, defined as a T-score of -2.5 or lower at screening.
12. History of liver transplantation.
13. Current or prior history of hepatocellular carcinoma (HCC).
14. Alcohol intake above an average limit of 2 drinks per day for women and 3 drinks per day for men assessed by alcohol use disorders identification test (AUDIT-C) questionnaire.
15. Human immunodeficiency virus (HIV) infection.
16. Unstable cardiovascular disease in the 6 months prior to screening.
17. Life expectancy less than 2 years.

Investigational Product, Dosage, and Mode of Administration:

EFX is an IgG1Fc-fibroblast growth factor 21 (FGF21) fusion protein.

Main Study:

EFX or Placebo is administered subcutaneously as follows:

- Placebo QW for 16 weeks (n = 20)
- 28 mg EFX QW for 16 weeks (n = 20)
- 50 mg EFX QW for 16 weeks (n = 20)
- 70 mg EFX QW for 16 weeks (n = 20)

Cohort C:

EFX or Placebo is administered subcutaneously as follows:

- Placebo QW for 16 weeks (n = 10)
- 50 mg EFX QW for 16 weeks (n = 20)

Criteria for Evaluation:

Safety:

The safety of EFX will be assessed during the study through the reporting of adverse events (AEs), clinical laboratory tests, electrocardiogram (ECG), vital sign assessments, body weight, anti-drug antibody (ADA) and neutralizing antibody (NAB) assessments, and concomitant medication usage.

An external Data Monitoring Committee (DMC) that consists of two hepatologists, one cardiologist, and a statistician will review the progress of the study. The DMC will convene after 20 subjects (approximately 5 per treatment group in the Main Study) have completed the Week 4 assessments. The DMC will receive all reports of serious adverse events (SAEs) and convene as needed to monitor for safety.

Pharmacokinetics:

Blood for pharmacokinetics (PK) will be collected at specified timepoints after dosing as outlined in the Schedule of Assessments (Appendix A and Appendix B) and will be used to estimate steady state PK parameters (i.e., T_{max}, C_{max}, C_{trough}, AUC_{0-t}, $t_{½}$, CL/F, Vz/F) following subcutaneous (SC) administration of EFX.

Efficacy:

Main Study:

The efficacy will be assessed via the absolute change from baseline in hepatic fat fraction measured by MRI-PDFF at Week 12.

Cohort C:

The efficacy will be assessed via the change from baseline in liver stiffness as evaluated by FibroScan® at Week 16 and non-invasive plasma (serum) fibrosis biomarkers including Liver Fibrosis by ELF™ Test score and pro-C3 levels.

Sample Size Calculation:

Main Study:

With the following assumptions, the power for N = 20 subjects with active treatment is approximately 91% to detect:

- a true, baseline-adjusted, mean decrease from baseline in MRI-PDFF-estimated hepatic fat content of 5% (absolute) compared to placebo,
- with a one-sided t-test for decrease from placebo,
- at a 5% significance level,
- 20 subjects receiving placebo, and
- a standard deviation estimated as 5.2% (Sanyal et al. 2019).

Cohort C:

Since the primary objective for Cohort C is to assess the safety and tolerability of EFX in NASH subjects with compensated cirrhosis, the sample size of Cohort C is determined based on clinical considerations, and the treatment comparisons of efficacy measurements are not powered.

Statistical Methods:

Efficacy Analysis:

Primary Efficacy Analysis (Main Study Only):

The primary efficacy variable will be the absolute change from baseline hepatic fat fraction measured by MRI-PDFF at Week 12. Summary statistics (number of subjects, mean, standard deviation, median, minimum, and maximum) at all visits, and change from baseline will be provided. The primary efficacy variable will be analyzed using an analysis of covariance (ANCOVA) model with treatment group and F1 fibrosis score as factors and baseline hepatic fat fraction (absolute %) measured by MRI-PDFF as a covariate. For any subjects in the full analysis set (FAS) with a missing primary efficacy value, the primary efficacy variable will be imputed with multiple imputation method. The specification of the multiple imputation method will be provided in the SAP. Fixed hypothesis testing sequence will be used to control the multiplicity for each active dose to be compared with placebo.

Secondary Efficacy Analysis:

Main Study:

For the continuous secondary efficacy variables related to hepatic fat fraction, like percent change from baseline in fat fraction assessed by MRI-PDFF at Week

	12, the same ANCOVA model used for primary efficacy variable will be used. For the other continuous secondary efficacy variables, the ANCOVA model with treatment group, baseline hepatic fat fraction (< 15% vs ≥ 15%), and F1 fibrosis score as factors will be used. Normality will be tested for the model residuals. For certain efficacy variables, logarithmic transformation may be performed prior to fitting the ANCOVA model.<br>For the responder(s) based on MRI-PDFF, logistic regression will be used for the analysis. The analyses will include the treatment group and F1 fibrosis score as factors and baseline hepatic fat fraction assessed by MRI-PDFF as a covariate. The missing value will be imputed as a non-responder.<br>For the responder(s) based on the NAS system, Fisher's exact test will be used for the analyses. Odds ratios, 95% confidence intervals and p-values will be provided. The missing value will be imputed as a non-responder.<br>**Cohort C:**<br>Summary statistics (number of subjects, mean, standard deviation, median, minimum, and maximum) at all visits and change from baseline will be provided. The efficacy variables will be analyzed with an ANCOVA model with treatment group as a factor and baseline value as a covariate. The effect of Type 2 diabetes status will be explored. Normality will be tested for the model residuals.<br>**Exploratory Efficacy Analyses:**<br>The same analyses used for the secondary efficacy variables will be used for the exploratory efficacy variables.
**Pharmacokinetic Analysis:**	Plasma concentrations of EFX will be listed and summarized.
Detailed description of data analysis and statistical methods to be used will be outlined separately in a Statistical Analysis Plan (SAP).	

This study will be conducted in accordance with the guidelines of Good Clinical Practice (GCP) including archiving of essential documents.

TABLE OF CONTENTS

STUDY ACKNOWLEDGEMENT SIGNATURE PAGE......2
SYNOPSIS......3
TABLE OF CONTENTS......11
LIST OF TABLES......15
LIST OF FIGURES......15
LIST OF APPENDICES......16
LIST OF ABBREVIATIONS AND DEFINITIONS OF TERMS......16
1. BACKGROUND AND RATIONALE......21
1.1. Background......21
1.1.1. FGF21......21
1.2. EFX Background......22
1.3. Nonclinical Findings......23
1.3.1. Pharmacology......23
1.3.2. Pharmacokinetics......24
1.3.3. Toxicology......24
1.3.3.1. Repeated-dose Toxicology Studies......24
1.3.3.2. Genotoxicity (Mutagenicity)......26
1.3.3.3. Carcinogenicity......27
1.3.3.4. Developmental and Reproductive Toxicity......27
1.3.3.5. Ongoing/Planned Toxicology Studies of EFX......27
1.3.3.6. Margin of Safety for Clinical Dose of EFX......27
1.4. Clinical Trials of EFX......28
1.4.1. Study 20100015 - Phase 1a Single Ascending Dose Study in Diabetic Patients......28
1.4.1.1. Subject Disposition......29
1.4.1.2. Pharmacokinetic Results......29
1.4.1.3. Pharmacodynamic Results......29
1.4.1.4. Safety Results......29
1.4.1.5. Conclusion......30
1.4.2. Study 20100018 - Phase 1b Multiple Ascending Dose Study in Diabetic Patients......31
1.4.2.1. Subject Disposition......31
1.4.2.2. Pharmacokinetic Results......31
1.4.2.3. Pharmacodynamic Results......32
1.4.2.4. Safety Results Describing the Combined Q2W and QW Datasets......33

1.4.2.5. Conclusion 34

1.5. Rationale for this Study 35
1.6. Rationale for Selection of Dose 37
1.7. Compliance 38

2. STUDY OBJECTIVES 39

2.1. Primary Objectives....39
2.2. Secondary Objectives....39
2.3. Exploratory Objectives 39

3. INVESTIGATIONAL PLAN 41

3.1. Overall Study Design and Plan 41
3.2. Study Duration and Number of Centers....42
3.3. End of Study 42
3.4. Early Termination 42
3.5. Post Study Care....43

4. STUDY POPULATION SELECTION....44

4.1. Study Population....44
4.2. Inclusion Criteria 44
4.3. Exclusion Criteria 46

5. INVESTIGATIONAL MEDICINAL PRODUCTS (IMP)....50

5.1. Randomization, Blinding and Treatment Codes....50

5.1.1. Procedures for Breaking Treatment Codes....50

5.2. Investigational Product(s) 50

5.2.1. Storage and Handling....51
5.2.2. Dosage and Administration....52

5.3. Prior and Concomitant Medications 52
5.4. Investigational Medicinal Product Accountability 54

5.4.1. Investigational Medicinal Product Return or Disposal....54

6. STUDY PROCEDURES....55

6.1. Screening and Informed Consent....55
6.2. Demographics and Medical History 55
6.3. Physical Examination....55

6.4. Vital Signs, Weight, Hip and Waist Circumference 56
6.5. 12-Lead Electrocardiography....56
6.6. FibroScan® 57
6.7. Liver Biopsy....57
6.8. MRI-PDFF 58
6.9. DXA Scan 58
6.10. Health Related Quality of Life Questionnaires....59

6.10.1. Short Form 36 Health Survey (SF-36)....59
6.10.2. Chronic Liver Disease Questionnaire-Nonalcoholic Fatty Liver Disease (CLDQ-NAFLD) 59
6.10.3. Work Productivity and Activity Impairment (WPAI) 59

6.11. Alcohol Use Disorders Identification Test (AUDIT-C) 59
6.12. Lifestyle Modification 59
6.13. Clinical Laboratory Tests....60

6.13.1. Laboratory Parameters 60

6.14. Sample Collection, Storage, and Shipping 62
6.15. Pharmacokinetic Assessments 62
6.16. Anti-Drug Antibody and Neutralizing Antibody Sampling....62
6.17. Blood Storage....62
6.18. Biomarkers....62
6.19. Criteria for Discontinuation of Study Drug 63
6.20. Interruption of Study Drug....64
6.21. Adverse Events 64

6.21.1. Severity 65
6.21.2. Relationship 65
6.21.3. Overdose 66
6.21.4. Serious Adverse Events 66

6.21.4.1. Definition 66

6.21.5. Reporting Adverse Events 66

6.21.5.1. Reporting Procedures for Non-Serious Adverse Events....66
6.21.5.2. Reporting Procedures Serious Adverse Events....67

6.22. Adverse Events of Special Interest 69
6.23. Clinical Laboratory Abnormalities and Other Abnormal Assessments as Adverse Events or Serious Events....69
6.24. Observation for Drug Induced Liver Injury (DILI) 71

6.24.1. Subjects with Normal Liver Transaminases and Bilirubin at Baseline 71
6.24.2. Subjects with Elevations in Liver Transaminases or Bilirubin at Baseline....71

6.24.3. Close Observation for Suspected Drug-Induced Liver Injury 72
6.25. Removal of Subjects from the Study 73
7. PLANNED STATISTICAL METHODS 74
7.1. Endpoints 74
7.1.1. Efficacy Endpoints 74
7.1.1.1. Primary Endpoint 74
7.1.1.2. Secondary Efficacy Endpoints 74
7.1.1.3. Exploratory Efficacy Endpoints 74
7.1.2. Safety Endpoints 75
7.2. Analysis Conventions 76
7.3. Analysis Sets 76
7.3.1. Efficacy 76
7.3.2. Safety 76
7.3.2.1. Pharmacokinetics 76
7.3.2.2. Biomarkers 76
7.4. Data Handling Conventions 76
7.5. Demographic Data and Baseline Characteristics 77
7.6. Efficacy Analysis 77
7.6.1. Primary Efficacy Analysis 77
7.6.2. Secondary Efficacy Analysis 77
7.6.3. Exploratory Efficacy Analysis 78
7.7. Safety Analysis 78
7.7.1. Extent of Exposure 78
7.7.2. Adverse Events 78
7.7.3. Laboratory Evaluations 79
7.8. Pharmacokinetic Analysis 79
7.9. Biomarker Analysis 79
7.10. Sample Size 80
7.11. Data Monitoring Committee 80
7.11.1. Stopping Rules 81
7.12. Adjudication Committee 81

8. ADMINISTRATIVE CONSIDERATIONS 82
8.1. Investigator Responsibilities 82
8.1.1. Good Clinical Practice 82
8.1.2. Institutional Review Board (IRB)/Independent Ethics Committee (IEC)/ Ethics Committee (EC) Review and Approval 82
8.1.3. Informed Consent 82
8.1.4. Confidentiality 83
8.1.5. Study Files and Retention of Records 83
8.1.6. Electronic Case Report Forms 84
8.1.7. Inspections 85
8.1.8. Protocol Compliance 85
8.2. Sponsor Responsibilities 85
8.2.1. Protocol Modifications 85
8.2.2. Study Report and Publications 85
8.3. Joint Investigator/Sponsor Responsibilities 86
8.3.1. Payment Reporting 86
8.3.2. Access to Information for Auditing or Inspections 86
8.3.3. Study Discontinuation 86
9. REFERENCE LIST 87
10. APPENDICES 90

LIST OF TABLES

Table 1. Systemic Exposures in Monkey, Rat, and Humans at the NOAEL and Margin of Safety in Humans 28
Table 2. Treatment-Related Adverse Events and Withdrawals 34
Table 3. Clinical Laboratory Assessments 61

LIST OF FIGURES

Figure 1. EFX Schematic Structure with Disulfide Bonds and Chains 23
Figure 2. Study Schema – Main Study 41
Figure 3. Study Schema – Cohort C 42

LIST OF APPENDICES

Appendix A. Schedule of Assessments- Main Study 90
Appendix B. Schedule of Assessments- Cohort C 93
Appendix C. Pregnancy Precautions, Definition for Female of Childbearing Potential, and Contraceptive Requirements 96

LIST OF ABBREVIATIONS AND DEFINITIONS OF TERMS

ADA	anti-drug antibodies
AE	adverse event
AESI	adverse event of special interest
ALP	alkaline phosphatase
ALT	alanine aminotransferase (SGPT)
ANC	absolute neutrophil count
ANCOVA	analysis of covariance
aPTT	activated partial thromboplastin time
AST	aspartate aminotransferase (SGOT)
AUC	area under the time-concentration curve
AUDIT-C	alcohol use disorders identification test
BMI	body mass index
BSAP	bone specific alkaline phosphatase
BUN	blood urea nitrogen
CAP	controlled attenuation parameter
CFR	Code of Federal Regulations
CI	confidence interval
C_{max}	maximum plasma drug concentration
C_{min}	minimum plasma drug concentration
CP	Child-Pugh
CRF	case report form
cT1	Corrected T1
CTCAE	Common Terminology Criteria for Adverse Events

CV	coefficient of variation
DILI	drug-induced liver injury
DNA	deoxyribonucleic acid
DXA	Dual Energy X-ray Absorptiometry
ECG	electrocardiogram
eCRF	electronic Case Report Form
EFX	efruxifermin
eGFR	estimated Glomerular Filtration Rate
ELF™	Enhanced Liver Fibrosis Panel
FAS	full analysis set
FDA	Food and Drug Administration
FGF	fibroblast growth factor
FGFR	fibroblast growth factor receptor
FVB	Friend Virus B
GCP	Good Clinical Practice
GGT	gamma glutamyl transferase
GLP	Good Laboratory Practice
HbA1c	hemoglobin A1c
HBcAb	total hepatitis B core antibody
HBsAg	hepatitis B surface antigen
HBV	hepatitis B virus
HCC	hepatocellular carcinoma
Hct	hematocrit
HCV	hepatitis C virus
HDL	high-density lipoprotein cholesterol
Hgb	hemoglobin
HIPAA	Health Information Portability and Accountability Act
HITECH	Health Information Technology for Economic and Clinical Health
HIV	human immunodeficiency virus

HLGT	high-level group term
HLT	high-level term
HOMA	homeostasis model assessment of insulin resistance, (HOMA-IR) and beta cell function (HOMA-B)
HRQoL	health-related quality of life
ICF	informed consent form
ICH	International Council for Harmonisation
IEC	Independent Ethics Committee
IgG1	human immunoglobulin G1
IMP	investigational medicinal product
IND	Investigational New Drug
INR	International Normalized Ratio
IP	investigational product
IRB	Institutional Review Board
IRT	interactive response technology
ITT	intent-to-treat
IV	intravenous
KO	knock-out
LDH	lactate dehydrogenase
LDL	low-density lipoprotein cholesterol
LLT	low-level term
LOCF	last observation carried forward
mAb	monoclonal antibody
MedDRA	Medical Dictionary for Regulatory Activities
MELD	Model for End-Stage Liver Disease
MRE	magnetic resonance elastography
MRI	magnetic resonance imaging
MRI-PDFF	magnetic resonance imaging – proton density fat fraction
NAFLD	non-alcoholic fatty liver disease
NAS	non-alcoholic fatty liver disease activity score

NASH	nonalcoholic steatohepatitis
NOAEL	no-observed-adverse-effect-level
PBC	primary biliary cholangitis
PD	pharmacodynamics
PI	principal investigator
PK	pharmacokinetics
PSC	primary sclerosing cholangitis
PT	preferred term
PT	prothrombin time
Q2W	every 2 weeks
QW	every week
RBC	red blood cell (count)
RNA	ribonucleic acid
SC	subcutaneous
SAE	serious adverse event
SAP	Statistical Analysis Plan
SD	standard deviation
SE	standard error
SGOT	serum glutamate oxaloacetate transaminase (AST)
SGPT	serum glutamate pyruvate transaminase (ALT)
SOC	system organ class
SUSAR	suspected unexpected serious adverse reaction
$T_{1/2}$	time to half plasma concentration (half-life)
TBL	Total bilirubin
TEAEs	treatment emergent adverse events
TESAEs	treatment-emergent serious AEs
TK	toxicokinetics
T_{max}	time to maximum plasma concentration
TSH	thyroid stimulating hormone

ULN	upper-limit of normal
U.S.	United States
WBC	white blood cell (count)

1. BACKGROUND AND RATIONALE

1.1. Background

Nonalcoholic steatohepatitis (NASH) is the most severe form of non-alcoholic fatty liver disease (NAFLD) and is characterized by the presence of an abnormal accumulation of fat in the liver, which in some individuals can progress to liver cell injury (hepatocellular ballooning), inflammation, and fibrosis. Lipotoxicity and oxidative stress are considered to be the drivers of disease progression, or the underlying cause of the disease. As NASH progresses, it can result in excessive deposition of extracellular collagen, or scarring of the liver (fibrosis) ultimately leading to liver cirrhosis. Later stages of NASH are associated with substantially increased risk of hepatocellular carcinoma and major adverse cardiac events (Ekstedt et al. 2015), underlining the healthcare burden presented by NASH in the absence of approved therapies.

NASH is closely related to the epidemic of obesity and diabetes and is often viewed as the liver manifestation of the Metabolic Syndrome. It is heavily influenced by lifestyle (e.g., chronically excessive calorie intake, sedentary activity) and is distinct from other fatty liver diseases caused by viral infection, alcohol abuse, or medication side effects (The Nash Education Program 2019). NASH can potentially progress to advanced liver disease, cirrhosis, and hepatocellular carcinoma (The Nash Education Program 2019). The current prevalence of NASH in the United States (U.S.) population is estimated to be around 3 – 4%. Approximately 1 in 5 of the near 80 million patients with NAFLD in the U.S. (Ruhl & Everhart 2015) are projected to develop NASH (Younossi et al. 2016). This unfurling epidemic of liver disease warrants a major public health effort to control the burden of disease, in particular its many complications/ co-morbid conditions.

1.1.1. FGF21

Fibroblast growth factor 21 (FGF21) is a member of the endocrine FGF sub-family. It depends on a high affinity interaction with β-Klotho to enable formation of a co-receptor complex with fibroblast growth factor receptor (FGFR) to initiate intracellular signaling. FGF21 is a secreted polypeptide highly expressed in tissues relevant to metabolic function, including the liver, adipose tissue, and pancreas (Fon Tacer et al. 2010, Nishimura et al. 2000). Studies have shown that FGF21 is a metabolic regulator of energy homeostasis, maintaining appropriate balance across glucose-lipid-protein utilization, and enhancing insulin sensitivity in the fed state (Kim et al. 2017, Gaich et al. 2013, Li et al. 2013, Talukdar et al. 2016). FGF21 directly modulates whole-body lipid metabolism to reduce hepatic lipid accumulation. FGF21 has been shown to reverse hepatic steatosis and to prevent diet-induced obesity in both rodents and nonhuman primates (Lin et al. 2013, Camporez et al. 2013, Mu et al. 2012, Xu et al. 2009, Coskun et al. 2008, Wu et al. 2017, Talukdar et al. 2016). Hence, FGF21 is a target for the development of novel biological drugs for metabolic diseases (Gimeno & Moller 2014, Chen et al. 2017). FGF21 forms a ligand co-receptor complex by anchoring to β-Klotho then interacts with one of three members of the FGFR family (specifically FGFR1c, 2c and 3c) to induce dimerization and autophosphorylation of FGFR. Activated FGFR initiates specific intracellular signaling cascades leading to the expression of

FGF21 autocrine biological functions (Li et al. 2013). Serum FGF21 levels are elevated in human subjects with diabetes, obesity, and NASH. This has been interpreted as evidence of FGF21 resistance (Li et al. 2013, Wu et al. 2017).

In NASH, FGF21 appears to address both the underlying disease driver, i.e. excessive flux of calories and lipid into the liver, and the ensuing downstream sequelae of hepatocyte stress leading to hepatic inflammation and fibrosis. FGF21 directly modulates whole body lipid metabolism to reduce hepatic lipid accumulation and has been shown to reverse hepatic steatosis and to prevent diet-induced obesity in both rodents and nonhuman primates (Lin et al. 2013, Camporez et al. 2013, Mu et al. 2012, Xu et al. 2009, Coskun et al. 2008, Wu et al. 2017, Talukdar et al. 2016).

Various in vitro and in vivo findings suggest potential beneficial effects of FGF21 agonism in NASH, including improvements in liver fat, inflammation, and fibrosis (Xu et al. 2009, Lee et al. 2016, Tanaka et al. 2015). Administration of native FGF21or long-acting FGF21 analogues prevented an increase in liver fat and suppressed inflammation and fibrosis, as assessed by histology, in rodent models of liver disease (Tanaka et al. 2015, Fisher et al. 2014, Bao et al. 2018). The models used a range of pathological insults, including excessive intake of fat and fructose, alcohol, diet deficient in methionine and choline, or chemical toxins (e.g., carbon tetrachloride, dimethyl nitrosamine; [Xu et al. 2016]). Increasing FGF21 tone was effective in all the models, whereas loss of FGF21 function exacerbated development of liver pathology.

Genetic and pharmacological studies provide further evidence that FGR21 agonism could have potentially beneficial effects on NASH. FGF21 knockout (KO) mice are viable, fertile, and show no strong metabolic phenotypes when fed a normal chow diet. However, they become insulin resistant with hepatosteatosis and have significantly elevated plasma glucose and triglyceride levels when challenged with high-fat or ketogenic diets. In contrast, transgenic mice over-expressing FGF21 are lean and resistant to diet induced or age-associated obesity and insulin resistance.

1.2. EFX Background

EFX is a 92.0 kD *E. coli*-expressed human IgG_1-Fc- Fc-FGF21 fusion protein—a long-acting FGF21 analogue. Each molecule contains one dimeric Fc portion and 2 modified FGF21 proteins. EFX has 8 disulfide bonds, 6 intrachain, and 2 interchain as depicted in Figure 1. Two of the intrachain disulfide bonds are in the FGF21 protein between Cys318 and Cys336, one for each monomer unit. Additionally, 3 mutation sites, L98R (residue 341 in Fc-fusion), P171G (414 in Fc-fusion), and A180E (423 in Fc-fusion) have been introduced into the wild-type human FGF21 sequence to decrease its susceptibility to in vivo proteolytic degradation and reduce aggregation while maintaining balanced FGF21 agonism across FGFR1c/2c/3c with selectivity over FGFR4. A schematic of the structure is shown in Figure 1.

Figure 1. EFX Schematic Structure with Disulfide Bonds and Chains

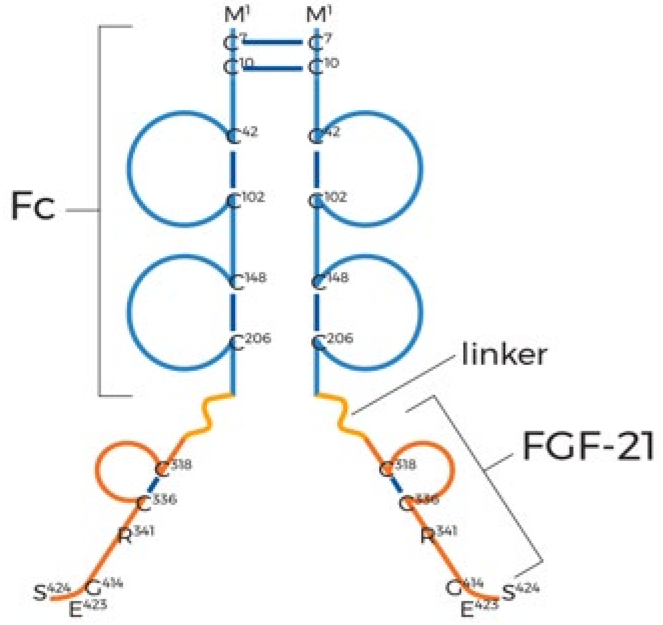

1.3. Nonclinical Findings

The intended clinical route of administration for EFX is via subcutaneous (SC) injection. EFX was studied in 4-week toxicology studies in rat and cynomolgus monkeys to support the original diabetes indication. Subsequently, EFX was examined in 16-week toxicology studies in rat and cynomolgus monkeys to support this planned Phase 2 clinical study in NASH patients. The results from the 16-week studies as well as previously conducted nonclinical studies are described in the Investigator's Brochure (IB).

1.3.1. Pharmacology

In vitro characterization demonstrated that EFX bound to human, cynomolgus monkey, and mouse β-Klotho with EC_{50} values of 11 nM, 10 nM, and 0.8 nM, respectively. In cells transiently co-expressing β-Klotho and individual FGFR, EFX selectively activated c-isoforms of FGFR, FGFR1c, 2c and 3c, with balanced potency. The potency of EFX determined by measuring its ability to activate the β-Klotho dependent MAPK pathway was 1.4 nM in human β-Klotho recombinant HEK 293 cells, and 0.44 nM in human primary adipocytes. EFX displayed cross species reactivity with comparable in vitro potency to activate receptor complex (FGFR1c/β-Klotho) from human, cynomolgus monkey, rat, and mice. In vivo, EFX is pharmacologically active in rodents and monkeys. Administration of EFX to obese and insulin resistant mouse and monkey models improved the metabolic state of these animals resulting in reduced plasma glucose, insulin, and triglyceride levels; improved lipoprotein profile; enhanced glucose tolerance; and significant weight loss.

Evaluation of cardiovascular, neurobehavioral, and respiratory function were incorporated into the 4-week repeated dose toxicity study with cynomolgus monkey treated with weekly doses of EFX of up to 100 mg/kg. No EFX-related effects on electrocardiograms (ECG) or pulse oximetry were observed, and no neurological abnormalities were observed at any dose in the treated monkeys.

1.3.2. Pharmacokinetics

The toxicokinetics (TK) of EFX was characterized in repeat-dose (weekly) SC toxicology studies conducted in Sprague Dawley rats and cynomolgus monkeys. EFX systemic exposure was approximately dose-proportional over the dose range of 1 - 100 mg/kg in rats and monkeys with no gender differences. Accumulation was less than 2-fold after once weekly doses of EFX for 16 weeks in rats and monkeys. The terminal half-life ($t_{1/2,z}$) was 3-4 days in rats and monkeys.

1.3.3. Toxicology

1.3.3.1. Repeated-dose Toxicology Studies

The toxicology studies conducted to support this Phase 2 clinical trial consisted of 16-week dosing in rats and cynomolgus monkeys. These studies were conducted in compliance with Good Laboratory Practice (GLP) and consistent with ICH M3(2) and ICH S6(R1). The summary findings are described below with further details provided in the current IB.

1.3.3.1.1. 4-Week Toxicity Studies Supporting Phase I

In a 4-week SC injection study (Study 110993), EFX was administered at doses of 0, 1, 10, or 100 mg/kg to Sprague-Dawley rats. In this study, EFX-related pharmacologic effects observed included decreased body weight gain and mean body weight. Based on these results, the no-observed-adverse-effect-level (NOAEL) for this study was determined to be the highest dose evaluated: 100 mg/kg.

In the 4-week subcutaneous injection study (Study 110988), EFX was administered at doses of 0, 1, 10 or 100 mg/kg to cynomolgus monkeys. Administration of EFX at 1, 10, or 100 mg/kg SC was well tolerated. EFX related pharmacologic effects included decreased body weights, decreased insulin, and decreased glucose. Other EFX-related effects were limited to minimal increases of alanine aminotransferase (ALT) and aspartate aminotransferase (AST), decreased thymus weights and lymphoid cellularity in females at 100 mg/kg. Based on these results, the NOAEL for this study was determined to be 100 mg/kg SC.

1.3.3.1.2. A 16-Week Subcutaneous Injection Toxicity Study of EFX in Sprague-Dawley Rats (Study 6700591)

In this study, Sprague Dawley rats were treated weekly with EFX by SC injection at doses of 0, 10, 30, or 100 mg/kg.

EFX related pharmacologic effects included decreased body weights, changes in food consumption, decreased triglyceride, protein, and albumin. Other EFX-related effects were limited to minimal increases of ALT without corroborative changes in microscopic appearance of liver, and decreased adipocyte cellularity in sternal and femoral bone marrow without discernible changes in peripheral blood cell populations. Additionally, decreases in the bone resorption marker CTX were observed, suggesting a lower bone turnover. However, no treatment-related changes in the bone formation marker PINP were observed. These changes are considered to be associated with under-nutrition/negative caloric balance arising from the pharmacological effect of EFX.

During the study, a dose-related increase in systemic exposure was evident across doses with serum concentrations of EFX at Day 1 and Day 57 being similar. At the end of the study (Day 113), a decrease in EFX concentrations was observed for all doses. The lower exposure based on C_{max} values was most notable in the 10 and 100 mg/kg groups, with exposure at Day 113 up to 80% lower compared to Day 57 levels. The AUC values showed a similar decline. The presence of anti-EFX antibodies was confirmed for 5 of the 26 animals (17.3%) in the 10 mg/kg group, 1 of the 26 animals (3.8%) in the 30 mg/kg group and 10 of the 26 animals (36.5%) in the 100 mg/kg group. However, the presence of ADAs could not be correlated with the lower exposure for all animals.

Based on these results, the no-observed-adverse-effect level (NOAEL) was considered to be 100 mg/kg for males and females. The corresponding C_{max} and AUC_{0-24hr} values at Day 113 were 17.5 µg/mL and 678 hr*µg/mL, respectively, for males and 120.0 µg/mL and 7650 hr*µg/mL, respectively, for females. Due to the variation in the Day 113 systemic exposures observed for male and female rats, the pharmacokinetic exposure values at Day 1 and Day 57 were used to calculate the safety margins (Section 1.3.3.6) and appear more representative of the exposure over the course of the study. The corresponding C_{max} and AUC_{0-24hr} values at Day 57 were 110 µg/mL and 7440 hr*µg/mL, respectively, for males and 311 µg/mL and 16,900 hr*µg/mL, respectively, for females.

1.3.3.1.3. A 16-Week Subcutaneous Injection Toxicity Study of EFX in Cynomolgus Monkeys (Study 6700592)

Cynomolgus monkeys (n = 3/sex/group) were treated with EFX weekly by SC injection at doses of 0, 10, 30, or 100 mg/kg. No EFX-related effects on ophthalmology, electrocardiology including heart rate, coagulation or urinalysis evaluation were observed during the study.

EFX related effects included decreased body weights, minimal increases of ALT and AST, decreased thymus weights associated with decreased lymphoid cellularity, increased cortisol, decreased red blood cell, hemoglobin and hematocrit levels.

At doses of ≥10 mg/kg reduced or a trend toward less bone growth was observed. The magnitude of reduction in measures of bone growth was comparable to the size of the deficit in body weight gain with EFX dose. The deficit in bone growth was reflected in lower levels of the bone formation biomarker, PINP. There were no changes in PINP levels in females at

10 mg/kg. Consistent with changes in bone morphology and strength arising mainly from reduced growth with EFX, levels of the bone resorption biomarker, CTX, were relatively stable in males and females during dosing.

Minimal to moderate atrophy of the female genital duct (uterus, cervix and vagina) was commonly seen at ≥30 mg/kg and was associated with marked decreases in uterine weight. The reduced development of genital ducts may reflect a slightly delayed sexual maturation, secondary to the deficit in body weight gain.

A single female in the 100 mg/kg group displayed symptoms associated with an immune mediated reaction, reddening of the skin (petechiae, hematoma, and/or purpura) that was diffuse on the extremities and upper body beginning at about Week 2 with recurring occurrences following each dose administration throughout the study. Additionally, systemic exposure was transiently lower for this animal and body weight loss was greater than other females treated with 100 mg/kg. At histopathological examination, this female presented a moderate chronically active crescentic glomerulonephritis associated with mild tubular hyaline casts. These renal findings were associated with substantial proteinuria, hematuria and creatinuria; serum BUN or creatinine levels were not different from controls. The occurrence of this histopathological finding has been reported for monkeys following injection of protein-based biologics that is generally immune mediated (Frazier & Obert 2018). Glomerulonephritis associated with the renal findings was considered adverse to the animal although these reactions have a limited prediction of the risk in humans (Vahle 2018, Kronenberg et al. 2017).

Following SC administration, T_{max} of EFX ranged between 8- and 48-hours post-dose on Days 1, 64 and 113. The systemic exposure, as assessed by C_{max} and $AUC_{(0\text{-}t)}$, increased in a near dose-proportional manner between 10 and 100 mg/kg. $AUC_{(0\text{-}t)}$ values on Days 64 and 113 indicated modest accumulation at all dose levels (1.1-1.8 fold) relative to the first dose (Day 1). No differences in systemic exposure (C_{max} and $AUC_{(0\text{-}t)}$) were observed between males and females on Days 1, 64 and 113.

Based on results available, the NOAEL was considered to be 30 mg/kg for males and females. The combined $C_{max}/AUC_{0\text{-}24hr}$ values at the end of the study (Day 113) at the NOAEL was 236 µg/mL and 17,400 hr*µg/mL, respectively.

1.3.3.2. Genotoxicity (Mutagenicity)

EFX is a recombinant protein made up entirely of naturally occurring amino acids and contains no inorganic or synthetic organic linkers or other non-protein portions. Thus, it is highly unlikely that EFX would react directly with deoxyribonucleic acid (DNA) or other chromosomal material. In accordance with ICH S6 (R1), no genotoxicity studies have been conducted or are planned with EFX.

1.3.3.3. Carcinogenicity

No studies evaluating the carcinogenic potential of EFX have been conducted to date. However, studies were conducted to examine the potential of EFX to impact cell proliferation and mitogenicity. In the 16-week repeat dose toxicology studies, no pre-neoplastic histopathology changes were observed in the rat. These data in conjunction with the lack of cellular proliferation described above suggests that EFX would not be associated with a risk for carcinogenicity.

1.3.3.4. Developmental and Reproductive Toxicity

No studies evaluating the developmental and reproductive toxicity of EFX have been conducted to date. In the 16-week toxicity studies, no effects on male or female reproductive organs was observed in the rat. In the monkey, minimal to moderate atrophy of the female genital tract (uterus, cervix and vagina) was seen at 30 and 100 mg/kg and were associated with marked decreases of uterus weight. These findings in the female appear to be secondary to the negative caloric balance induced by EFX pharmacology (Cameron 1996, Adams et al. 2013, Talukdar et al. 2016).

1.3.3.5. Ongoing/Planned Toxicology Studies of EFX

Chronic toxicity studies in rat and monkey and reproduction and developmental toxicity studies in the rat and rabbit are ongoing. .

1.3.3.6. Margin of Safety for Clinical Dose of EFX

In the 16-week toxicity study of EFX, rats and monkeys were dosed weekly by SC injections at doses of 10, 30, or 100 mg/kg. In both species, similar toxicity was observed with effects arising from the pharmacology of EFX. The NOAEL at the end of the 16-week study was 30 mg/kg in monkeys and 100 mg/kg for rats. The AUC and maximum observed concentration (C_{max}) at the NOAEL are shown in Table 1. The exposure margins were calculated using AUC_{0-t} values for a period of 96 hours (AUC_{0-96h}) in rats and monkeys compared to 168 hours (AUC_{0-168h}) in humans, and hence were not compared based on the same time period between species. Based on the EFX PK profiles, this approach was considered acceptable as it provides more conservative estimates of exposure margins (i.e. lower calculated values) in terms of AUC_{0-t} and supported the proposed clinical doses.

Table 1. Systemic Exposures in Monkey, Rat, and Humans at the NOAEL and Margin of Safety in Humans

Species	Human Exposure Margins at Respective NOAELs							
	C_{max} (µg/mL)			Human Exposure Margins Range [b] (fold)	AUC_{0-t}[c] (µg*d/mL)			Human Exposure Margins Range [b] (fold)
	SD57	SD113			SD57	SD113		
	M+F	M	F		M+F	M	F	
Rat	211	18	120	2.9 – 33.5	765	28	319	0.9 - 24
Monkey		236		37.5		725		23
Human[a]		6.3		1		32		1

AUC = area under the drug concentration versus time curve; C_{max} = maximum observed concentration; F = females; HEMR = human exposure margins range; M = males; NOAEL = no-observed-adverse-effect level; max = maximum, min = minimum; SD = study day

Note: PK values for the monkey and rat are at the NOAEL. The values reported for the AUC (µg*hr/mL) were converted to µg*day/mL by dividing the value by 24 hr. Margin of safety is the ratio of exposure in animals to humans.

[a] Human exposure values (AUC_{0-168h}) on Day 22 in the initial Phase 1 clinical trial (20100018) in diabetic patients treated for 4 weeks with once weekly EFX via sc administration

[b] Exposure margins range represents the lowest and highest margins for each exposure parameter, where applicable

[c] Exposure data represented as AUC_{0-96h} for rats and monkeys and AUC_{0-168h} for humans.

When considering all the relevant animal data including both Day 57 and Day 113 exposure data in male and female rats, EFX exposure margins at the proposed maximum clinical dose ranged from approximately 2.9- to 37.5-fold based on C_{max} and approximately 0.9- to 24-fold based on AUC_{0-t}.

1.4. Clinical Trials of EFX

EFX has been evaluated in Phase 1 single- and multiple-ascending dose studies up to 4-weeks treatment duration in type 2 diabetes (T2D) patients. In the Phase 1a single-ascending dose study, the highest dose tested was 210 mg administered subcutaneously. In the Phase 1b multiple-ascending dose study, the highest dose tested was 140 mg QW SC. In both studies, the drug was generally well tolerated. The nonclinical safety data and safety profile of the doses tested in the Phase 1a/1b studies support the safety of doses intended for clinical testing in the proposed Phase 2a study in NASH patients. A summary of the results of the Phase 1 studies are described below and are described in further detail in the current IB.

1.4.1. Study 20100015 - Phase 1a Single Ascending Dose Study in Diabetic Patients

Study 20100015 was a Phase 1, randomized, double-blind, placebo-controlled, ascending single dose study whose objective was to evaluate the safety, tolerability, PK, and pharmacodynamics (PD) of EFX in subjects with type 2 diabetes. Primary endpoints included subject incidence of treatment-emergent adverse events (TEAEs), laboratory analyte markers of safety, vital signs, and ECGs and incidence of anti-EFX antibodies. Secondary endpoints included EFX serum PK, and multiple PD parameters based on the known metabolic effects of EFX including: fasting or post-meal levels of glucose, insulin and C-peptide, lipoproteins, and markers of lipid metabolism.

1.4.1.1. Subject Disposition

Eligible subjects were to be enrolled into one of 7 dose cohorts. For cohorts 1, 2, 3, 4, 5, and 7, 8 subjects with T2D were to be randomized to receive EFX or placebo SC in a 3:1 ratio at dose levels of 2.1 mg, 7.0 mg, 21 mg, 70 mg, or 210 mg, respectively. In cohort 6, 8 subjects with T2D were to receive EFX or placebo intravenous (IV) in a 3:1 ratio at a dose level of 70 mg.

A total of 42 subjects (25 men, 17 women; median age of 55) received a single dose of either placebo (11 subjects) or EFX (31 subjects) and completed the study. SC doses of 2.1 mg, 7.0 mg, 21 mg, 70 mg, or 210 mg (6-7 subjects per dose level) were administered. In addition, 1 subject received a 70 mg IV dose of EFX.

1.4.1.2. Pharmacokinetic Results

Median time to reach maximal serum EFX concentrations (t_{max}) ranged from 2 to 5 days following single doses of 2.1, 7, 21, 70, or 210 mg SC EFX. For the SC cohorts, mean EFX AUC_{last} values increased 309-fold over the 100-fold dose range.

The mean half-life ranged from 3.14 to 3.38 days after SC doses of 7 to 210 mg. The mean terminal half-life ($t_{1/2,z}$) was 2.62 days after IV dosing.

1.4.1.3. Pharmacodynamic Results

A number of isolated differences were observed in the fasting metabolic parameters following single SC and IV doses of EFX. After a single 7.0 mg SC dose, compared to placebo, fasting glucose increased as much as 40%, fasting insulin increased up to 60%, and fasting glucagon increased up to 60%. At the 210 mg dose, significant decreases were seen with homeostasis model assessment (HOMA-B) (up to 40% decrease) and significant increases were seen in fasting glucagon (up to 70% increase) compared to placebo. Glucagon $AUC_{0\text{-}4hr}$ ratio to baseline increased compared to placebo at the 210 mg SC dose level after a mixed meal challenge. Otherwise, single doses of EFX did not significantly affect metabolic parameters after a mixed meal challenge. At the 210 mg SC dose, reductions were observed in total cholesterol (up to 20%) and low-density lipoprotein cholesterol (LDL-C) (up to 40%) compared to placebo. At doses of 21 mg SC and higher, increases were observed in high-density lipoprotein cholesterol (HDL-C) (up to 50%) along with reductions in triglycerides (up to 50%) compared to placebo. Increases of up to 50% were also noted in β-hydroxybutyrate with doses of 21 mg SC and higher compared to placebo. Compared to placebo, up to 2% reductions in body weight were seen following administration of EFX at doses of 21 mg SC and higher.

1.4.1.4. Safety Results

A total of 42 subjects received a single dose of either placebo (11 subjects) or EFX (31 subjects). Seven subjects (64%) receiving placebo and 17 subjects (55%) receiving EFX at any dose experienced ≥ 1 adverse event (AE) during the study. AEs reported for ≥ 3 subjects, by preferred term, were anemia (EFX: 3 [10%]; placebo: 1 [9%]); diarrhea (EFX: 3

[10%]; placebo: 1 [9%]); and increased appetite (EFX: 5 [16%]; placebo: 0 [0%]). One subject in the 70 mg IV treatment group experienced a serious adverse event (SAE) of cholecystitis; the event was considered unrelated to investigational product. The subject was hospitalized and underwent a cholecystectomy. No other SAEs were reported during the study. No subjects withdrew from the study due to an AE and no subjects discontinued investigational product. No subjects died during the study. During the washout period, 4 subjects (9%) reported AEs. These included headache (3 subjects [6%]) and syncope (1 subject [2%]). There were no SAEs reported during the washout period.

All AEs reported were either Grade 1 (mild) or Grade 2 (moderate) in severity with the exception of one Grade 3 (severe) event of syncope in the EFX 2.1 mg SC group and one Grade 3 event of cholecystitis in the EFX 70 mg IV group. Both events were considered unrelated to investigational product. One subject (9%) in the placebo group and 8 subjects (26%) in the combined EFX treatment groups experienced treatment related AEs. None of these were considered as serious. Treatment-related AEs reported for ≥ 3 subjects, by preferred term, were diarrhea (EFX: 3 [10%]; placebo: 0 [0%]) and increased appetite (EFX: 5 [16%]; placebo: 0 [0%]). During the washout period, 4 subjects (9%) reported AEs. These included headache (3 subjects [6%]) and syncope (1 subject [2%]). There were no SAEs reported during the washout period. There were no trends indicative of clinically important treatment-related laboratory abnormalities. No clinically significant abnormalities in vital signs and ECGs were noted in subjects treated with EFX or placebo. Anti-EFX binding, non-neutralizing antibodies in 4 subjects did not appear to affect the safety profile of EFX.

Study 20100015 was closed prior to completing the enrollment of cohort 6 (70 mg IV) and initiating the enrollment of cohort 7 (420 mg SC). Only the sentinel pair was dosed in cohort 6. Upon review of the initial safety data from cohorts 5 and 6, which included the three events of Grade 2 diarrhea in the 210 mg SC group and the one event of Grade 3 cholecystitis in the 70 mg IV group, the decision was made not to dose escalate to cohort 7, and no further subjects were enrolled into cohort 6.

1.4.1.5. Conclusion

Overall, EFX appeared to be well tolerated at doses up to 70 mg SC. At doses up to 70 mg, subject incidences of overall AEs and treatment-related AEs did not differ notably between treatment groups, and no trends indicative of clinically important adverse effects of EFX on laboratory or other safety-related clinical parameters were apparent.

At doses of 210 mg SC and 70 mg IV, dose stopping criteria were met by 3 subjects (Grade 2 diarrhea) and 1 subject (Grade 3 cholecystitis), respectively. The decision was made not to escalate to the planned 420 mg SC group and to discontinue enrollment in the 70 mg IV group. Binding, non-neutralizing anti-EFX antibodies were detected in 4 subjects after treatment with EFX. EFX-related effects on PD parameters were noted at doses higher than 21 mg SC and included decreases in total cholesterol, LDL-C, and triglycerides, and increases for HDL-C. Smaller changes were seen at the 7 mg SC dose. Fasting glucose, HOMA-B, and glucagon also showed response to EFX administration. Small reductions in

body weight were also noted with the 21 mg SC and higher doses. EFX was absorbed into systemic circulation with a median t_{max} of 2 to 5 days following SC dosing.

1.4.2. Study 20100018 - Phase 1b Multiple Ascending Dose Study in Diabetic Patients

Study 20100018 was a Phase 1b multicenter, randomized, double-blind, placebo-controlled, ascending multiple-dose study in subjects with type 2 diabetes. Doses of 7 mg every 2 weeks (Q2W), 7 mg QW, 21 mg Q2W, 21 mg QW, 70 mg Q2W, 70 mg QW, 140 mg Q2W, and 140 mg QW administered for 1 month were tested. The primary endpoints were subject incidence of TEAE, laboratory analyte markers of safety, vital signs, ECG, and subject incidence of anti-EFX antibodies. Secondary and exploratory endpoints included multiple metabolic PD and PK assessments.

1.4.2.1. Subject Disposition

Eligible subjects were to be enrolled into 1 of the 9 cohorts and randomized to receive EFX or placebo in a ratio of 3:1 (cohorts 1 to 8) or 1:1 (cohort 9). The planned sample size was approximately 64 subjects in cohorts 1 to 8 (8 subjects in each cohort) and 60 subjects in cohort 9. EFX or equivalent dose of placebo was administered SC Q2W or QW for 4 weeks. The planned dose escalation for AKR 001 in cohorts 1 through 8 was 7 mg Q2W, 7 mg QW, 21 mg Q2W, 21 mg QW, 70 mg Q2W, 70 mg QW, 140 mg Q2W, and 140 mg QW, respectively. Subjects in cohort 9 were to receive 70 mg EFX QW.

A total of 86 patients were enrolled in 1 of 9 cohorts and randomized to receive EFX or placebo in a ratio of 3:1 (cohorts 1 to 8) or 1:1 (cohort 9). Seventeen patients were withdrawn from the study during the washout period before receiving EFX. The remaining 69 subjects were randomized in cohorts 1 to 8 including 52 subjects in the EFX groups and 17 subjects in the placebo group. Of the 52 subjects in the EFX groups; 6 subjects each were in cohort 1 and cohorts 3 to 7, 7 subjects in cohort 2, and 9 subjects in cohort 8. A total of 63 subjects (91.3%) completed investigational product as per the dosing regimen for 4 weeks. Six subjects (8.7%) discontinued investigational product; 1 subject in EFX cohort 2, 4 subjects in EFX cohort 8, and 1 subject in the placebo group. All 6 subjects discontinued investigational product because of AEs. A total of 63 subjects (92.8%) completed the study. Of the 5 subjects (7.2%) who discontinued the study; 2 subjects (2.9%) withdrew consent and 1 (1.4%) subject each discontinued due to protocol specified criteria, sponsor decision, or loss of follow up. All 5 subjects who discontinued the study were in EFX groups (1 subject in cohort 4, 4 subjects in cohort 8). The study was terminated before enrollment of subjects into cohort 9 (70-mg EFX QW) upon review of the aggregate PD data from cohorts 1 to 8 which confirmed the glucose lowering effect of EFX. Therefore, cohort 9 was deemed unnecessary by the sponsor.

1.4.2.2. Pharmacokinetic Results

Based on linear regression analysis of dose-normalized log-transformed C_{max} and AUC_{inf} values, EFX exhibited linear PK after Q2W or QW SC administration across the dose range

of 7-mg to 140-mg. Median t_{max} values following Q2W or QW SC administration of 7-mg to 140-mg ranged from 2 to 3.5 days for all cohorts. There was minimal accumulation of EFX following 2 doses administered Q2W, with mean accumulation ratios ranging from 0.86 to 1.94. Following 4 doses administered QW, moderate accumulation was observed, with mean accumulation ratios ranging from 1.56 to 3.61. There was no discernible trend between dose and accumulation ratio. For those subjects who developed anti-EFX antibodies, there was no detectable change in PK of EFX by comparison with ADA negative subjects in the respective dose groups.

1.4.2.3. Pharmacodynamic Results

Q2W Dosing Regimen:

Fasting glucose increased over time as percent change compared with placebo in the 7-mg, 21-mg, and 140-mg groups (up to 29%, 32%, and 53% increase, respectively). Fasting insulin increased over time in the 7-mg group (up to 59% increase) and decreased over time in the 140-mg group (up to -41% decrease). The percent change from baseline in homeostasis model assessment of insulin resistance (HOMA-IR) increased over time in the 7-mg group (up to 75% increase) and decreased over time in the 140-mg group (up to -38% decrease). Improvement in beta cell function was evident following 140mg doses, with HOMA-B decreasing by up to 50%. Inconsistent with this, fasting C-peptide levels decreased by up to 36% in the 140-mg group. Fasting glucagon increased by up to 36%, 68%, and 31%, respectively in the 7 mg, 70 mg, and 140 mg groups.

Following a mixed meal tolerance test on Day 18, compared with placebo, insulin and C-peptide AUC from 0 to 4 hours (AUC_{0-4hr}) decreased by 27% and 18% respectively in the 140-mg group. Glucagon AUC_{0-4hr} increased 32% in the 70 mg group.

Fasting total cholesterol increased by up to 25% over time in the 140 mg group. HDL-C increased by up to 28% over time in the 21 mg and 70 mg groups, and by up to 40% in the 140 mg group. Fasting triglyceride decreased by up to 43% and 56% respectively over time in the 70 mg and 140 mg groups.

Fasting levels of β-hydroxybutyrate increased by 166% and 235% respectively in the 70-mg and 140-mg groups.

Body weight appeared to decrease in a dose-responsive manner.

QW Dosing Regimen:

Fasting glucose decreased by up to 34% and 32% respectively compared with placebo, in the 70-mg and 140-mg groups. Fasting insulin decreased by up to 54% and 51% respectively in the 70-mg and 140-mg groups. Consistent with this, fasting C-peptide decreased over time in the 21-mg, 70-mg, and 140-mg groups by up to 32%, 40% and 44% respectively. The improvements in insulin sensitivity underlying these changes were reflected by reductions of up to 63% and 60% in HOMA-IR respectively in the 70-mg and 140-mg groups. The improvement in insulin sensitivity occurred despite increases over time in fasting glucagon

for all the EFX groups: up to 55%, 50%, 38%, and 64% higher, respectively for 7-mg, 21-mg, 70-mg, and 140-mg groups.

Following a mixed meal tolerance test on Day 25, glucose $AUC_{0\text{-}4hr}$ was 29% and 30% lower in the 70-mg and 140-mg groups respectively. Insulin and C-peptide $AUC_{0\text{-}4hr}$ were – respectively 28% and 20% lower in the 21-mg group, and 47% and 36% lower in the 140-mg group. Free fatty acids $AUC_{0\text{-}4hr}$ was decreased by 30% in the 70-mg group. Glucagon $AUC_{0\text{-}4hr}$ was increased by 43% in the 7-mg group.

Fasting total cholesterol decreased by up to 16% and 17% respectively in the 70-mg and 140-mg groups. Fasting LDL-C decreased by 33% over time in the 140-mg group. Fasting HDL-C increased by up to 31%, 43%, 61%, and 48% respectively in the 7-mg, 21-mg, 70-mg, and 140-mg groups. Fasting triglycerides decreased by up to 49%, 55%, 69%, and 65% respectively in the 7-mg, 21-mg, 70-mg, and 140-mg groups.

Fasting levels of β-hydroxybutyrate increased by up to 271% in the 140-mg AKR-group.

Body weight appeared to decrease in a dose-responsive manner.

1.4.2.4. Safety Results Describing the Combined Q2W and QW Datasets

A total of 69 subjects received at least a single dose of either EFX (52 subjects) or placebo (17 subjects) and were included in the safety analysis set. Of these, 55 subjects (EFX group: 43 [82.7%]; placebo group: 12 [70.6%]) had ≥ 1 AE during the study.

Of the patients who received the 70 mg SC dose or less (the highest dose proposed in Protocol AK-US-001-0101), 3 AEs of Grade 3 or above were observed and considered not related to investigational product: two instances of transient hyperglycemia in one subject (7 mg QW dose), and one instance of transient diarrhea (7 mg QW dose).

Six subjects (8.7%) discontinued investigational product; one subject in cohort 2, four subjects in cohort 8, and one subject in the placebo group. All six subjects discontinued investigational product because of AEs. Four of the patients who discontinued were in the 140 mg QW group. The reasons for discontinuation by each of the four subjects dosed at 140 mg QW were diarrhea; vomiting; tremor; and tremor/nausea, respectively. The remaining two discontinuations (one following treatment with 7mg QW; one on placebo) were attributed by the investigator to hyperglycemia and were considered unrelated to investigational product. Subjects were washed off anti-diabetic medications two weeks prior to the first dose and remained so until end of study. A summary of the treatment-related AEs and withdrawals is provided in Table 2.

There were no trends indicative of clinically important treatment-related laboratory abnormalities. No trends in clinically significant abnormalities in vital signs and ECG were noted in subjects treated with EFX or placebo. At the end of study visit (EOS), 7 subjects (13.5%) tested positive for anti-EFX binding, non-neutralizing antibodies dosing with EFX. Three of these 7 subjects returned for a follow-up antibody sample collected at least 2 months after the End of Study (EOS) visit. All 3 subjects tested negative for anti-EFX antibodies.

Table 2. Treatment-Related Adverse Events and Withdrawals

	Placebo	EFX							
		QW				Q2W			
	QW/Q2W (N=17)	7 mg (N=7)	21 mg (N=6)	70 mg (N=6)	140 mg (N=9)	7 mg (N=6)	21 mg (N=6)	70 mg (N=6)	140 mg (N=6)
Subjects reporting all-grade IP-related TEAEs (n)	3	2	4	5	8	3	3	2	3
Grade 2-4*	0	0	1	0	2	0	1	0	1
Adverse events with two or more observations									
Nausea (all grade)	0	1	3	0	6	0	2	1	2
Grade 2-4	0	0	0	0	0	0	0	0	2
Diarrhea (all grade)	1	1	0	2	2	0	1	1	1
Grade 2-4	0	0	0	0	0	0	1	0	0
Change in appetite† (all grade)	0	1	0	2	5	0	1	0	0
Grade 2-4	0	0	0	0	0	0	0	0	0
Vomiting (all grade)	0	0	0	0	3	0	1	0	2
Grade 2-4	0	0	0	0	1	0	0	0	1
Gastrointestinal, other‡ (all grade)	1	0	1	0	5	2	1	1	0
Grade 2-4	0	0	0	0	0	0	0	0	0
Tremor (all grade)	0	0	0	0	4	0	0	0	0
Grade 2-4	0	0	0	0	1	0	0	0	0
Headache (all grade)	1	0	0	0	1	1	1	0	0
Grade 2-4	0	0	0	0	0	0	0	0	0
Injection-site rash or erythema (all grade)	0	0	1	2	1	0	0	0	1
Grade 2-4	0	0	1	0	0	0	0	0	0
Withdrawals	0	0	0	0	4§	0	0	0	0

* - CTCAE toxicity grades
# - a single event of the following AEs was observed in the trial: dizziness (140 mg QW), dysgeusia (140 mg QW), musculoskeletal pain (7 mg Q2W), muscle spasms (140 mg QW), ventricular extrasystoles (140 mg QW), hyperhidrosis (140 mg QW), flushing (21 mg Q2W); all events Grade 1
† - includes increased appetite, decreased appetite, and hunger
‡ - includes constipation, dyspepsia, abdominal distension, abdominal pain, abdominal tenderness, and epigastric discomfort
§ - reason for withdrawals: nausea and tremor (1 subject), diarrhea (1 subject), nausea (1 subject), tremor (1 subject)

1.4.2.5. Conclusion

Overall, EFX appeared to be well tolerated at doses up to 70 mg SC. At doses up to 70 mg, subject incidences of overall AEs and TEAEs did not differ notably between treatment groups, and no trends were apparent indicative of clinically important adverse effects of EFX on laboratory or other safety-related clinical parameters. There were no patient deaths and no SAEs attributable to treatment with EFX. The most common AEs were gastrointestinal disorders, such as mild diarrhea and nausea.

Following administration of a 210 mg dose, 3 of 6 subjects reported diarrhea and 4 of 6 subjects reported increased appetite. Neither diarrhea nor increased appetite were reported for subjects receiving any other dose of EFX. No other AEs were reported by more than one subject. All AEs were reported as either mild or moderate, with the exception of two AEs graded as severe but considered unrelated to the investigational product by the investigators. One subject experienced a severe AE of vasovagal syncope secondary to blood draw following randomization to the 2.1mg cohort, but prior to receiving any investigational product. One subject who received EFX 70 mg IV had a severe AE of cholecystitis initially reported as abdominal pain beginning on Day 11. The subject thereafter reported having experienced intermittent abdominal pain for many years. Findings from a subsequent cholecystectomy were consistent with chronic cholecystitis.

Seven subjects developed anti-EFX non-neutralizing antibodies post baseline. Three of these 7 subjects returned for a follow-up antibody sample collected at least 2 months after the EOS Visit, with all 3 subjects testing negative for anti-EFX antibodies. EFX exhibited linear PK and increased dose-proportionally from 7-mg to 140-mg following Q2W or QW SC administration. In general, minimal accumulation (< 2-fold) was observed after 2 SC doses administered Q2W, while moderate accumulation (approximately 2-fold) was observed after 4 SC doses administered QW. The presence of anti-EFX binding antibodies did not appear to affect PK of EFX.

The extent of improvements in markers of insulin sensitivity elicited by Q2W dosing was less than for QW dosing. Likewise, the improvement in lipoprotein profile was greater for QW than Q2W and was evident at lower doses. In the context of NASH, greater insulin sensitivity would be expected to reduce flux of fatty acids from adipose tissue to liver, the major source of lipid entering the liver. At the same time, improved adipose insulin sensitivity is associated with greater uptake of plasma triglyceride whether of liver (VLDL) or dietary (chylomicron) origin. The substantial reductions in plasma triglyceride elicited by QW EFX are consistent with these effects, and potentially with reduced synthesis and secretion of VLDL by liver. Overall the improvements in metabolic status of the T2D subjects, particularly insulin sensitivity of adipose tissue, and in lipoprotein profile indicate potential for therapeutic benefit in NASH with QW dosing of EFX.

1.5. Rationale for this Study

Given FGF21's well-documented beneficial effects on metabolic parameters, FGF21 has recently emerged as a promising drug candidate for metabolic diseases, including NASH. FGF21 has many of the key characteristics of an optimal NASH therapy as a result of its well-documented effects in improving insulin sensitivity and lipoprotein levels, reducing liver fat and inflammation, as well as inhibiting fibrosis. In particular, EFX's dual actions of addressing both the underlying NASH disease driver (i.e., increased liver fat and fat oxidation) and downstream NASH pathology (i.e. cellular stress, inflammation and fibrosis), is well suited to patients with F3 and F4 fibrosis, not just patients with early-stage disease.

Results from a Phase 2 trial of BMS-986036, an investigational PEGylated analog of FGF21, provide important validation for FGF21 agonism as a NASH therapy (Sanyal et al. 2019). In this study, 74 patients with biopsy-confirmed NASH (stages F1-F3) received 16 weeks of daily or weekly subcutaneous injections of BMS-986036 or placebo. The study achieved its primary endpoint of significant reduction in liver fat versus placebo, as measured non-invasively by magnetic resonance imaging – proton density fat fraction (MRI-PDFF). Absolute fat levels in the daily and weekly dosing cohorts fell 6.8% and 5.2%, respectively, compared to 1.3% in the placebo group. These reductions translated into relative reductions of 38% and 26%, respectively, compared to 6% in the placebo group. Statistically significant improvements were also seen in prespecified exploratory endpoints including biomarkers of fibrosis, lipoprotein parameters and markers of liver injury. Positive changes in Pro-C3, a marker of liver fibrogenesis, and liver stiffness as measured by magnetic resonance elastography, or MRE, were also observed. The addition of Cohort C to the protocol will evaluate the effects of EFX on subjects with cirrhosis using non-invasive imaging and biomarkers of liver fibrosis.

Results from a Phase 2 trial of NGM282, an FGF19 analog, provides additional clinical validation for FGF21 agonism (Harrison et al. 2019). FGF19 and FGF21 are both members of the endocrine subfamily of FGFs. Both have been shown to activate three FGF receptors (FGFR1c/2c/3c) expressed in liver cells (mainly 2c and 3c) and adipose cells (mainly 1c). FGF19 also activates a fourth receptor, known as FGFR4, which FGF21 does not activate.

In a 12-week clinical trial in NASH patients, a 3 mg daily dose of NGM282 was shown to have a placebo-corrected absolute reduction in liver fat of 11.2% at 12 weeks, or a 67% relative reduction, as measured by MRI-PDFF. This magnitude of liver fat reduction is the largest observed to date in all NASH clinical trials.

Preclinical data suggests that FGF21 agonism of FGFR1c substantially inhibits adipose tissue lipolysis. NGM313, which was developed to mimic FGF21's effects on FGFR1c and βKlotho (but with no activity at FGFR2c and 3c), provides important clinical validation that agonism of FGFR1c substantially reduces liver fat in NASH patients. NGM313 was evaluated in a single-dose, open-label Phase 1b trial in 25 obese, insulin resistant subjects with NAFLD. Although it is an open label study, a 37% relative reduction of liver fat at 5 weeks, along with a 48% reduction in fasting triglycerides at 4 weeks, suggests that agonism of FGFR1c will result in meaningful liver fat reduction in NASH patients (Shankar et al. 2018).

We hypothesize that EFX, by virtue of its balanced agonism across FGFR1c/2c/3c will act on both liver and adipose tissue to reduce liver fat substantially, in turn mitigating hepatocyte stress. By combining these metabolic improvements with the direct anti-inflammatory and antifibrotic effects evident with FGF21, EFX is expected to deliver clinically meaningful resolution of NASH.

The dose and length of administration of the drug in study AK-US-001-0101 are supported by safety data from nonclinical studies and from prior Phase 1a/1b studies in T2D patients. The primary endpoint of the study will be the absolute change from baseline in hepatic fat

fraction assessed by MRI-PDFF at Week 12. Non-invasive imaging modalities that assess hepatic fat content are acceptable as study endpoints for early Phase 2 trials (Noncirrhotic Nonalcoholic Steatohepatitis With Liver Fibrosis: Developing Drugs for Treatment, Guidance for Industry, December 2018).

The potential to observe early histologic changes at Week 12 is supported by the Phase 2 trial of NGM282. Nineteen subjects with biopsy-confirmed NASH completed 12 weeks of treatment with paired biopsies. Histopathology of 13 (68%) of these biopsies revealed anti-fibrotic activity at Week 12, with biopsies from eight subjects (42%) showing a reduction of ≥ 1 fibrosis stage (Harrison et al. 2019).

The current regulatory standard for assessing fibrosis referenced in the Guidance for Industry is liver biopsy. As such, improvement in liver fibrosis without worsening of NASH as assessed by liver biopsy will be evaluated in a subset of subjects who show a significant response to treatment (≥ 30% fat reduction by MRI-PDFF at Week 12). Liver biopsy is limited by cost, sampling error, and variability in subjective histopathological interpretation. Due to these limitations, the development and validation of noninvasive markers of liver injury has emerged as a clinical and research priority. A key exploratory objective of the study is to correlate these noninvasive markers with liver histology and determine their utility for monitoring and ultimately predicting histological responses that occur during the course of the study.

Currently there are no approved drugs for the treatment of NASH, and there remains a clear unmet medical need to slow progression of, halt, or reverse NASH. As fibrosis is seen as the strongest predictor of adverse clinical outcomes in NASH, the Main Study will enroll biopsy-proven noncirrhotic NASH patients with (fibrosis stage 1 to 3 and a NAS of ≥ 4) and Cohort C will enroll patients with compensated cirrhosis with fibrosis stage 4.

1.6. Rationale for Selection of Dose

As described in Section 1.4.1 and Section 1.4.2, in the Phase 1a/1b studies, EFX appeared to be well tolerated at doses up to 70 mg SC. There were no patient deaths and no SAEs attributable to treatment with EFX. The most common AEs were gastrointestinal disorders, such as mild diarrhea and nausea.

Exploratory analyses for Study 20100018 (Section 1.4.2) were conducted by Akero to demonstrate the dose-related change from baseline for lipoproteins and markers of insulin sensitivity, observed following administration of 21 mg and 70 mg QW doses of EFX, compared to placebo. Significant improvements for each marker of insulin sensitivity were observed at the 70 mg QW dose, consistent with agonism of FGFR1c in adipose tissue.

The analysis indicates maximal or near maximal effects were observed by the third dose of 70 mg QW for lipoproteins, and by the fourth dose for markers of insulin sensitivity. Reductions in triglyceride and increases in HDL-C were significant at all time points from Day 4 through Day 57, while non-HDL-C was significantly lower from Day 15 through Day 57. Taken together with published clinical data for third-party FGF21 analogs, the

time-course and magnitude of changes in lipoproteins observed at the 70 mg QW dose suggest that EFX has the potential to rapidly and durably reduce liver fat in patients with NASH. Notably, EFX's effects appear to be sustained for three weeks after the final dose, including significant increases of 39% in HDL-C and significant reductions of 28% and 67% in non-HDL-C and triglycerides, respectively, observed on Day 43.

At 21 mg QW, there were also indications of improved sensitivity to insulin, with a significantly lower level of C-peptide observed after the fourth dose, and a trend toward lower levels of insulin and a lower calculated value of HOMA IR. Although liver fat was not measured in Study 20100018, the magnitude and robustness of effects on lipoproteins at 21 mg and 70 mg QW will likely translate into substantial reductions in liver fat with longer term treatment.

Efficacy for the proposed Phase 2a study is predicted based on PK/PD modeling. The doses to be tested are expected to mediate potentially beneficial changes in PD parameters including triglycerides and LDL. PK/PD modeling suggests that the dose range selected will achieve approximately 45% to 90% of the maximum effect on triglycerides and LDL.

EFX will be administered QW at doses ranging from 28 mg/day to 70 mg/day for up to 16 weeks. Subjects with advanced cirrhotic disease in Cohort C will receive 50 mg/day based on PK/PD modeling of data from the multiple ascending dose Study 20100018. As presented in Section 1.3.3.6, a dose of 70 mg of EFX results in exposure margins ranging from approximately 2.9- to 37.5-fold based on C_{max} and approximately 0.9- to 24-fold based on AUC_{0-t}.

At doses up to 70 mg, subject incidences of overall AEs and treatment-related AEs did not differ notably between treatment groups, and no trends were apparent indicative of clinically important adverse effects of EFX on laboratory or other safety-related clinical parameters. Of the patients who received the 70 mg SC dose or less (the highest dose proposed in this protocol), 3 AEs of Grade 3 or above were observed and considered not related to investigational product: two instances of transient hyperglycemia in one subject (7 mg QW dose), and one instance of transient diarrhea (7 mg QW dose). The withdrawals due to hyperglycemia are probably attributable to all subjects having been washed off all their anti-diabetic medications for 14 days prior to first dose of EFX. Based on these results, the highest planned SC dose level to be tested is 70 mg SC QW.

1.7. Compliance

This study will be conducted in compliance with this protocol, Good Clinical Practice (GCP), and all applicable regulatory requirements.

2. STUDY OBJECTIVES

2.1. Primary Objectives

The primary objective of the **Main Study** is:

- To evaluate the absolute change from baseline in hepatic fat fraction assessed by Magnetic Resonance Imaging - Proton Density Fat Fraction (MRI-PDFF) at Week 12

The primary objective of **Cohort C** of this study is:

- To assess the safety and tolerability of EFX in NASH subjects with compensated cirrhosis

2.2. Secondary Objectives

The secondary objectives of the **Main Study** are:

- To evaluate percent change from baseline in hepatic fat fraction assessed by MRI-PDFF at Week 12
- To evaluate the responder: subjects who achieved a clinically meaningful relative reduction of at least 30% in liver fat content as measured by MRI-PDFF at Week 12
- To evaluate the responder based on NAFLD Activity Score (NAS) system: subjects who had a decrease of ≥ 2 points in NAS with at least a 1-point reduction in either lobular inflammation or hepatocellular ballooning and with no concurrent worsening of fibrosis stage
- To evaluate the change from baseline in ALT at Week 12
- To assess the safety and tolerability of EFX in subjects with NASH

The secondary objectives of **Cohort C** of this study are:

- To evaluate the change from baseline in liver stiffness as evaluated by FibroScan® at Week 16
- To evaluate the change from baseline in non-invasive plasma (serum) fibrosis biomarkers including liver fibrosis by ELF™ Test score and pro-C3 levels

2.3. Exploratory Objectives

The exploratory objectives are:

- Change from baseline in markers of liver injury and function including, aspartate aminotransferase (AST), alkaline phosphatase (ALP), gamma glutamyl transferase (GGT), bilirubin, International Normalized Ratio (INR), albumin, Model for End-Stage Liver Disease (MELD) score, and Child Pugh (CP) score
- Change from baseline in body weight and waist-to-hip ratio
- MRI-PDFF based quantitation of hepatic fat (Main Study Only)

- Changes from baseline in noninvasive plasma (or serum) biomarkers including liver fibrosis by Enhanced Liver Fibrosis (ELF™) Test score, and by pro-C3 levels (Main Study Only)
- Change from baseline in bone mineral density of the lumbar spine, femoral neck, and total hip as measured by DXA
- The proportion of subjects – whose fibrosis regresses by ≥ 1-stage according to the NASH CRN classification
- The proportion of subjects who had no worsening in NAS with improvement in fibrosis stage
- The proportion of subjects who had 2 points improvement in NAS with no worsening in fibrosis stage
- The proportion of subjects who had ≥ 1 point improvement in NAS, fibrosis, steatosis lobular inflammation or hepatocellular ballooning
- Changes from baseline in health-related quality of life (HRQoL)
- Changes from baseline in lipoprotein profile and markers of lipid metabolism, insulin sensitivity and glycemic control
- Change from baseline in body fat distribution as measured by DXA total body composition
- Change from baseline in corrected T1 (cT1), an MRI-based metric of liver fibro-inflammation at Week 12 (Main Study Only)
- Plasma concentrations of EFX
- To evaluate decompensated liver disease events including ascites, hepatic encephalopathy (HE), or variceal bleeding (Cohort C Only)

3. INVESTIGATIONAL PLAN

3.1. Overall Study Design and Plan

This is a Phase 2a, randomized, double-blind, placebo-controlled study evaluating the safety and efficacy of EFX in subjects with NASH.

Main Study: Subjects meeting the study's entry criteria will be randomly assigned in a 1:1:1:1 ratio into 4 treatment groups as shown in Figure 2 below:

Figure 2. Study Schema – Main Study

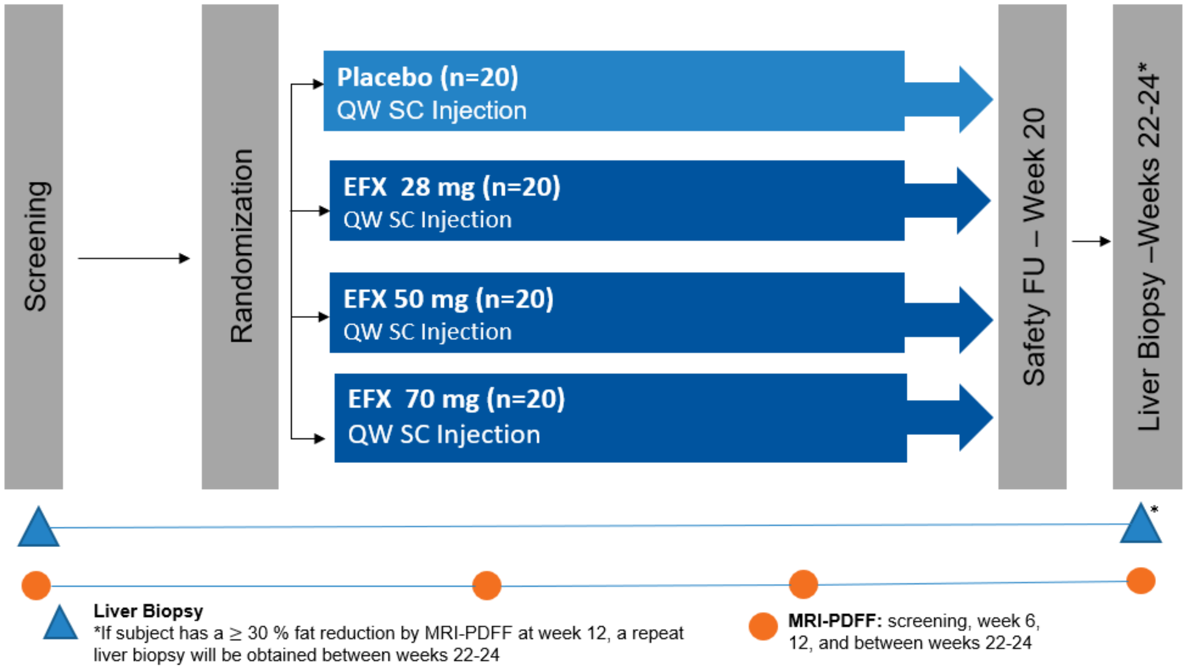

Randomization will be stratified by baseline hepatic fat fraction (< 15% vs ≥ 15%) and F1 fibrosis score. Study drug will be administered subcutaneously once weekly (QW) for a total of 16 weeks. After 16 weeks of treatment, a safety follow-up visit will occur at Week 20. Subjects with ≥ 30% relative fat reduction on MRI-PDFF at week 12 will be required to return between weeks 22 - 24 for a repeat liver biopsy and MRI-PDFF.

Cohort C: Subjects meeting the study's entry criteria will be randomly assigned in a 1:2 ratio into 2 treatment groups as shown in Figure 3 below:

Figure 3. Study Schema – Cohort C

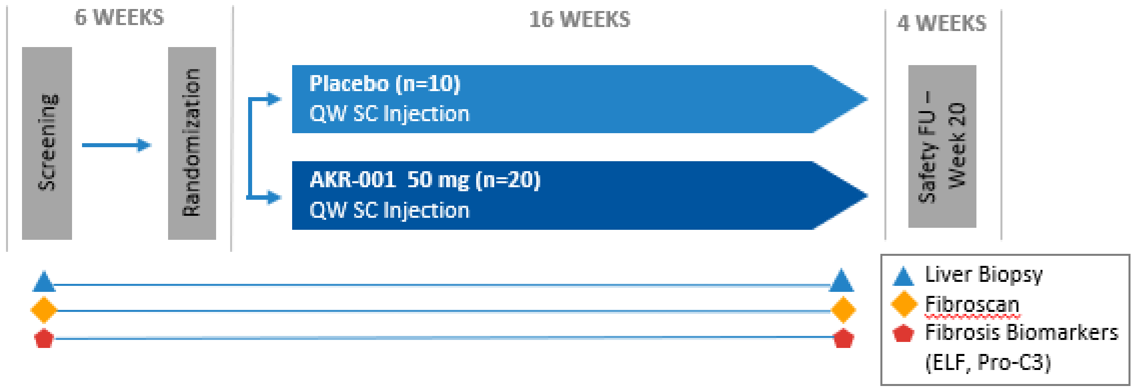

Following 16 weeks of treatment, subjects will be given the option to collect an end of treatment biopsy.

3.2. Study Duration and Number of Centers

Main Study: Participation in the study can last up to 30 weeks, which includes 6 weeks for screening, 16 weeks on-treatment, 4 weeks of safety follow-up, and an additional 4 weeks for a repeat liver biopsy and MRI-PDFF in eligible subjects.

Cohort C: Participation in the study can last up to 26 weeks, which includes 6 weeks for screening, 16 weeks on-treatment, and 4 weeks of safety follow-up.

This study will be conducted at approximately 30 sites in the U.S. Additional sites may be added, depending on subject accrual rates.

3.3. End of Study

Main Study: The end of the study is considered to be the completion of the Safety Follow-up Visit (Week 20) or the Repeat Biopsy (Week 22 - 24) for subjects who are eligible.

Cohort C: The end of the study is considered to be the completion of the Safety Follow-up Visit (Week 20).

3.4. Early Termination

If a subject discontinues study drug, every attempt should be made to perform the Early Termination (ET) assessments and the Safety Follow-up assessments. The Safety Follow-up assessments should be completed 4 weeks after the last dose of study drug.

Subjects with ongoing clinically significant clinical or laboratory findings will be followed until the finding is resolved or medically stable; reasonable attempts will be made to follow-up with subjects. The subject's participation will end once all study assessments and follow-up has been completed.

The ET and Safety Follow-up assessments are outlined in the Schedule of Assessments (Appendix A and Appendix B).

3.5. Post Study Care

There is no offered post study care.

4. STUDY POPULATION SELECTION

4.1. Study Population

The Main Study will enroll approximately 80 subjects with biopsy-proven NASH. F1 fibrosis will comprise of approximately 35% of total subject population.

Cohort C will enroll approximately 30 subjects with biopsy-proven NASH. All subjects in Cohort C will have F4 fibrosis.

4.2. Inclusion Criteria

Main Study: Subjects must meet all the following inclusion criteria to be eligible for study participation:

1. Males and non-pregnant, non-lactating females between 18 - 80 years of age inclusive, based on the date of the screening visit.
2. BMI > 25 kg/m^2 (unless the subject has biopsy-proven NASH documented within the last 2 years).
3. Must have confirmation of ≥ 10% liver fat content on MRI-PDFF at screening.
4. Biopsy-proven NASH. Must have had a liver biopsy within 180 days of randomization with fibrosis stage 1 to 3 and a NAS of ≥ 4 with at least a score of 1 in each of the following NAS components:

    a. Steatosis (scored 0 to 3),
    b. Ballooning degeneration (scored 0 to 2), and
    c. Lobular inflammation (scored 0 to 3).

5. Must have no evidence of worsening of ALT and AST (within 50%) measurements at the screening (-6 weeks) and pre-baseline (-2 weeks) visits.

6. Screening laboratory parameters, as determined by the central laboratory:

    a. Estimated glomerular filtration rate (eGFR) ≥ 60 mL/min, as calculated by the Cockcroft-Gault equation;
    b. HbA1c ≤ 9.5% (or serum fructosamine ≤ 381 μmol if HbA1c measurement is unavailable);
    c. Hemoglobin ≥ 11 g/dL;
    d. International normalized ratio (INR) ≤ 1.3, unless due to therapeutic anticoagulation;
    e. Direct bilirubin ≤ 0.3 mg/dL;
    f. Total bilirubin ≤ 1.3 x upper limit of normal ULN, unless due to an alternate etiology such as Gilbert's syndrome or hemolytic anemia;
    g. Creatinine kinase < 3 x ULN;
    h. Platelet count ≥ 150,000/μL;
    i. Serum triglyceride level ≤ 500 mg/dL;
    j. Alanine aminotransferase (ALT) < 5 x ULN;

k. Aspartate aminotransferase (AST) < 5 x ULN;
l. AST >20u/L (only required for subjects without a historical biopsy in the last 2 years confirming fibrosis 1-3 and a NAS ≥ 4);
m. Alkaline phosphatase (ALP) < 2 x ULN;

7. Subjects on Vitamin E ≥ 400 IU/day, thiazolidinediones (including, but not limited to, pioglitazone, rosiglitazone, and lobeglitazone), GLP1, or SGLT2 must be on a stable dose (defined as no changes in prescribed dose, new medications, or discontinuation) for at least 6 months prior to the diagnostic liver biopsy and screening.
8. Subjects on antidiabetic, weight loss, or lipid-modifying medication(s) must be on stable dose(s) for at least 3 months prior to the diagnostic liver biopsy through randomization.
9. Willing and able to give written informed consent prior to any study specific procedures being performed.
10. Female subjects of childbearing potential (see definition in Appendix C) must have a negative pregnancy test at screening and Baseline/Day 1.
11. Male subjects and female subjects of childbearing potential who engage in heterosexual intercourse must agree to use protocol specified method(s) of contraception as described in Appendix C.
12. FibroScan® measurement > 7.0 kPa.

Cohort C: Subjects must meet all the following inclusion criteria to be eligible for study participation:

1. Males and non-pregnant, non-lactating females between 18 - 80 years of age inclusive, based on the date of the screening visit.
2. Compensated cirrhosis (Fibrosis Stage 4) due to NASH documented by a local pathologist within 24 months of Screening with no evidence of competing etiology, and at least one co-existing or historic metabolic comorbidity. If a historical biopsy is not available within 24 months of Screening, the subject may undergo a liver biopsy to be assessed by the local or central reader and confirmed to have compensated cirrhosis (Fibrosis Stage 4).
3. FibroScan® measurement > 13.1 kPa.
4. Enhanced Liver Fibrosis (ELF) score of > 9.8.
5. Must have no evidence of worsening of ALT and AST (within 50%) measurements at the screening (-6 weeks) and pre-baseline (-2 weeks) visits.

   a. A single retest of the pre-baseline labs is permitted only if there is reason to believe the initial value was erroneous due to a sample processing error or due to an extenuating circumstance. An average of the screening and pre-baseline value will be used and compared to the retest value. The retest value must have no evidence of worsening within 50% of the average of the screening and pre-baseline value.

6. Screening laboratory parameters, as determined by the central laboratory:

a. Estimated glomerular filtration rate (eGFR) ≥ 60 mL/min, as calculated by the Cockcroft-Gault equation;
b. HbA1c ≤ 9.5% (or serum fructosamine ≤ 381 µmol if HbA1c measurement is unavailable);
c. Hemoglobin ≥ 11 g/dL;
d. International normalized ratio (INR) ≤ 1.3, unless due to therapeutic anticoagulation;
e. Direct bilirubin ≤ 0.3 mg/dL;
f. Total bilirubin ≤ 1.3 x upper limit of normal ULN, unless due to an alternate etiology such as Gilbert's syndrome or hemolytic anemia;
g. Creatinine kinase < 3 x ULN;
h. Platelet count ≥ 125,000/µL;
i. Serum triglyceride level ≤ 500 mg/dL;
j. Alanine aminotransferase (ALT) < 5 x ULN;
k. Aspartate aminotransferase (AST) < 5 x ULN;
l. Alkaline phosphatase (ALP) < 2 x ULN;
m. Albumin ≥ 3.5 g/dL.

7. Subjects on Vitamin E ≥ 400 IU/day, thiazolidinediones (including, but not limited to, pioglitazone, rosiglitazone, and lobeglitazone), GLP1, or SGLT2 must be on a stable dose (defined as no changes in prescribed dose, new medications, or discontinuation) for at least 6 months prior to the screening visit.
8. Subjects on antidiabetic, weight loss, or lipid-modifying medication(s) must be on stable dose(s) for at least 3 months prior to the screening visit.
9. Willing and able to give written informed consent prior to any study specific procedures being performed;
10. Female subjects of childbearing potential (see definition in Appendix C) must have a negative pregnancy test at screening and Baseline/Day 1;
11. Male subjects and female subjects of childbearing potential who engage in heterosexual intercourse must agree to use protocol specified method(s) of contraception as described in Appendix C.

4.3. Exclusion Criteria

Main Study: Subjects meeting any of the following criteria are not eligible for study participation:

1. Weight gain or loss >5% in the 3 months prior to randomization or > 10% in the 6 months prior to screening.
2. Type 1 and insulin dependent Type 2 diabetes.
3. Presence of cirrhosis on liver biopsy (stage 4 fibrosis).
4. Poorly controlled hypertension BP > 160/100.
5. Prior history of decompensated liver disease including ascites, hepatic encephalopathy (HE), or variceal bleeding.
6. Chronic hepatitis B virus (HBV) infection (hepatitis B surface antigen [HBsAg] positive).

7. Chronic hepatitis C virus (HCV) infection (HCV antibody [Ab] and HCV ribonucleic acid [RNA] positive). Subjects cured of HCV infection less than 2 years prior (based on date of RNA PCR negative confirmation following conclusion of treatment) to the screening visit are not eligible.
8. Prior or planned (during the study period) bariatric surgery (e.g., gastroplasty, roux-en-Y gastric bypass) surgery reversal or removal of intragastric balloon > 2 years prior to enrollment would be eligible.
9. Other causes of liver disease based on medical history and/or centralized review of liver histology, including but not limited to: alcoholic liver disease, autoimmune disorders (e.g., primary biliary cholangitis [PBC], primary sclerosing cholangitis [PSC], autoimmune hepatitis), drug-induced hepatotoxicity, Wilson disease, clinically significant iron overload, or alpha-1-antitryspin deficiency requiring treatment.
10. Subjects with osteoporosis, defined as a T-score of -2.5 or lower at screening.
11. History of liver transplantation.
12. Current or prior history of hepatocellular carcinoma (HCC).
13. Alcohol intake above an average limit of 2 drinks per day for women and 3 drinks per day for men as assessed by the AUDIT-C questionnaire.
14. Human immunodeficiency virus (HIV) infection.
15. Unstable cardiovascular disease. Any acute cardiovascular event in the six months prior to screening (ex: myocardial infarction, cerebral vascular accident, symptomatic rhythm disturbance (stable, controlled atrial fibrillation is allowed); or an emergency room visit or hospitalization for confirmed cardiovascular disease.
16. Life expectancy less than 2 years.
17. Subjects taking fibrates and peroxisome proliferator-activated receptor (PPAR) alpha agents (clofibrate, gemfibrozil, ciprofibrate, bezafibrate, or fenofibrate).
18. Use of any investigational medication within 30 days or within 5 half-lives prior to screening.
19. Subjects with a history of (12 months prior to screening) or current use of prescription drugs associated with liver steatosis (e.g. methotrexate, amiodarone, high-dose estrogen, tamoxifen, systemic steroids, anabolic steroids, valproic acid). Short courses of corticosteroids (less than two weeks) may be allowed prior to screening if in the investigator’s opinion they are not associated with liver steatosis or clinically relevant;
20. Positive urine drug screen for amphetamines, cocaine or opiates (e.g., fentanyl, heroin, morphine) at screening. Subjects on stable methadone or buprenorphine maintenance treatment for at least 6 months prior to screening may be included in the study. Subjects with a positive urine drug screen due to prescription medication (e.g., opiates, methylphenidate) are eligible if the prescription and diagnosis are reviewed and approved by the investigator.
21. Unable to safely undergo a liver biopsy.
22. Subjects who have contraindications to MR imaging (e.g., unmanageable claustrophobia or metal implants).
23. Concurrent participation in another therapeutic clinical study.

24. Presence of any laboratory abnormality or significant systemic or major illnesses (other than liver disease) that, in the opinion of the Investigator, compromise the subject's ability to safely participate in and complete the study including:
    a. Pulmonary disease, renal failure, organ transplantation, serious psychiatric disease, malignancy, history of substance abuse and/or a psychiatric condition requiring hospitalization and/or emergency room visit within six months of screening.
25. Unavailable for follow-up assessment or concern for subject's compliance with the protocol procedures.

Cohort C: Subjects meeting any of the following criteria are not eligible for study participation:

1. Weight gain or loss >5% in the 3 months prior to randomization or > 10% in the 6 months prior to screening.
2. Type 1 and insulin dependent Type 2 diabetes.
3. Prior history of decompensated liver disease including ascites, hepatic encephalopathy (HE), or variceal bleeding.
4. Child-Pugh Score > 6 (Class B or C), as determined at screening, unless due to therapeutic anti-coagulation
5. MELD score > 12, as determined at screening, unless due to therapeutic anti-coagulation
6. Poorly controlled hypertension BP > 160/100.
7. Chronic hepatitis B virus (HBV) infection (hepatitis B surface antigen [HBsAg] positive).
8. Chronic hepatitis C virus (HCV) infection (HCV antibody [Ab] and HCV ribonucleic acid [RNA] positive). Subjects cured of HCV infection less than 2 years prior (based on date of RNA PCR negative confirmation following conclusion of treatment) to the screening visit are not eligible.
9. Prior or planned (during the study period) bariatric surgery (e.g., gastroplasty, roux-en-Y gastric bypass) surgery reversal or removal of intragastric balloon > 2 years prior to enrollment would be eligible.
10. Other causes of liver disease based on medical history and/or centralized review of liver histology, including but not limited to: alcoholic liver disease, autoimmune disorders (e.g., primary biliary cholangitis [PBC], primary sclerosing cholangitis [PSC], autoimmune hepatitis), drug-induced hepatotoxicity, Wilson disease, clinically significant iron overload, or alpha-1-antitryspin deficiency requiring treatment.
11. Subjects with osteoporosis, defined as a T-score of -2.5 or lower at screening.
12. History of liver transplantation.
13. Current or prior history of hepatocellular carcinoma (HCC).
14. Alcohol intake above an average limit of 2 drinks per day for women and 3 drinks per day for men as assessed by the AUDIT-C questionnaire.
15. Human immunodeficiency virus (HIV) infection.

16. Unstable cardiovascular disease. Any acute cardiovascular event in the six months prior to screening (ex: myocardial infarction, cerebral vascular accident, symptomatic rhythm disturbance (stable, controlled atrial fibrillation is allowed); or an emergency room visit or hospitalization for confirmed cardiovascular disease.
17. Life expectancy less than 2 years.
18. Subjects taking fibrates and peroxisome proliferator-activated receptor (PPAR) alpha agents (clofibrate, gemfibrozil, ciprofibrate, bezafibrate, or fenofibrate).
19. Use of any investigational medication within 30 days or within 5 half-lives prior to screening.
20. Subjects with a history of (12 months prior to screening) or current use of prescription drugs associated with liver steatosis (e.g. methotrexate, amiodarone, high-dose estrogen, tamoxifen, systemic steroids, anabolic steroids, valproic acid). Short courses of corticosteroids (less than two weeks) may be allowed prior to screening if in the investigator’s opinion they are not associated with liver steatosis or clinically relevant;
21. Positive urine drug screen for amphetamines, cocaine or opiates (e.g., fentanyl, heroin, morphine) at screening. Subjects on stable methadone or buprenorphine maintenance treatment for at least 6 months prior to screening may be included in the study. Subjects with a positive urine drug screen due to prescription medication (e.g., opiates, methylphenidate) are eligible if the prescription and diagnosis are reviewed and approved by the investigator.
22. Concurrent participation in another therapeutic clinical study.
23. Presence of any laboratory abnormality or significant systemic or major illnesses (other than liver disease) that, in the opinion of the Investigator, compromise the subject’s ability to safely participate in and complete the study including:
    a. Pulmonary disease, renal failure, organ transplantation, serious psychiatric disease, malignancy, history of substance abuse and/or a psychiatric condition requiring hospitalization and/or emergency room visit within six months of screening.
24. Unavailable for follow-up assessment or concern for subject’s compliance with the protocol procedures.
25. History of significant pancreatic disorders (acute pancreatitis, chronic pancreatitis, hereditary pancreatitis, and pancreatic cancer).

5. INVESTIGATIONAL MEDICINAL PRODUCTS (IMP)

5.1. Randomization, Blinding and Treatment Codes

An Interactive Response Technology (IRT) will be used for centralized randomization and treatment assignment. Randomization will be stratified by baseline hepatic fat fraction (< 15% vs ≥ 15%) and F1 fibrosis score in the Main Study.

There will be no stratification for Cohort C.

Investigative site personnel will obtain the treatment assignment from the IRT. Investigational medicinal product will be prepared by an unblinded, qualified health care professional (e.g., pharmacist or designee) at the investigative site who is not otherwise associated with the study. Investigators, study site personnel, subjects and the Sponsor and/or designee will be blinded to treatment assignments. Investigational medicinal products will be dispensed in a blinded fashion to the subjects.

5.1.1. Procedures for Breaking Treatment Codes

In the event of a medical emergency where breaking the blind is required to provide medical care to the subject, the investigator may obtain treatment assignment directly from the IRT for that subject. Akero recommends but does not require that the investigator contact the Medpace medical monitor before breaking the blind. Treatment assignment should remain blinded unless that knowledge is necessary to determine subject emergency medical care. The rationale for unblinding must be clearly explained in source documentation and on the electronic case report form (eCRF), along with the date on which the treatment assignment was obtained. The investigator is requested to contact the Medpace medical monitor promptly in case of any treatment unblinding.

Blinding of study treatment is critical to the integrity of this clinical trial and therefore, if a subject's treatment assignment is disclosed to the investigator, the subject will have study treatment discontinued. All subjects will be followed until study completion unless consent to do so is specifically withdrawn by the subject.

The CRO's pharmacovigilance and/or designee may independently unblind cases for expedited reporting of suspected unexpected serious adverse reactions (SUSARs).

5.2. Investigational Product(s)

EFX is provided in 6 mL sterile vials filled with a 1.1 mL volume at a strength of 70 mg/mL. EFX will be presented as a sterile, colorless to slightly yellow and preservative-free frozen liquid.

Placebo will be presented in identical containers and packaged the same as EFX.

For preparation of the 28 mg and 50 mg doses, diluent vials will be provided. Refer to the Pharmacy Manual for further instructions on EFX/placebo administration and use of the diluent vials.

The Diluent will be presented as a sterile, clear, colorless refrigerated liquid in an identical vial as EFX. The 6 mL vials supplied will contain approximately 3.6 mL of diluent and each vial is intended for single use only.

Investigational medicinal products are to be distributed to centers in the US are labeled to meet applicable requirements of the United States Food and Drug Administration (FDA).

5.2.1. Storage and Handling

EFX / Placebo will arrive frozen in secondary box packaging and should be immediately placed in a freezer maintained between -30°C to -70°C until planned use. The EFX / Placebo vials should be stored protected from light in a secure non-frost-free freezer prior to thawing and use. The set point for the freezer should be between -30°C to -70°C. The set point is a single temperature and should remain constant. Do not refreeze EFX / Placebo after it has been thawed.

Freezer Set Point (°C) for EFX / Placebo	Acceptable Parameters:	Acceptable Range:
-30°C to -70°C	-30°C (± 10°C) to -70°C (± 10°C)	-20°C to -80°C

The Diluent vials are shipped in refrigerated containers maintained at a range of 2°C to 8°C and must not be frozen. The Diluent vials should be stored in a secure refrigerator that is maintained between 2°C to 8°C.

All vials of IMP should be stored in a securely locked area, accessible only to authorized site personnel.

Records of the actual storage conditions during the period of the study must be maintained (e.g., records of the date and time and initials of person checking, and the "working day" temperatures of the refrigerator and freezer used for storage of study supplies, continuous temperature recordings, or regularly maintained temperature alarm systems used in conjunction with temperature recording).

Akero must be notified if any IMP undergoes temperature excursions or if the IMP is damaged. IMP supply should not be utilized unless Akero personnel have advised that it is acceptable to do so.

5.2.2. Dosage and Administration

Detailed information regarding the preparation and administration of EFX or Placebo for this study is contained in the EFX Pharmacy Manual. Investigational medicinal product will be administered by subcutaneous injection once weekly as follows:

Main Study:

- Placebo QW for 16 weeks (n = 20)
- 28 mg EFX QW for 16 weeks (n = 20)
- 50 mg EFX QW for 16 weeks (n = 20)
- 70 mg EFX QW for 16 weeks (n = 20)

Cohort C:

- Placebo QW for 16 weeks (n = 10)
- 50 mg EFX QW for 16 weeks (n = 20)

The IMP will be administered by a weekly 1.0 mL subcutaneous injection into the abdomen at the clinic by a qualified staff member. The date and time of injection will be recorded. If injection site reactions are observed, mitigation procedures should be put in place as per local standard of care. Examples of mitigations include icing the injection site, rotating the quadrant of the abdomen in which the injection is administered, or providing antihistamine prior to injection.

5.3. Prior and Concomitant Medications

All concomitant medication will be recorded in the source documents and eCRFs. This includes concomitant medications taken within 30 days prior to screening and any taken during the study through end of the study.

Use of any investigational medication within 30 days or within 5 half-lives prior to screening and throughout the study is prohibited.

Subjects with a history of (12 months prior to screening) or current use of prescription drugs associated with liver steatosis (e.g. methotrexate, amiodarone, high-dose estrogen, tamoxifen, systemic steroids, anabolic steroids, valproic acid) should be excluded. Short courses of corticosteroids (less than two weeks) may be allowed prior to screening if in the investigator's opinion they are not associated with liver steatosis or clinically relevant.

As EFX is expected to improve insulin sensitivity, subjects on Sulfonylureas with unstable blood glucose levels should be reminded of the symptoms of hypo- and hyper-glycemia and how to correct them.

Main Study:

Subjects on Vitamin E ≥ 400 IU/day, thiazolidinediones (including, but not limited to, pioglitazone, rosiglitazone, and lobeglitazone), GLP1, or SGLT2 must be on a stable dose (defined as no changes in prescribed dose, new medications, or discontinuation) for at least 6 months prior to the diagnostic liver biopsy and screening.

Subjects who have insulin dependent Type 2 diabetes are excluded from the study. Subjects on other antidiabetic, weight loss, or lipid-modifying medication(s) must be on stable dose(s) for at least 3 months prior to the diagnostic liver biopsy through randomization. Dose adjustments for metformin are allowed in the 3 months prior to screening. If possible, the doses of these medications should remain stable through the end of treatment. Fibrates and peroxisome proliferator-activated receptor (PPAR) alpha agents (clofibrate, gemfibrozil, ciprofibrate, bezafibrate, or fenofibrate) are prohibited from screening until the end of the study.

Cohort C:

Subjects on Vitamin E ≥ 400 IU/day, thiazolidinediones (including, but not limited to, pioglitazone, rosiglitazone, and lobeglitazone), GLP1, or SGLT2 must be on a stable dose (defined as no changes in prescribed dose, new medications, or discontinuation) for at least 6 months prior to the screening visit.

Subjects who have insulin dependent Type 2 diabetes are excluded from the study. Subjects on other antidiabetic, weight loss, or lipid-modifying medication(s) must be on stable dose(s) for at least 3 months prior to screening. Dose adjustments for metformin are allowed in the 3 months prior to screening. If possible, the doses of these medications should remain stable through the end of treatment. Fibrates and peroxisome proliferator-activated receptor (PPAR) alpha agents (clofibrate, gemfibrozil, ciprofibrate, bezafibrate, or fenofibrate) are prohibited from screening until the end of the study.

5.4. Investigational Medicinal Product Accountability

The Sponsor or designee will supply the Investigational Medicinal Product (IMP). The site will maintain the following records: receipt of shipments, dispensation to subjects, and return of partially used, or unused IMP.

5.4.1. Investigational Medicinal Product Return or Disposal

At the start of the study, the study monitor will evaluate the study center's study drug disposal procedures and provide appropriate instruction for return or destruction of unused IMP supplies. If the site has an appropriate Standard Operating Procedure (SOP) for drug destruction, the site may destroy used (empty bottles) and unused IMP supplies performed in accordance with the site's (hospital/pharmacy) SOP. If the site does not have acceptable procedures in place for drug destruction, arrangements will be made between the site and Akero (or Akero's representative) for return of unused IMP supplies. A copy of the site's SOP will be obtained for central files. Where possible, IMP will be destroyed at the site. Upon study completion, a copy of the Investigational Drug Accountability records must be filed at the site. Another copy will be returned to Akero. If IMP is destroyed on site, the Investigator must maintain accurate records for all IMP vials destroyed. Records must show the identification and quantity of each unit destroyed, the method of destruction, and person who disposed of the IMP. All IMP records must be maintained at the site and copies must be submitted to Akero at the end of the study.

6. STUDY PROCEDURES

The following assessments will be conducted during screening and at selected time points specified in the Schedule of Assessments (Appendix A and Appendix B). Additional procedures deemed necessary as part of standard of care may be performed at the discretion of the Investigator. All missed visits and any performed procedures that are not protocol-specified activities must be documented in the subject's medical record and the appropriate eCRF.

6.1. Screening and Informed Consent

Before subjects commence any study-specific activities or procedures, the Sponsor requires a copy of the study site's written institutional review board (IRB) approval of the protocol, informed consent form (ICF), and all other subject information and/or recruitment material, if applicable. Each subject (or legally acceptable representative) must sign and date the ICF before participating in study-specific activities.

After the ICF is signed, the subject enters the screening period. Subjects will be screened within 6 weeks before randomization to determine eligibility for participation in the study. The screening period may be extended to 8 weeks to allow for pathology assessment of the liver biopsy. Subjects who are ineligible at the screening visit may be re-screened if there is reasonable belief that the exclusionary result was obtained in error or is transient upon approval of the Medical Monitor. Subjects who screen fail for the Main Study due to cirrhosis may apply any assessments from the original screening visit that are still within window for potential enrollment into Cohort C.

After completing the screening period, the subject will be evaluated by the investigator to confirm eligibility. A subject is considered enrolled when the investigator decides that all of the eligibility criteria are met. The investigator will document this decision and date and time in the subject's medical record and the eCRF. Screen failure subjects will be entered into the eCRF. Investigators will maintain a screening log of all potential subjects that includes limited information about each subject, dates, and outcome of screening process (e.g., enrolled into study, reason for ineligibility, or withdrawal of consent).

6.2. Demographics and Medical History

Demographic data including sex, age, race, and ethnicity will be collected.

The Investigator or designee will collect medical and surgical history that started prior to enrollment through the time of consent, including information on the subject's concurrent medical conditions. All findings will be recorded on the medical history eCRF.

6.3. Physical Examination

The Investigator or designee will conduct a complete physical examination, or a symptom-driven exam as outlined in the Schedule of Assessments. Physical exam findings prior to the

first dose of study drug will be recorded on the medical history eCRF page and clinically significant findings after the first dose of study drug will be recorded as AEs.

At a minimum, the complete physical examination should include assessments of the head and neck, skin, nervous system, lungs, cardiovascular system, abdomen, thyroid, lymph nodes, and extremities.

A symptom-driven physical examination will include assessment of any new subject complaints or changes from baseline.

Physical assessments required to calculate Child-Pugh Score (CP Score) for Cirrhosis Mortality to be completed as part of the physical examination as indicated in the Schedule of Assessments.

6.4. Vital Signs, Weight, Hip and Waist Circumference

The following vital sign measurements will be performed as outlined in the schedule of assessments: systolic and diastolic blood pressure, pulse, respiration rate, oral temperature, height, weight, hip and waist circumference.

The subject must be in seated or in a semi-recumbent position in a rested and calm state for at least 5 minutes before vital signs are collected. The position selected for a subject should be the same throughout the study and documented on the vital signs eCRF. Triplicate blood pressure (to be measured in same arm with at least two minutes rest between BP measurements) and heart rate to be collected at baseline and between 48-96 hours post-dose (to correspond to C_{max}) at the timepoints indicated in the schedule of assessments.

Height will be measured without shoes at screening.

Weight will be obtained at screening and selected time points thereafter. On dosing days, weight will be collected pre-dose. Weight should be obtained using a calibrated scale. The subject should be weighed in consistent clothing.

Waist circumference should be measured by placing the tape measure around the waist just above the top of the hip bone. Ensure it is snug against the skin, but not tight.

Hip circumference should be measured by placing the tape measure around the widest part of the buttocks. Ensure the tape measure is parallel to the floor, and not slanted.

Weight, waist and hip circumference should be obtained by the same study personnel between visits for each subject to minimize variability in the measurement.

6.5. 12-Lead Electrocardiography

The subject must be in a seated, semi-recumbent, or supine position in a rested and calm state for at least 10 minutes before ECG assessment is conducted. Each 12-lead ECG should be performed prior to blood draws, dosing (if applicable), or other invasive procedures. Each

ECG must capture QRS, QT, QT interval corrected for heart rate (QTc), RR, and PR intervals and be documented on the ECG eCRF.

The Investigator or designated study site physician will review, sign, and date all ECGs. The original ECGs will be retained with the subject's source documents. At the request of the Sponsor, a copy of the original ECG will be made available to the Sponsor.

6.6. FibroScan®

FibroScan® examinations will be performed and median liver stiffness in kilopascals (kPa), interquartile range/median value (IQR/M), and success rate (number of valid shots/total number of shots) will be recorded. Where available, the median CAP and the interquartile range of CAP values will be recorded from FibroScan® examinations. For the Main Study, a FibroScan® measurement of greater than 7.0 kPa is required to continue to progress the subject through the remaining screening assessments.

At least 2-3 hours fasting is required prior to all elastography assessments.

6.7. Liver Biopsy

Main Study:

A liver biopsy must be obtained at screening and between weeks 22-24 (for subjects with ≥ 30% relative fat reduction on MRI-PDFF at Week 12) to provide the liver tissue for central reading. All reasonable attempts should be made to acquire a liver biopsy specimen of at least 2.0 cm in length. It is recommended that a 16-gauge needle is used to collect the tissue sample.

A historical biopsy that meets eligibility criteria may be accepted as the screening biopsy if the sample is deemed acceptable for interpretation by the central reader. The historical sample must have been originally obtained within 180 days of the Randomization date and confirm the below criteria:

- Subject was not previously treated for NASH/NAFLD with prohibited concomitant medications (including investigational products) within 6 months of historical biopsy.
- Subject was not previously treated with an Investigational Product within 6 months prior to or at the time of historical biopsy.

If the historical biopsy is not adequate for interpretation by the central reader, or was obtained more than 180 days prior to randomization, a fresh liver biopsy will be collected. Liver biopsies will be sent to a central laboratory and then read by a central reader. The central reader will read all screening biopsies for eligibility.

Cohort C:

A historical biopsy performed within 24 months of the Screening Visit or for those subjects who undergo a liver biopsy for eligibility, the following is required for enrollment:

- Biopsy must be consistent with NASH and cirrhosis as assessed by a local pathologist.
- Subject was not previously treated for NASH/NAFLD (including investigational products unless documentation can be provided that the subject was randomized to placebo) between obtaining the historical biopsy and the Screening visit.

Following 16 weeks of treatment, subjects will be given the option to collect an end of treatment biopsy. Both the historical biopsy sample used to qualify the subject at screening as well as the end of treatment biopsy sample will be shipped for blinded pathology analysis by the central reader.

All Subjects:

If a liver biopsy is performed per standard of care outside of protocol mandated assessments, all possible attempts should be made to submit the biopsy specimen to the central reader for evaluation.

If liver biopsy results are deemed unevaluable by the central reader, additional slides may be provided, or a repeat biopsy may be performed at the discretion of the Investigator.

6.8. MRI-PDFF

Main Study Only:

The degree of steatosis will be measured by MRI-PDFF. Subjects who have contraindications to MR imaging (e.g., unmanageable claustrophobia or metal implants) should be excluded from the study.

The MRI-PDFF images will be analyzed by a central reader. Please refer to the Imaging Manual for MRI-PDFF imaging guidelines. At least 4 hours fasting is required prior to all MR assessments.

Corrected T1 (cT1) will also be collected at the same time as MRI-PDFF, under the same conditions, where available.

6.9. DXA Scan

A DXA scan will be performed to confirm eligibility and again after treatment has been discontinued. DXA scan should include lumbar spine, femoral neck, and total hip with subjects in the standard (supine) position.

Total body composition may also be collected at the DXA timepoints, where available, to determine body fat distribution.

Refer to the DXA manual for more information.

6.10. Health Related Quality of Life Questionnaires

It is recommended that the questionnaires be completed prior to any study procedures being performed and prior to the subject seeing a health care provider. If the baseline questionnaires are missed at Day 1, questionnaires should not be collected at the remaining timepoints.

6.10.1. Short Form 36 Health Survey (SF-36)

The SF-36 asks 36 questions to measure functional health and well-being from the subject's point of view. It consists of eight health domains: physical functioning, role-physical, bodily pain, general health, vitality, social functioning, role-emotional, and mental health. These health domain scales contribute to the physical health and mental health summary measures.

6.10.2. Chronic Liver Disease Questionnaire-Nonalcoholic Fatty Liver Disease (CLDQ-NAFLD)

The CLDQ-NAFLD asks questions related to liver disease and specifically NAFLD, to measure health related quality of life in subjects with chronic liver disease.

6.10.3. Work Productivity and Activity Impairment (WPAI)

The WPAI questionnaire asks questions regarding the effect of NASH on a person's ability to work and perform regular activities.

6.11. Alcohol Use Disorders Identification Test (AUDIT-C)

The Alcohol Use Disorders Identification Test (AUDIT-C) is a 3-item screening tool developed by the World Health Organization (WHO) to assess alcohol consumption, drinking behaviors, and alcohol-related problems. Subjects should be encouraged to answer the AUDIT-C questions in terms of standard drinks. A chart illustrating the approximate number of standard drinks in different alcohol beverages is included for reference.

6.12. Lifestyle Modification

Lifestyle modifications such as weight loss via diet and increased exercise can be effective in the treatment of NASH. All subjects will receive counseling regarding lifestyle modifications including the maintenance of a healthy diet and participation in regular exercise at the Baseline visit. At each subsequent visit, subjects will be asked if there has been a significant change in diet, or physical activity. Changes will be documented in the eCRF.

6.13. Clinical Laboratory Tests

6.13.1. Laboratory Parameters

Blood samples will be collected according to the Schedule of Assessments (Appendix A and Appendix B) and below. Samples will be sent to the central laboratory for analysis and reporting. Samples may be analyzed for the tests outlined in this protocol and for any additional tests necessary to ensure subject safety. These may include, but are not limited to, investigation of unexpected results and incurred sample reanalysis.

At visits where CTX-1 or lipids are collected, the samples should be collected in the morning following an overnight fast of at least 8 hours.

Subjects will be in a seated, semi-recumbent, or supine position during blood collection. Clinical laboratory tests will include the following:

Table 3. Clinical Laboratory Assessments

Chemistry	Hematology/Coagulation	Urine/Miscellaneous	Biomarkers
Sodium	**Hematology**	**Urine**	**Fibrosis Biomarkers**
Potassium	RBC count	Drug screening:	ELF
Chloride	Hemoglobin (Hgb)	amphetamines,	Pro-C3
Bicarbonate	Hematocrit (Hct)	cocaine and opiates	
Calcium	Platelet count	(i.e., fentanyl, heroin,	**Bone Biomarkers**
Magnesium		morphine)	Osteocalcin
Phosphorus	WBC count with		P1NP
Glucose[b]	differential: neutrophils,	**Miscellaneous**	CTX-1[b]
BUN	lymphocytes, monocytes,	Screening Serology	BSAP
Creatinine	eosinophils, and basophils	Tests	Parathyroid hormone
Total protein		HBsAg	(PTH)
Albumin	ANC	HBcAb	Vitamin D
Uric acid		HBV DNA	
LDH	**Coagulation**	Anti-HCV antibody	**Lipid Metabolism**
Total (TBL) and	PT	HCV RNA	**Biomarkers**
direct bilirubin	aPTT	HIV	Lp (a)
Alkaline phosphatase	INR		ApoB, ApoC2 & Apo
(ALP)	PAI-1	Endogenous FGF21[b]	C3
ALT (SGPT)	Fibrinogen		3 Hydroxy-butyrate
AST (SGOT)		Saliva Cortisol	Bile acids
GGT			
CK		Pregnancy test[a]	**Insulin Sensitivity &**
hsCRP			**Glycemic Control**
Amylase		Pharmacokinetics	**Biomarkers**
Lipase[c]		(PK)	Insulin
			Glucagon
**Lipoproteins**		Anti EFX antibody	Adiponectin
Total Cholesterol		(ADA)	C-Peptide
HDL-C[b]		Neutralizing Anti EFX	HOMA-IR
LDL-C[b]		antibody (NAB)	HbA1c
Triglycerides			
Free Fatty Acids[b]			

ADA = anti-drug antibodies; ALT = alanine aminotransferase; ANC = absolute neutrophil count; Apo = Apolipoprotein; AST = aspartate aminotransferase; aPTT = activated partial thromboplastin time; BSAP= bone specific alkaline phosphatase; BUN = blood urea nitrogen; ELF = Enhanced Liver Fibrosis panel; GGT = gamma-glutamyl transferase; HbA1c = Glycated Hemoglobin A1c; HBcAb = total hepatitis B core antibody; HBsAg = hepatitis B surface antigen; HBV DNA = hepatitis B virus deoxyribonucleic acid; HCV = hepatitis C virus; HDL-C = high density lipoprotein cholesterol; HIV = human immunodeficiency virus; HOMA-IR = Homeostatic Model Assessment of Insulin Resistance; hsCRP = high sensitivity C-Reactive Protein; INR = International Normalized Ratio; LDH = lactate dehydrogenase; LDL-C = low density lipoprotein cholesterol; Lp = Lipoprotein; L&HMW = low and high molecular weight; PAI-1 = Plasminogen Activator Inhibitor-1; Pro-C3 = N-terminal Type III Collagen Propeptide; PT =prothrombin time; RBC = red blood cell; RNA = ribonucleic acid; TBL, total bilirubin; WBC = white blood cell

[a] For female subjects of childbearing potential only. Serum pregnancy test at screening and urine pregnancy test at other time points.

[b] Samples to be collected in the morning following an overnight fast of at least 8 hours.

[c] Reflex lipase testing is performed in subjects with total amylase > 1.5 ULN.

6.14. Sample Collection, Storage, and Shipping

Blood and urine samples from screening through ET or end of study visits will be collected and submitted to the central laboratory for analysis. Validated assays will be used for analysis when appropriate.

6.15. Pharmacokinetic Assessments

Pharmacokinetic samples will be collected at time points specified in the Schedule of Assessments (Appendix A and Appendix B).

6.16. Anti-Drug Antibody and Neutralizing Antibody Sampling

Samples will be obtained for detection of anti-drug antibodies (ADA) against EFX as outlined in the Schedule of Assessments (Appendix A and Appendix B).

In the event of a positive anti-EFX antibody detection, a neutralizing ADA assay will be run for the sample timepoint with the positive result.

Subjects who test positive for binding antibodies to EFX at the final scheduled study visit will be required to return for additional follow-up testing. This testing is to occur once the site has been notified of the positive result and approximately every 3 months until: (1) antibodies are no longer detectable or (2) the subject has been followed for a period of at least 6 months (± 2 weeks) after administration of EFX. All follow-up results, both positive and negative, will be communicated to the sites. More frequent testing (e.g., every month) or testing for a longer period of time may be requested in the event of safety-related concerns. Follow-up testing is not required where it is established that the subject did not receive EFX.

6.17. Blood Storage

A portion of the blood samples drawn at study visits will be frozen and stored. The stored samples may be used for future clinical laboratory testing to provide additional clinical data. No human genetic testing will be performed without expressed consent of study subjects. At the conclusion of the study, these samples may be retained in storage by Akero for a period of up to 15 years.

6.18. Biomarkers

The biological specimens collected in this study will be used to evaluate the association of exploratory systemic biomarkers with study drug response, including efficacy and/or AEs and to increase knowledge and understanding of the biology of how EFX affects subjects with NASH. Biological specimens may also be used to increase the knowledge of diseases such as NAFLD, liver fibrosis, inflammatory diseases, bone turnover, and/or the validation of a companion diagnostic if applicable. The specific analyses will include, but will not be limited to, the biomarkers and assays described in Table 3. As biomarker science is a rapidly evolving area of investigation, and AEs in particular are difficult to predict, it is not possible to specify prospectively all tests that will be performed on the specimens provided. The

testing outlined is based upon the current state of scientific knowledge and may be modified during or after the end of the study to remove tests no longer indicated and/or to add new tests based upon the newest technologies.

Samples will be destroyed no later than 15 years after the end of the study. The specimen storage period will be in accordance with the IRB/independent ethics committee (IEC)/ethics committee (EC) approved ICF and applicable laws (e.g., health authority requirements).

6.19. Criteria for Discontinuation of Study Drug

Study drug must be discontinued in the following instances:

- Liver transplantation
- Subject develops an SAE consisting of a serious hypersensitivity reaction to study drug
- Subject experiences diarrhea that meets the criteria in Section 6.23 for discontinuation.
- Subject experiences a Grade IV Common Terminology Criteria for Adverse Events (CTCAE) or meets the criteria for Drug Induced Liver Injury described in Section 6.24.
    - The Data Monitoring Committee (DMC) should perform a causality assessment. The study drug may be restarted if the DMC concludes that the AE or laboratory abnormalities were not related to study drug, the AE has resolved, laboratory abnormalities have returned to baseline, and the subject is amenable to close clinical follow-up.
    - If, after re-challenge, a subject has a second serious AE or recurrent elevations (even mild or minimal) of total bilirubin, ALT, or AST as defined in Section 6.24.3, study drug should be discontinued permanently.
- Intercurrent illness that would, in the judgment of the Investigator, affect assessments of clinical status to a significant degree. Following resolution of intercurrent illness, the subject may resume study dosing at the discretion of the Investigator, in consultation with the Medical Monitor
- Unacceptable toxicity or toxicity that, in the judgment of the Investigator, compromises the ability to continue study-specific procedures or is considered to not be in the subject's best interest
- Subject or Investigator request to discontinue for any reason
- Significant subject noncompliance
- Significant protocol violation that impacts subject safety

- Pregnancy during the study; refer to Appendix C.

- Discontinuation of the study at the request of Akero, a regulatory agency, or an IRB/IEC/EC.

Cohort C

In addition to the above criteria, study drug must be discontinued in the following instances in Cohort C:

- Development of any decompensation events including ascites, hepatic encephalopathy (HE), or variceal bleeding

- Progression to Child Pugh Class C

6.20. Interruption of Study Drug

If dosing is interrupted (i.e., as a result of an AE), every attempt should be made to keep the subject in the study and continue to perform the required study-related procedures. Discussion with the Medical Monitor is recommended. If this is not possible or acceptable to the subject or Investigator, the subject may be withdrawn from the study.

6.21. Adverse Events

An AE is any unfavorable and unintended sign (including an abnormal laboratory finding), symptom, or disease temporally associated with the use of a study drug, whether or not related to the study drug. AEs may also include pre- or post-treatment complications that occur as a result of protocol specified procedures, lack of efficacy, overdose, drug abuse/misuse reports, or occupational exposure. Pre-existing events that increase in severity or change in nature during or as a consequence of participation in the clinical study will also be considered AEs. AEs that occur after the first dose of EFX or during the study treatment and safety follow up periods will be documented on the AE eCRF. The Investigator will assess the AE severity and relationship of the AE to study drug. The Investigator will treat the subject as medically required.

Laboratory values that are outside the laboratory reference range should be reported as AEs only if considered clinically significant by the Investigator.

From the time of obtaining informed consent until 30 days after the last administration of study drug, all SAEs and non-serious AEs related to protocol-mandated procedures will be recorded on the SAE/AE eCRF. All other untoward medical occurrences observed during screening, including exacerbation or changes in medical history, will be captured on the medical history eCRF. Details on recording and reporting AEs are provided below.

Clinical judgment of the Investigator should be used to determine whether a subject is to be withdrawn due to an AE. In the event the subject requests to withdraw from study-related

treatment or the study due to an AE, the subject should be followed for the safety follow up period as outlined in the Schedule of Assessments.

All subjects experiencing AEs, including clinically significant abnormal laboratory values, whether or not associated with use of the study drug, must be monitored until the condition returns to normal, returns to the subject's baseline, until the Investigator determines the AE has reached a stable outcome and is no longer clinically significant, or the subject is considered lost to follow up.

6.21.1. Severity

All AEs, both serious and non-serious, will be assessed for severity using the Common Terminology Criteria for Adverse Events (CTCAE) Version 5.0. The CTCAE scale includes unique clinical descriptions of AEs that are categorized by anatomy and/or pathophysiology and is provided in the Site Operations Manual.

The CTCAE scale displays Grades 1 through 5 with unique clinical descriptions of severity for each AE (including abnormal laboratory values) based on this general guideline provided in the CTCAE scale. For any AEs not covered by CTCAE the conventional definition of severity will be used.

- Grade 1 (Mild) AE: minor; no specific medical intervention; marginal clinical relevance
- Grade 2 (Moderate) AE: minimal intervention; local intervention; noninvasive intervention
- Grade 3 (Severe) AE: significant symptoms requiring hospitalization or invasive intervention;
- Grade 4 (Life-threatening or disabling) AE: Complicated by acute, life-threatening complications; need for intensive care or emergent invasive procedure
- Grade 5 Fatal AE

6.21.2. Relationship

The Investigator or qualified sub-Investigator is responsible for assessing the relationship to study drug using clinical judgment and the following considerations:

- Not related: all AEs should be considered related unless the AE is clearly unrelated to study drug or clearly due to the subject's underlying disease. For a SAE, an alternative causality must be provided (e.g., preexisting condition, underlying disease, intercurrent illness, or concomitant medication).
- Related: There is reasonable possibility that the event may have been caused by the study drug.

Ineffective treatment should not be reported as an AE.

The relationship to study-related procedures (e.g., invasive procedures such as venipuncture or biopsy) should be assessed using the following considerations:

- Not related: Evidence exists that the AE has an etiology other than the study procedure.
- Related: The AE occurred as a result of study procedures.

6.21.3. Overdose

An overdose is any dose of study drug given to a subject that exceeds the protocol specified dose by 10% or more. In the event of an overdose-associated AE, appropriate supportive therapy should be initiated according to the subject's signs and symptoms.

Any overdose, with or without associated AEs, will be promptly reported to the Sponsor and recorded as non-compliance on the eCRF. AEs associated with overdose should be reported on relevant AE/SAE sections in the eCRF.

6.21.4. Serious Adverse Events

6.21.4.1. Definition

A SAE is defined by federal regulation as any AE occurring at any dose that results in any of the following outcomes: death, life-threatening AE, hospitalization or prolongation of existing hospitalization, a persistent or significant incapacity or substantial disruption of the ability to conduct normal life functions, or a congenital anomaly/birth defect.

Important medical events that may not result in death, be life threatening, or require hospitalization may be considered an SAE when, based upon appropriate medical judgment, they may jeopardize the subject, and may require medical or surgical intervention to prevent one of the outcomes listed in this definition. Examples of such medical events include allergic bronchospasm requiring intensive treatment in an emergency room or at home, blood dyscrasias or convulsions that do not result in inpatient hospitalization, or the development of drug dependency or drug abuse.

A life-threatening AE is one that, in the view of the Investigator, places the subject at immediate risk of death from the reaction as it occurred.

An unexpected AE is any event for which the specificity or severity is not consistent with the AE profile in the current EFX IB.

All SAEs, regardless of cause(s) or relationship to study drug, must be reported immediately to the study Sponsor and/or designee.

6.21.5. Reporting Adverse Events

6.21.5.1. Reporting Procedures for Non-Serious Adverse Events

The Investigator is responsible for ensuring that all AEs observed by the Investigator or designee or reported by the subject are reported using the AE eCRF.

The Investigator will assign the following AE attributes:

- AE diagnosis or syndrome(s), if known (if not known, signs or symptoms)
- Dates of onset and resolution (if resolved)
- Severity
- Relatedness to study drug or study-related procedures
- Action taken

Follow-up of non-serious AEs will continue through the last day on the study and/or until a definitive outcome (e.g., resolved, resolved with sequelae, lost to follow-up) is achieved.

When a subject is withdrawn from the study because of a non-serious AE, the Sponsor and/or designee must be notified by e-mail or phone within 48 hours.

6.21.5.2. Reporting Procedures Serious Adverse Events

The Investigator is responsible for ensuring that all SAEs observed by the Investigator or reported by the subject are promptly assessed and reported. The Investigator must assess whether the SAE is related to study drug or any study-related procedure.

The procedures for reporting SAEs are as follows:

Initial Reports

- All SAEs occurring after the first dose of EFX or during the study treatment and safety follow up periods must be reported to Medpace Clinical Safety within 24 hours of the knowledge of the occurrence. After the protocol-specified reporting window, any SAE that the investigator considers related to study must be reported to Medpace Clinical Safety or the Sponsor/designee.

- To report the SAE, complete the SAE form electronically in the electronic data capture (EDC) system for the study. When the form is completed, Medpace Safety personnel will be notified electronically by the EDC system and will retrieve the form. If the event meets serious criteria and it is not possible to access the EDC system, send an email to Medpace Safety at medpace-safetynotification@medpace.com or call the Medpace SAE hotline (phone number listed below), and fax/email the completed paper SAE form to Medpace (contact information listed below) within 24 hours of awareness. When the EDC system becomes available, the SAE information must be entered within 24 hours of the system becoming available.

Follow-Up Reports

- The investigator must continue to follow the subject until the SAE has subsided or until the condition becomes chronic in nature, stabilizes (in the case of persistent impairment) or the subject dies.

- Within 24 hours of receipt of follow-up information, the investigator must update the SAE form electronically in the EDC system for the study, and submit any supporting documentation (e.g., subject discharge summary or autopsy reports) to Medpace

Clinical Safety via fax or e-mail. If it is not possible to access the EDC system, refer to the procedures outlined above for initial reporting of SAEs.

- The Sponsor or/or designee may request additional information from the Investigator to ensure the timely completion of accurate safety reports.

The Investigator must take all therapeutic measures necessary for resolution of the SAE. Any medications or therapies necessary for treatment of the SAE must be recorded in the SAE form and the concomitant medication eCRF.

Follow-up of SAEs will continue through the last day on the study and/or until a definitive outcome (e.g., resolved, resolved with sequelae, lost to follow-up, fatal) is achieved.

If a subject becomes pregnant during the study or within the safety follow up period defined in the protocol, the investigator is to stop dosing with study drug(s) immediately and the subject should be withdrawn from the study. Early termination procedures and safety follow-up should be implemented at that time.

A pregnancy is not considered to be an AE or SAE; however, it must be reported to Medpace Clinical Safety within 24 hours of knowledge of the event. Medpace Clinical Safety will then provide the investigator/site the Exposure In Utero (EIU) form for completion. The investigator/site must complete the EIU form and fax/email it back to Medpace Clinical Safety.

If the female partner of a male subject becomes pregnant while the subject is receiving study drug or within the safety follow up period defined in the protocol, the investigator should notify Medpace Clinical Safety as described above.

The pregnancy should be followed until the outcome of the pregnancy, whenever possible. Once the outcome of the pregnancy is known, the follow-up EIU form should be completed and faxed/emailed to Medpace Clinical Safety. If the outcome of the pregnancy meets the criteria for immediate classification as an SAE (i.e., postpartum complication, spontaneous abortion, stillbirth, neonatal death, or congenital anomaly), the investigator should follow the procedures for reporting an SAE.

Safety Contact Information:
Medpace Clinical Safety

Medpace SAE hotline – USA:
Telephone: +1-800-730-5779, option “3” **or** +1-513-579-9911, option “3”
Facsimile: +1-866-336-5320 **or** +1-1-513-570-5196

E-mail: medpace-safetynotification@medpace.com

Some SAEs will qualify for reporting to the Food and Drug Administration (FDA) as applicable via the MedWatch/CIOMS reporting system in accordance with FDA and other

applicable regulations. The Sponsor or its designee will report SAEs and/or SUSARs as required to regulatory authorities and Investigators in compliance with all reporting requirements according to local regulations and Good Clinical Practice (GCP).

The Investigator will notify the appropriate IRB of SAEs occurring at the study site and other AE reports received from the Sponsor, in accordance with local procedures and statutes. The Investigator or designee at each study site is responsible for submitting Investigational New Drug (IND) safety reports (initial and follow-up) and other safety information (e.g., revised Investigator's Brochure) to the IRB and for retaining a copy in the study files.

6.22. Adverse Events of Special Interest

The Investigator will monitor each subject for clinical and laboratory-evidence for predefined AEs of special interest (AESIs) throughout the subject's participation in this study. The Investigator should treat the AESI according to local standard of care. The following conditions will be monitored as AESIs during this study:

- Injection site reactions
- Diarrhea
- Hypoglycemia

In addition, drug-induced liver injury will be monitored and managed according to Section 6.24.

During the course of the study, additional AESIs may be identified by the Sponsor. The Investigator will assess and record in detail any additional information for AESIs on the AE eCRF form.

6.23. Clinical Laboratory Abnormalities and Other Abnormal Assessments as Adverse Events or Serious Events

Laboratory abnormalities are usually not recorded as AEs or SAEs, however, laboratory abnormalities (e.g., clinical chemistry, hematology, urinalysis) independent of the underlying medical condition that require medical or surgical intervention or lead to investigational medicinal product interruption or discontinuation must be recorded as an AE, as well as an SAE, if applicable. In addition, laboratory or other abnormal assessments (e.g., electrocardiogram, X-rays, vital signs) that are associated with signs and/or symptoms must be recorded as an AE or SAE if they meet the definition of an AE (or SAE) as described in Section 6.21. If the laboratory abnormality is part of a syndrome, record the syndrome or diagnosis (e.g., anemia) not the laboratory result (i.e., decreased hemoglobin).
Severity should be recorded and graded according to the CTCAE Version 5.0. For AEs associated with laboratory abnormalities, the event should be graded based on the clinical severity in the context of the underlying conditions; this may or may not be in agreement with the grading of the laboratory abnormality.

Toxicity Management
Any questions regarding toxicity management should be directed to the Medical Monitor.

Grade 1 and 2 Laboratory Abnormality or Clinical Event
Continue investigational medicinal product at the discretion of the Investigator.

Grade 3 Laboratory Abnormality or Clinical Event

- For Grade 3 clinically significant laboratory abnormality or clinical event, investigational medicinal product may be continued if the event is considered to be unrelated to investigational medicinal product.

- For a Grade 3 clinical event, or clinically significant laboratory abnormality confirmed by repeat testing, that is considered to be related to investigational medicinal product, investigational medicinal product should be withheld until the toxicity returns to ≤ Grade 2.

- If a laboratory abnormality recurs to ≥ Grade 3 following re-challenge with investigational medicinal product and is considered related to investigational medicinal product, then investigational medicinal product should be permanently discontinued, and the subject managed according to local practice. Recurrence of laboratory abnormalities considered unrelated to investigational medicinal product may not require permanent discontinuation.

Grade 4 Laboratory Abnormality or Clinical Event

- For a Grade 4 clinical event or clinically significant Grade 4 laboratory abnormality confirmed by repeat testing, the DMC should perform a causality assessment. The study drug may be restarted if the DMC concludes that the AE or laboratory abnormalities were not related to study drug, the AE has resolved, laboratory abnormalities have returned to baseline, and the subject is amenable to close clinical follow-up.
- If a laboratory abnormality recurs to ≥ Grade 4 following re-challenge with investigational medicinal product and is considered related to investigational medicinal product, then investigational medicinal product should be permanently discontinued, and the subject managed according to local practice. Recurrence of laboratory abnormalities considered unrelated to investigational medicinal product may not require permanent discontinuation.
- If, after re-challenge, a subject has a second serious AE or recurrent elevations (even mild or minimal) of total bilirubin, ALT, or AST as defined in Section 6.19, study drug should be discontinued permanently.

6.24. Observation for Drug Induced Liver Injury (DILI)

Throughout the study, all TEAEs, clinical assessments, and clinical laboratory parameters will be closely monitored against the criteria for drug-induced liver injury as detailed in this protocol.

6.24.1. Subjects with Normal Liver Transaminases and Bilirubin at Baseline

Drug-induced liver injury monitoring in subjects with normal liver transaminases and bilirubin at baseline should be performed throughout the study according to the procedures summarized below.

- If subjects with normal baseline liver indices develop elevations of >5 × ULN during the study, repeat testing should be performed within 2 - 5 days from receipt of results. For Cohort C subjects, repeat testing should be performed within 48 to 72 hours from receipt of results.
  - If there are persistent elevations (ALT > 5 × ULN or TBL > 2 × ULN) upon repeat testing, close observation (testing and physical examination 2 to 3 times per week) should be implemented. An important purpose of the close observation is to gather additional clinical information to seek other possible causes of the observed liver test abnormalities, such as one of the following: acute viral hepatitis, alcoholic and autoimmune hepatitis, hepatobiliary disorders, cardiovascular causes, or concomitant treatments. Discontinuation of study drug should be considered.
- Study drug should be discontinued, and the subject should be followed until resolution of signs or symptoms, in the following situations:
  - ALT >8 × ULN
  - ALT >5 × ULN and (TBL >2 × ULN)
  - ALT >5 × ULN with the appearance of fatigue, nausea, vomiting, right upper quadrant pain or tenderness, fever, rash, and/or eosinophilia (> 5%)

For any subjects who present with a constellation of syndromes indicative of liver disease as per the Investigator's overall assessment (i.e., fatigue, nausea, vomiting, right upper quadrant pain or tenderness, fever, rash, and/or eosinophilia [> 5%]), perform liver function tests to determine if liver disease is worsening.

Re-initiation of study drug may be considered after consultation with the Medical Monitor.

6.24.2. Subjects with Elevations in Liver Transaminases or Bilirubin at Baseline

Drug-induced liver injury monitoring in subjects with elevations in liver transaminases or bilirubin at baseline should be performed throughout the study according to the procedures summarized below.

- If subjects with abnormal baseline liver indices develop elevations of ALT >3 × baseline or 300 U/L (whichever occurs first) during the study, repeat testing should be performed within 2 - 5 days.

  - If there are persistent elevations (ALT >3 × baseline, or 300 U/L) upon repeat testing, then close observation (testing and physical examination 2 to 3 times per week) should be implemented and discontinuation of study drug should be considered.
- Discontinue the study drug if any of the following occur:
  - ALT increases to >5 × baseline measurements or 400 U/L (whichever occurs first).
  - ALT increase >3 × baseline measurements or 300 U/L (whichever occurs first) AND the increase is accompanied by a concomitant increase in TBL to > 2 × ULN.
  - ALT increase >3 × baseline measurements or 300 U/L (whichever occurs first) with the appearance of fatigue, nausea, vomiting, right upper quadrant pain or tenderness, fever, rash, and/or eosinophilia (> 5%)

For any subjects who present with a constellation of syndromes indicative of liver disease as per the Investigator's overall assessment (i.e., fatigue, nausea, vomiting, right upper quadrant pain or tenderness, fever, rash, and/or eosinophilia [> 5%]), perform liver function tests to determine if liver disease is worsening.

Re-initiation of study drug may be considered after consultation with the Medical Monitor.

6.24.3. Close Observation for Suspected Drug-Induced Liver Injury

For all subjects, close observation for suspected drug-induced liver injury includes the following within 72 hours of suspected drug-induced liver injury:

- Repeating liver enzyme (ALT, AST, and alkaline phosphatase) and serum bilirubin tests 2 or 3 times weekly.
- The frequency of repeat testing can decrease to once a week or less if abnormalities stabilize or the study drug has been discontinued and the subject is asymptomatic.
- Obtaining a more detailed history of symptoms and prior or concurrent diseases.
- Obtaining a history of concomitant drug use (including nonprescription medications and herbal and dietary supplement preparations), alcohol use, recreational drug use, and special diets.
- Ruling out acute viral hepatitis types A, B, C, D, and E; autoimmune or alcoholic hepatitis; hypoxic/ischemic hepatopathy; and biliary tract disease.
- Obtaining a history of exposure to environmental chemical agents.
- Obtaining additional tests to evaluate liver function, as appropriate (e.g., INR, direct bilirubin).
- Considering gastroenterology or hepatology consultations.
- If biochemistries stabilize and the subject is asymptomatic, monitor liver biochemistries once a week until they return to baseline.
- Subjects who live far from study sites can be evaluated locally for history, physical exam, and laboratories, provided results can be communicated promptly to the site investigator.

6.25. Removal of Subjects from the Study

The Investigator may withdraw a subject from the study for any of the following reasons:

- Withdrawal of consent
- Non-compliance as determined by the Investigator
- At the discretion of the Investigator and/or study Medical Monitor and/or the DMC if it is in the best interest of the subject
- Lost to follow-up

At any time, the Investigator can remove a subject from the study drug and/or the study if deemed necessary for subject safety. The Sponsor also reserves the right to terminate the study at any time. All data normally collected at completion of the study must be collected at the time of the subject's early termination or termination of the study.

The Investigator will discuss with the subject or legally acceptable representative(s) the most appropriate way to withdraw to ensure the subject's health. Should a subject (or a legally acceptable representative) request or decide to withdraw consent, all efforts will be made to complete and report the observations as thoroughly as possible up to the date of withdrawal.

7. PLANNED STATISTICAL METHODS

7.1. Endpoints

7.1.1. Efficacy Endpoints

7.1.1.1. Primary Endpoint

The primary endpoint (Main Study Only) is the absolute change from baseline in hepatic fat fraction measured by MRI-PDFF at Week 12

7.1.1.2. Secondary Efficacy Endpoints

Main Study: The secondary endpoints are:

- Absolute change from baseline in hepatic fat fraction measured by MRI-PDFF at Week 22 - 24
- Percent change from baseline in hepatic fat fraction measured by MRI-PDFF at Weeks 12 and 22 - 24
- Responder: subjects who achieved a clinically meaningful relative reduction of at least 30% in liver fat content as measured by MRI-PDFF at Week 12
- Responder based on NAFLD Activity Score (NAS) system: subjects who had a decrease of $\geq$ 2 points in NAS with at least a 1-point reduction in either lobular inflammation or hepatocellular ballooning and with no concurrent worsening of fibrosis stage
- Change from baseline in ALT at Weeks 12, 16 and 20

Cohort C: The secondary endpoints are:

- Change from baseline in liver stiffness as evaluated by FibroScan® at Week 16
- Changes from baseline in non-invasive biomarkers including Pro-C3 at Weeks 12, 16 and 20, and liver fibrosis by ELF™ Test score at Weeks 6 and 16

7.1.1.3. Exploratory Efficacy Endpoints

The exploratory endpoints are:

- Change from baseline in markers of liver injury and function including AST, ALP, GGT, bilirubin, INR, albumin, MELD score, and CP score at Weeks 12, 16 and 20
- Change from baseline in body weight and waist-to-hip ratio at Weeks 12, 16 and 20
- MRI-PDFF based quantitation of hepatic fat (Main Study only)

- Changes from baseline in non-invasive biomarkers including Pro-C3 at Weeks 12, 16 and 20, and liver fibrosis by ELF™ Test score at Weeks 6 and 12 (Main Study Only)
- Change from baseline in bone mineral density of the lumbar spine, femoral neck, and total hip as measured by DXA at Week 16
- The proportion of subjects at Week 22 - 24 (Main Study) and Week 16 (Cohort C) whose fibrosis regresses by ≥ 1-stage according to the NASH CRN classification
- The proportion of subjects at Week 22 - 24 (Main Study) and Week 16 (Cohort C) who had no worsening in NAS with improvement in fibrosis stage
- The proportion of subjects at Week 22 - 24 (Main Study) and Week 16 (Cohort C) who had 2 points improvement in NAS with no worsening in fibrosis stage
- The proportion of subjects at Week 22 - 24 (Main Study) and Week 16 (Cohort C) who had ≥ 1point improvement in NAS, fibrosis, steatosis lobular inflammation or hepatocellular ballooning
- Changes from baseline in HRQoL (SF-36, CLDQ-NAFLD, WPAI) at Weeks 12 and 16
- Change from baseline in lipoprotein profile and markers of lipid metabolism, insulin sensitivity and glycemic control at Weeks 12, 16 and 20
- Change from baseline in body fat distribution as measured by DXA total body composition at Week 16
- Changes from baseline in corrected T1 (cT1), an MRI-based metric of fibro-inflammation at Weeks 12 and 22 - 24 (Main Study only)
- Plasma concentrations of EFX
- The incidence of the decompensated liver disease events including ascites, hepatic encephalopathy (HE), or variceal bleeding (Cohort C Only)

7.1.2. Safety Endpoints

The safety endpoint is safety and tolerability of EFX in subjects with NASH.

Safety analyses include: summaries of extent of exposure, AEs, laboratory evaluations, ECG, and vital sign assessments, body weight, anti-drug antibody (ADA) and neutralizing antibody (NAB) assessments, and concomitant medication usage.

7.2. Analysis Conventions

All individual subject data will be listed as measured. All statistical summaries and analyses will be performed using Statistical Analysis System (SAS®) software (SAS Institute, Cary, North Carolina, USA).

7.3. Analysis Sets

7.3.1. Efficacy

The primary analysis set for efficacy analyses will be the Full Analysis Set (FAS) which includes all subjects who were randomized into the study.

Subjects who receive a dose of study drug other than that to which they were randomized for the entire duration of treatment will be analyzed according to treatment group to which they were randomized.

7.3.2. Safety

The primary analysis set for safety analyses will be the Safety Set which includes all subjects who received at least one dose of study drug. Subjects who received a dose of study drug other than that to which they were randomized for the entire duration of treatment will be analyzed according to the dose of study drug received.

All data collected during treatment plus 30 days after last dose of study drug will be included in the safety summaries.

7.3.2.1. Pharmacokinetics

The PK analysis set will include all randomized subjects who took at least one dose of study drug and for whom concentration data of EFX is available.

7.3.2.2. Biomarkers

The Biomarker Analysis Set will include data from subjects in the Safety Analysis Set who have the necessary baseline and on-study measurements to provide interpretable results for the specific parameters of interest.

7.4. Data Handling Conventions

Missing data can have an impact on the interpretation of the trial data. In general, values for missing data will not be imputed.

Where appropriate, safety data for subjects that did not complete the study will be included in summary statistics. For example, if a subject received study drug, the subject will be included in a summary of AEs according to the treatment received; otherwise, if the subject is not dosed then they will be excluded from the summary. If safety laboratory results for a subject

are missing for any reason at a time point, the subject will be excluded from the calculation of summary statistics for that time point. If the subject is missing a pre-dose value, then the subject will be excluded from the calculation of summary statistics for the pre-dose value and the change from pre-dose values.

Values for missing safety laboratory data will not be imputed; however, a missing baseline result will be replaced with a screening result, if available. If no pre-treatment laboratory value is available, the baseline value will be assumed to be normal (i.e., no grade [Grade 0]) for the summary of graded laboratory abnormalities.

Values for missing vital signs data will not be imputed; however, a missing baseline result will be replaced with a screening result, if available.

Further details of data handling conventions and transformation will be provided in the SAP.

7.5. Demographic Data and Baseline Characteristics

Demographic and baseline measurements will be summarized using standard descriptive methods.

Demographic summaries will include T2D status, sex, race/ethnicity, and age.

Baseline data will include a summary of body weight, height, BMI, waist-to-hip ratio, randomization stratification groups, and other disease characteristic variables.

7.6. Efficacy Analysis

7.6.1. Primary Efficacy Analysis

The primary efficacy variable will be the absolute change from baseline hepatic fat fraction measured by MRI-PDFF at Week 12. Summary statistics (number of subjects, mean, standard deviation, median, minimum, and maximum) at all visits and change from baseline will be provided. The primary efficacy analysis will be analyzed with an analysis of covariance (ANCOVA) model with treatment group and F1 fibrosis score as factors and baseline hepatic fat fraction measured by MRI-PDFF as a covariate. For any subjects in the full analysis set (FAS) with a missing primary efficacy value, the primary efficacy variable will be imputed with multiple imputation method. The specification of the multiple imputation method will be provided in the SAP. The treatment effect of each active dose comparing with placebo will be estimated from the ANCOVA model. LS means, one-sided, 97.5% confidence intervals and one-sided p-values will be calculated. In order to control the Type I error rate inflation caused by multiplicity issues, a step-down fixed hypothesis testing sequence will be used. That is, only high dose is proved to be significantly better than placebo will the next low dose be compared.

7.6.2. Secondary Efficacy Analysis

Main Study:

For the continuous secondary efficacy variables related to hepatic fat fraction, like percent change from baseline in fat fraction measured by MRI-PDFF at week 12, the same ANCOVA model used for primary efficacy variable will be used. For the other continuous secondary efficacy variables, the ANCOVA model with treatment group, baseline hepatic fat fraction (< 15% vs ≥ 15%), and F1 fibrosis score as factors will be used. Normality will be tested for the model residuals. For certain efficacy variables, logarithmic transformation may be performed prior to fitting the ANCOVA model.

For the responder(s) based on MRI-PDFF, logistic regression will be used for the analyses. The analyses will include the treatment group and F1 fibrosis score as factors and baseline hepatic fat fraction measured by MRI-PDFF as a covariate. The missing value will be imputed as a non-responder.

For the responder(s) based on the NAS system, Fisher's exact test will be used for the analyses. Odds ratios, 95% confidence intervals and p-values will be provided. The missing value will be imputed as a non-responder.

Cohort C:

Summary statistics (number of subjects, mean, standard deviation, median, minimum, and maximum) at all visits and change from baseline will be provided. The efficacy variables will be analyzed with an ANCOVA model with treatment group as a factor and baseline value as a covariate. The effect of Type 2 diabetes status will be explored. Normality will be tested for the model residuals.

7.6.3. Exploratory Efficacy Analysis

The same efficacy analyses used for the secondary efficacy variables will be used for the exploratory efficacy variables.

7.7. Safety Analysis

All safety data collected on or after the date that study drugs were first dispensed up to the date of last dose of study drugs plus 30 days will be summarized by treatment group. Data for the follow-up period will be included in data listings.

7.7.1. Extent of Exposure

Data of a subject's extent of exposure to study drug will be generated from the study drugs administration eCRF data. Exposure data will be summarized by treatment group.

7.7.2. Adverse Events

Clinical and laboratory AEs will be coded using the Medical Dictionary for Regulatory Activities (MedDRA). System Organ Class (SOC), High-Level Group Term (HLGT), High-Level Term (HLT), Preferred Term (PT), and Lower-Level Term (LLT) will be attached to the clinical database. AE severity will be graded using the CTCAE.

Events will be summarized on the basis of the date of onset for the event. A TEAE will be defined as one or both of the following:

- Any AEs with an onset date on or after the study drugs start date and no later than 30 days after permanent discontinuation of study drugs
- Any AEs leading to premature discontinuation of study drugs

Summaries (number and percentage of subjects) of TEAEs and SAEs by SOC and PT will be provided by treatment group. Treatment-emergent AEs will also be summarized by relationship to study drugs and severity. In addition, TEAEs leading to premature discontinuation of study drugs and study, and SAEs leading to death will be summarized and listed.

All AEs collected during the course of the study will be presented in data listings with a field for treatment-emergent event (Yes/No).

7.7.3. Laboratory Evaluations

Selected laboratory data will be summarized (n, mean, SD, median, minimum, and maximum) by treatment group and study visit along with the corresponding change from baseline values.

Graded laboratory abnormalities will be defined using the grading scheme in the CTCAE. Grading of laboratory abnormalities for analysis purposes will be defined in the Statistical Analysis Plan.

The incidence of treatment-emergent laboratory abnormalities, defined as values that increase at least one toxicity grade from baseline at any time post baseline up to and including the date of last dose of study drugs plus 30 days, will be summarized by treatment group. If baseline data are missing, then any graded abnormality (i.e., at least a Grade 1) will be considered treatment-emergent.

7.8. Pharmacokinetic Analysis

Plasma concentrations of EFX will be listed and summarized pre-dose and between 48-96 hours post-dose at the timepoints listed in the Schedule of Assessments. Noncompartmental parameters will be listed and summarized descriptively.

7.9. Biomarker Analysis

Descriptive statistics of biomarker change from baseline will be provided at each sampling time by treatment. Point estimates and 95% confidence intervals may be calculated.

Exploratory analyses may also be performed to evaluate the association of individual exploratory biomarkers or combination of biomarkers with clinical measurements and other risk factors.

Additional exploratory analyses that could enhance the understanding of the biological effects and the mechanism of action of EFX may be added in the Biomarker Analysis Plan if necessary.

7.10. Sample Size

Main Study:

Due to the exploratory nature of this study, no formal power calculations were used to determine sample size. The number of subjects was chosen based on clinical experience with other similar proof of concept studies.

With the following assumptions, the power for N = 20 subjects with active treatment is approximately 91% to detect:

- a true, baseline-adjusted, mean decrease from baseline in MRI-PDFF-estimated hepatic fat of 5% compared to placebo,
- with a one-sided t-test for decrease from placebo,
- a 5% significance level,
- 20 subjects receiving placebo, and
- a standard deviation estimated as 5.2% (Sanyal et al. 2019).

Cohort C:

Since the primary objective is to assess the safety and tolerability of EFX in NASH subjects with compensated cirrhosis, the sample size is determined based on clinical considerations, and the treatment comparisons of efficacy measurements are not powered.

7.11. Data Monitoring Committee

An external DMC that consists of two hepatologists, one cardiologist and a statistician will review the progress of the study. The DMC will convene after 20 subjects (approximately 5 per treatment group) have completed the Week 4 assessments in the Main Study. The DMC will convene again after 10 subjects have completed the Week 4 assessments in Cohort C. The DMC will receive all reports of SAEs including potential DILI and liver decompensation events and convene as needed to monitor for safety. The DMC will provide recommendations to Akero whether the nature, frequency, and severity of adverse effects associated with study treatment warrant the early termination of the study in the best interests of the participants, whether the study should continue as planned, or the study should continue with modifications. The DMC may also provide recommendations as needed regarding study design.

The DMC's specific activities will be defined by a mutually agreed charter, which will define the DMC's membership, conduct, and meeting schedule.

While the DMC will be asked to advise Akero regarding future conduct of the study, including possible early study termination, Akero retains final decision-making authority on all aspects of the study.

7.11.1. Stopping Rules

The DMC will be convened based on emerging safety data if any of the following occur:

- One or more subjects develop the same treatment-emergent grade 5 adverse event or lab abnormality (confirmed by repeat testing) deemed related to EFX.
- Two or more subjects develop the same treatment-emergent grade 4 or higher adverse event or lab abnormality (confirmed by repeat testing) deemed related to the same dose level of EFX.
- Three or more subjects develop the same treatment-emergent grade 3 or higher adverse event or lab abnormality (confirmed by repeat testing) deemed related to the same dose level of EFX.

The DMC will assess the causality and clinical relevance of the adverse events or lab abnormalities and recommend to either halt study dosing for all subjects, pause enrollment, or take no action. If enrollment or dosing is paused, it may be resumed (with or without modifications) only after further review of all available safety data and agreement by the appropriate regulatory agencies.

7.12. Adjudication Committee

An external adjudication committee may be established to adjudicate cases that meet protocol-defined criteria for hepatic decompensation events and possible cases of drug-induced liver injury following DMC review and escalation.

8. ADMINISTRATIVE CONSIDERATIONS

8.1. Investigator Responsibilities

8.1.1. Good Clinical Practice

The Investigator will ensure that this study is conducted in accordance with the principles of the Declaration of Helsinki, International Council for Harmonisation (ICH) guidelines, or with the laws and regulations of the country in which the research is conducted, whichever affords the greater protection to the study subject. These standards are consistent with the European Union Clinical Trials Directive 2001/20/EC and Good Clinical Practice Directive 2005/28/EC.

The Investigator will ensure adherence to the basic principles of Good Clinical Practice, as outlined in 21 Code of Federal Regulations (CFR) 312, subpart D, "Responsibilities of Sponsors and Investigators," 21 CFR, part 50, 1998, and 21 CFR, part 56, 1998.

The Investigator and all applicable subinvestigators will comply with 21 CFR, Part 54, 1998, providing documentation of their financial interest or arrangements with Akero, or proprietary interests in the investigational drug under study. This documentation must be provided prior to the Investigator's (and any subinvestigator's) participation in the study. The Investigator and subinvestigator agree to notify Akero of any change in reportable interests during the study and for 1 year following completion of the study. Study completion is defined as the date when the last subject completes the protocol-defined activities.

8.1.2. Institutional Review Board (IRB)/Independent Ethics Committee (IEC)/ Ethics Committee (EC) Review and Approval

Before initiating a trial, the Investigator (or Sponsor as appropriate according to local regulations) will submit this protocol, ICF, and any accompanying material to be provided to the subject (such as advertisements, subject information sheets, or descriptions of the study used to obtain informed consent) to an IRB/IEC/EC. The Investigator will not begin any study subject activities until approval from the IRB/IEC/EC has been documented and provided as a letter to the Investigator.

Before implementation, the investigator will submit to and receive documented approval from the IRB/IEC/EC any modifications made to the protocol or any accompanying material to be provided to the subject after initial IRB/IEC/EC approval, with the exception of those necessary to reduce immediate risk to study subjects.

8.1.3. Informed Consent

Eligible subjects may only be included in the study after providing (witnessed, where required by law or regulation), IRB/IEC-approved informed consent, or, if applicable after such consent has been provided by a legally acceptable representative(s) of the subject. Informed consent must be obtained before conducting any study-specific procedures (e.g., all

of the procedures described in the protocol). The process of obtaining informed consent must be documented in the subject source documents.

The investigator must use the most current IRB/IEC/EC-approved consent form for documenting written informed consent.

8.1.4. Confidentiality

The investigator will ensure that subjects' anonymity will be strictly maintained and that their identities are protected from unauthorized parties. Only partial date of birth (as applicable in certain countries), another unique identifier (as allowed by local law), and an identification code will be recorded on any form or biological sample submitted to the Sponsor, IRB/IEC/EC, or laboratory. Laboratory specimens must be labeled in such a way as to protect subject identity while allowing the results to be recorded to the proper subject. Refer to specific laboratory instructions. The investigator will keep a screening log showing codes, names, and addresses for all subjects screened and for all subjects enrolled in the trial. Subject data will be processed in accordance with all applicable regulations.

The investigator agrees that all information received from Akero, including but not limited to the IB, this protocol, eCRFs, the IMP, and any other study information, remain the sole and exclusive property of Akero during the conduct of the study and thereafter. This information is not to be disclosed to any third party (except employees or agents directly involved in the conduct of the study or as required by law) without prior written consent from Akero. The investigator further agrees to take all reasonable precautions to prevent the disclosure by any employee or agent of the study site to any third party or otherwise into the public domain.

8.1.5. Study Files and Retention of Records

The investigator must maintain adequate and accurate records to enable the conduct of the study to be fully documented and the study data to be subsequently verified. These documents should be classified into at least the following two categories: (1) Investigator's study file, and (2) subject clinical source documents.

The investigator's study file will contain the protocol/amendments, CRF and query forms, IRB/IEC/EC and governmental approval with correspondence, informed consent, drug records, staff curriculum vitae and authorization forms, and other appropriate documents and correspondence.

The Investigator must maintain source documents for each subject in the study, consisting of case and visit notes (hospital or clinic medical records) containing demographic and medical information, laboratory data, electrocardiograms, and the results of any other tests or assessments. All information on CRFs must be traceable to these source documents in the subject's file. Data not requiring a separate written record will be defined before study start and will be recorded directly on the CRFs. The Investigator must also keep the original informed consent form signed by the subject (a signed copy is given to the subject).

The Investigator must give the monitor access to all relevant source documents to confirm their consistency with the data capture and/or data entry. Akero monitoring standards require full verification for the presence of informed consent, adherence to the inclusion/exclusion criteria, documentation of SAEs, and of data that will be used for all primary variables. Additional checks of the consistency of the source data with the CRFs are performed according to the study-specific monitoring plan. No information in source documents about the identity of the subjects will be disclosed.

All clinical study documents must be retained by the investigator until at least 2 years or according to local laws, whichever is longer, after the last approval of a marketing application in an ICH region (i.e., United States, Europe, or Japan) and until there are no pending or planned marketing applications in an ICH region; or, if no application is filed or if the application is not approved for such indication, until 2 years after the investigation is discontinued and regulatory authorities have been notified. Investigators may be required to retain documents longer if specified by regulatory requirements, by local regulations, or by an agreement with Akero. The investigator must notify Akero before destroying any clinical study records.

Should the investigator wish to assign the study records to another party or move them to another location, Akero must be notified in advance.

If the investigator cannot provide for this archiving requirement at the study site for any or all of the documents, special arrangements must be made between the investigator and Akero to store these records securely away from the site so that they can be returned sealed to the investigator in case of an inspection. When source documents are required for the continued care of the subject, appropriate copies should be made for storage away from the site.

8.1.6. Electronic Case Report Forms

Designated investigator staff will enter the data required by the protocol into the eCRF using fully validated secure web-enabled software that conforms to US CFR 21 Part 11 requirements. Designated investigator site staff will not be given access to the system until they have been trained. Subsequent to data entry, a study monitor will perform source data verification within the EDC system. Original entries as well as any changes to data fields will be stored in the audit trail of the system.

Automatic validation procedures within the system check for data discrepancies during and after data entry and, by generating appropriate error messages, allow the data to be confirmed or corrected online by the designated investigator site staff. The Investigator must certify that the data entered into the electronic Case Report Forms are complete and accurate.

At the conclusion of the trial, Akero will provide the site with a read-only archive copy of the data entered by that site. This archive must be stored in accordance with the records retention requirements outlined in Section 8.1.5.

8.1.7. Inspections

The investigator will make available all source documents and other records for this trial to Akero's appointed study monitors, to IRB/IEC/EC, or to regulatory authority or health authority inspectors.

8.1.8. Protocol Compliance

The investigator is responsible for ensuring the study is conducted in accordance with the procedures and evaluations described in this protocol.

8.2. Sponsor Responsibilities

8.2.1. Protocol Modifications

Protocol modifications, except those intended to reduce immediate risk to study subjects, may be made only by Akero. The investigator must submit all protocol modifications to the IRB/IEC/EC in accordance with local requirements and receive documented IRB/IEC/EC approval before modifications can be implemented.

8.2.2. Study Report and Publications

A clinical study report (CSR) will be prepared and provided to the regulatory agencies. Akero will ensure that the report meets the standards set out in the ICH Guideline for Structure and Content of Clinical Study Reports (ICH E3). Note that an abbreviated report may be prepared in certain cases.

Investigators in this study may communicate, orally present, or publish in scientific journals or other scholarly media only after the following conditions have been met: the results of the study in their entirety have been publicly disclosed by or with the consent of Akero in an abstract, manuscript, or presentation form or the study has been completed at all study sites for at least 18 months.

The investigator will submit to Akero any proposed publication or presentation along with the respective scientific journal or presentation forum at least 60 days before submission of the publication or presentation.

No such communication, presentation, or publication will include Akero's confidential information (see Section 8.1.4).

The investigator will comply with Akero's request to delete references to its confidential information (other than the study results) in any paper or presentation and agrees to withhold publication or presentation for an additional 60 days in order to obtain patent protection if deemed necessary.

8.3. Joint Investigator/Sponsor Responsibilities

8.3.1. Payment Reporting

Investigators and their study staff may be asked to provide services performed under this protocol (e.g., attendance at Investigator's Meetings). If required under the applicable statutory and regulatory requirements, Akero will capture and disclose to Federal and State agencies any expenses paid or reimbursed for such services, including any clinical trial payments, meal, travel expenses or reimbursements, consulting fees, and any other transfer of value.

8.3.2. Access to Information for Auditing or Inspections

Representatives of regulatory authorities or of Akero may conduct inspections or audits of the clinical study. If the investigator is notified of an inspection by a regulatory authority the investigator agrees to notify the Akero medical monitor immediately. The investigator agrees to provide to representatives of a regulatory agency or Akero access to records, facilities, and personnel for the effective conduct of any inspection or audit.

8.3.3. Study Discontinuation

Both the sponsor and the investigator reserve the right to terminate the study at any time. Should this be necessary, both parties will arrange discontinuation procedures and notify the appropriate regulatory authorities, IRBs, IECs, and ECs. In terminating the study, Akero and the investigator will assure that adequate consideration is given to the protection of the subjects' interests.

9. REFERENCE LIST

Adams AC, Halstead CA, Hansen BC, et al. LY2405319, an Engineered FGF21 Variant, Improves the Metabolic Status of Diabetic Monkeys. PLoS One. 2013; 8(6): e65763. doi: 10.1371/journal.pone.0065763.

Bao L, Yin J, Gao W, et al. A long-acting FGF21 alleviates hepatic steatosis and inflammation in a mouse model of non-alcoholic steatohepatitis partly through an FGF21-adiponectin-IL17A pathway. Br J Pharmacol. 2018; 175: 3379-3393.

Cameron JL. Nutritional determinants of puberty. Nutr Rev. 1996; 54(2 Pt 2): S17-22.

Camporez JP, Jornayvaz FR, Petersen MC, et al. Cellular mechanisms by which FGF21 improves insulin sensitivity in male mice. Endocrinology. 2013; 154: 3099–3109.

Chen MS, Chang JC, Zavala-Solorio J, et al. FGF21 mimetic antibody stimulates UCP1-independent brown fat thermogenesis via FGFR1/bKlotho complex in non-adipocytes. Mol. Metab. 2017; 6: 1454-1467.

Coskun T, Bina HA, Schneider MA, et al. Fibroblast growth factor 21 corrects obesity in mice. Endocrinology. 2008; 149: 6018–6027.

Ekstedt M, Hagström H, Nasr P, et al. Fibrosis stage is the strongest predictor for disease-specific mortality in NAFLD after up to 33 years of follow-up. Hepatology. 2015; 61: 1547-54.

Fisher FM, Chui PC, Nasser IA, et al. Fibroblast growth factor 21 limits lipotoxicity by promoting hepatic fatty acid activation in mice on methionine and choline-deficient diets. Gastroenterology. 2014; 147: 1073-83.

Fon Tacer K, Bookout AL, Ding X, et al. Research resource: Comprehensive expression atlas of the fibroblast growth factor system in adult mouse. Mol Endocrinol. 2010; 24: 2050-2064.

Frazier KS, Obert LA. Drug-Induced Glomerulonephritis: The Spectre of Biotherapeutic and Antisense Oligonucleotide Immune Activation in the Kidney. Toxicol Pathol. 2018; 46(8):904-17.

Gaich G, Chien JY, Fu H, et al. The effects of LY2405319, and FGF21 analog, in obese human subjects with type 2 diabetes. Cell Metab. 2013; 18(3):333-340.

Gimeno RE, Moller DE. FGF21-based pharmacotherapy: Potential utility for metabolic disorders. Trends Endocrinol. Metabol. 2014; 25: 303-311.

Harrison SA, Rossi SJ, Paredes AH, Trotter JF, Bashir MR, Guy CD, et al. NGM282 Improves Liver Fibrosis and Histology in 12 Weeks in Patients with Nonalcoholic Steatohepatitis. Hepatology. 2019. doi: 10.1002/hep.30590.

Kim AM, Somayaji VR, Dong JQ, et al. Once-weekly administration of a long-acting fibroblast growth factor 21 analogue modulates lipids, bone turnover markers, blood pressure and body weight differently in obese people with hypertriglyceridaemia and in non-human primates. Diabetes Obes Metab. 2017; 19(12):1762-1772.

Kronenberg S, Husar E, Schubert C, et al. Comparative assessment of immune complex-mediated hypersensitivity reactions with biotherapeutics in the non-human primate: Critical parameters, safety and lessons for future studies. Regul Toxicol Pharmacol. 2017; 88:125-37.

Lee JH, Kang YE, Chang JY, et al. An engineered FGF21 variant, LY2405319, can prevent non-alcoholic steatohepatitis by enhancing hepatic mitochondrial function. Am J Transl Res. 2016; 8: 4750-4763.

Li H, Zhang J, Jia W. Fibroblast growth factor 21: a novel metabolic regulator from pharmacology to physiology. Front Med. 2013; 7: 25–30.

Lin Z, Tian H, Lam KS, et al. Adiponectin mediates the metabolic effects of FGF21 on glucose homeostasis and insulin sensitivity in mice. Cell Metab. 2013; 17: 779–789.

Mu J, Pinkstaff J, Li Z, et al. FGF21 analogs of sustained action enabled by orthogonal biosynthesis demonstrate enhanced antidiabetic pharmacology in rodents. Diabetes. 2012; 61: 505–512.

Nicoles A, Guillet S, Tomlinson E, et al. A mouse model of hepatocellular carcinoma ectopic expression of fibroblast growth factor 19 in skeletal muscle of transgenic mice. Amer. J of Pathology. 2002; 160: 2295-2307.

Nishimura T, Nakatake Y, Konishi M, Itoh N. Identification of a novel FGF, FGF-21, preferentially expressed in the liver. Biochim Biophys Acta. 2000; 1492: 203-206.

Ruhl CE, Everhart JE. Fatty liver indices in the multiethnic United States National Health and Nutrition Examination Survey. Aliment Pharmacol. Ther. 2015; 41: 65–76 .

Sanyal A, Charles ED, Neuschwander-Tetri BA, Loomba R, Harrison SA, Abdelmalek MF, et al. Pegbelfermin (BMS-986036), a PEGylated fibroblast growth factor 21 analogue, in patients with non-alcoholic steatohepatitis: a randomised, double-blind, placebo-controlled, phase 2a trial. Lancet. 2019; 392(10165) :2705-2717.

Shankar S, Bashir MR, Baxter BA, Phung V, Yan AZ, Rossi SJ, et al. NGM313, a Novel Once-Monthly Activator of bKlotho -FGFR1c, Significantly Reduces Hepatic Steatosis and Key Biomarkers of Non-alcoholic Steatohepatitis: Results of a Randomized, Active-Controlled Clamp Study in Obese Insulin-Resistant Patients with NAFLD. Poster presented at American Association for the Study of Liver Diseases, 2018.

Talukdar S, Zhou Y, Li D, et al. A long-acting FGF21 molecule, PF-05231023, decreases body weight and improves lipid profile in non-human primates and type 2 diabetic subjects. Cell Metab. 2016; 23: 427-440.

Tanaka N, Takahashi S, Zhang Y, et al. Role of fibroblast growth factor 21 in the early stage of NASH induced by methionine- and choline-deficient diet. Biochim. Biophysica. Acta. 2015; 1852: 1242-1252.

The NASH Education Program. https://www.the-nash-education-program.com/. Accessed February 25, 2019.

Vahle J. Immunogenicity and immune complex disease in preclinical safety studies. Toxicol Pathol. 2018; 46(8):1013-19.

Wu G, Li H, Fang Q, et al. Complementary role of fibroblast growth factor 21 and cytokeratin 18 in monitoring the different stages of nonalcoholic fatty liver disease. Sci. Rep. 2017; 7: 5095.

Xu J, Lloyd DJ, Hale C, et al. Fibroblast growth factor 21 reverses hepatic steatosis, increases energy expenditure, and improves insulin sensitivity in diet-induced obese mice. Diabetes. 2009; 58: 250–259.

Xu J, Zhang Y, Liu Y, et al. Fibroblast growth factor 21 attenuates hepatic fibrogenesis through TGF-β/smad2/3 and NF-κB signaling pathways. Toxicol Appl. Pharmacol. 2016; 290: 43-53.

Younossi ZM, Koenig AB, Abdelatif D, et al. Global epidemiology of nonalcoholic fatty liver disease- meta-analytic assessment of prevalence, incidence, and outcomes. Hepatology. 2016; 64: 73–84.

10. APPENDICES

Appendix A. Schedule of Assessments- Main Study

Assessments[a]	Screening (- 6 wks)	Pre-Baseline[l] (-2 wks)	Baseline Day 1	On-Treatment Visits[a] Week 1	Wks 2 and 3	Week 4	48-96hrs post-dose	Wks 5, 6 and 7	Week 8	48-96hrs post-dose	Wks 9, 10, 11, 13, 14, 15	Week 12	Week 16	ET	Week 20 Safety follow -up[bb]	Repeat Biopsy Week 22-24[o]
**Clinical Assessments**																
Written Informed Consent	X															
Eligibility	X	X	X													
Medical History	X															
Physical Examination[b]	X		X	X		X			X			X	X	X	X	
Vital Signs[c]	X	X	X	X	X	X	X	X	X	X	X	X	X	X	X	
Weight[w]	X	X	X	X	X	X		X	X		X	X	X	X	X	
Triplicate BP/HR[d]			X				X			X						
Height	X															
Hip and Waist Circumference[w]	X		X	X		X			X			X	X	X	X	
12-lead ECG	X		X										X	X		
FibroScan®	X[r]															
Liver Biopsy	X[e]															X
MRI-PDFF[s]	X[f]							X[f]				X[f]		X[p]		X
DXA Scan[t]	X												X	X		
AUDIT-C Questionnaire	X															
On-Study Questionnaires[g]			X	X		X			X			X	X	X[p]		
Lifestyle Modification[u]	X		X	X	X	X		X	X		X	X	X			
Administer Study Drug[y]			X	X	X	X		X	X		X	X				
Concomitant Medications	X	X	X	X	X	X	X	X	X	X	X	X	X	X	X	X
Adverse Events	X[h]	X	X	X	X	X	X	X	X	X	X	X	X	X	X	X

Assessments[a]	Screening (- 6 wks)	Pre-Baseline[l] (-2 wks)	Baseline Day 1	On-Treatment Visits[a] Week 1	Wks 2 and 3	Week 4	48-96hrs post-dose	Wks 5, 6 and 7	Week 8	48-96hrs post-dose	Wks 9, 10, 11, 13, 14, 15	Week 12	Week 16	ET	Week 20 Safety follow -up[bb]	Repeat Biopsy Week 22-24[o]
**Laboratory Assessments**																
Chemistry, Hematology, Lipids, Coagulation[i]	X	X[x]	X	X	X[z]	X		X[z]	X		X[z]	X	X	X	X	
Serum Storage Sample[q]			X	X		X			X			X	X	X	X	
Urine drug screen[i]	X															
Serologies[i]	X															
Endogenous FGF21[v]			X													
Enhanced Liver Fibrosis (ELF™)	X							X[f]				X[f]		X		
Biomarkers[i]	X[aa]		X	X		X			X			X	X	X	X	
PK[j]			X	X		X	X		X	X		X	X	X	X	X
ADA and NAB[k]			X			X			X				X	X	X	X
Saliva cortisol testing[m]			X	X		X	X		X	X		X	X	X	X	
Pregnancy testing[n]	X		X			X			X			X	X	X	X	

ADA = anti-drug antibodies; AUDIT-C = alcohol use disorders identification test; BP = blood pressure; DXA = Dual Energy X-ray Absorptiometry; ECG = electrocardiogram; HR = heart rate; PK = pharmacokinetic; MRI-PDFF = magnetic resonance imaging – proton density fat fraction; NAB = neutralizing antibody

a All visits after Baseline (Day 1) can be performed within the following window: ± 3 days for weekly visits 1 - 20. Other windows are indicated below.

b Complete PE including assessment at Screening, Baseline and Weeks 12 and 16, symptom driven at weeks 1, 4, 8, 20, or ET. CP Score assessments should be completed as part of the physical exam at Baseline, Week 12, Week 16, ET and Week 20.

c Vitals to include systolic and diastolic BP, pulse, respiration rate, and oral temperature (at Screening and Baseline only) will be collected at each visit.

d Triplicate BP/HR at Baseline and at 48 - 96 hours post-dose on Weeks 4 and 8 to correspond to C_{max}. BP/HR to be measured in same arm with at least two minutes rest between measurements.

e Liver biopsy obtained within 180 days of randomization to confirm eligibility.

f MRI-PDFF and ELF™ will be performed during screening, at Weeks 6 and 12 ± 5 days. Corrected T1 will be collected, where available.

g If the Baseline questionnaires are missed at Day 1, questionnaires should not be collected at the remaining timepoints. The following questionnaires will be administered: HRQoL include: SF-36, CLDQ-NAFLD, WPAI. It is recommended that the questionnaires be completed prior to any study procedures being performed and prior to the subject seeing a health care provider.

h AE reporting during screening is limited to SAEs and AEs related to study procedures.

i Refer to Table 3. At visits where CTX-1 or lipids are collected, the samples to be collected in the morning following an overnight fast of at least 8 hours.

j PK sampling will be obtained pre-dose at all timepoints listed and at 48-96 hours post-dose on weeks 4 and 8 to correspond to C_{max}.
k Samples will be obtained for detection of ADA against EFX and if required a neutralizing ADA assay will be run on the same sample.
l Pre-baseline labs should be collected 4 weeks post-Screening.
m Saliva cortisol testing will be obtained pre-dose at all timepoints listed and at 48 - 96 hours post-dose on Weeks 4 and 8 to correspond to C_{max}.
n For female subjects of childbearing potential only. Serum pregnancy test at screening and urine pregnancy test at other time points.
o Subjects with ≥ 30% relative fat reduction on MRI-PDFF at Week 12 will be required to return at Week 22 - 24 for a repeat liver biopsy and MRI-PDFF. The biopsy and MRI-PDFF should be collected within 5 working days of one another. The window for this visit is – 6 weeks.
p At the discretion of the Investigator.
q Serum storage samples banked for possible additional clinical testing.
r At least 2-3 hours fasting is required prior to all elastography assessments.
s At least 4 hours fasting is required prior to all MR assessments.
t DXA scan should include lumbar spine, femoral neck, and total hip with Subjects in the standard (supine) position. Total body composition may also be collected at the DXA timepoints, where available, to determine body fat distribution.
u All subjects will receive counseling regarding lifestyle modifications including the maintenance of a healthy diet and participation in regular exercise at the Baseline Visit. At each subsequent visit, subjects will be asked if there has been a significant change in diet, or physical activity.
v Endogenous FGF21 serum sample to be collected in the morning following an overnight fast of at least 8 hours.
w Weight, waist and hip circumference should be obtained by the same study personnel between visits for each subject to minimize variability in the measurement. On dosing days, weight will be collected pre-dose. Weight should be obtained using a calibrated scale. The subject should be weighed in consistent clothing.
x Only confirmatory ALT/AST to be collected at the Pre-Baseline Visit to confirm eligibility. Pre-Baseline labs may not be repeated if inclusion criteria is not met.
y Administration of study drug should occur following all other study procedures and sample collection for that visit.
z Glucose only to be measured. Sample should be collected at the end of the study visit.
aa HbA1c only to be measured.
bb The Safety Follow-up visit should be performed 4 weeks following the last dose of study drug. For subjects who complete the study, this will be Week 20. Subjects who terminate the study early should have this visit 4 weeks following the last dose of study drug received.

Appendix B. Schedule of Assessments- Cohort C

Assessments[a]	Screening (- 6 wks)	Pre-Baseline[l] (-2 wks)	Baseline Day 1	On-Treatment Visits[a]											ET	Week 20 Safety follow-up[z]
				Week 1	Wks 2 and 3	Week 4	48-96hrs post-dose	Wks 5, 6 and 7	Week 8	48-96hrs post-dose	Wks 9, 10, 11, 13, 14, 15	Week 12	Week 16			
**Clinical Assessments**																
Written Informed Consent	X															
Eligibility	X	X	X													
Medical History	X															
Physical Examination[b]	X		X	X		X			X			X	X		X	X
Vital Signs[c]	X	X	X	X	X	X	X	X	X	X	X	X	X		X	X
Weight[w]	X	X	X	X	X	X		X	X		X	X	X		X	X
Triplicate BP/HR[d]			X				X			X						
Height	X															
Hip and Waist Circumference[u]	X		X	X		X			X			X	X		X	X
12-lead ECG	X		X										X		X	
FibroScan®	X[q]												X[q]			
Liver Biopsy	X[e]												X[aa]			
DXA Scan[r]	X												X		X	
AUDIT-C Questionnaire	X															
On-Study Questionnaires[g]			X	X		X			X			X	X		X[o]	
Lifestyle Modification[s]	X		X	X	X	X		X	X		X	X	X			
Administer Study Drug[w]			X	X	X	X		X	X		X	X				
Concomitant Medications	X	X	X	X	X	X	X	X	X	X	X	X	X		X	X
Adverse Events	X[h]	X	X	X	X	X	X	X	X	X	X	X	X		X	X
**Laboratory Assessments**																
Chemistry, Hematology, Lipids, Coagulation[i]	X	X[v]	X	X	X[x]	X		X[x]	X		X[x]	X	X		X	X
Serum Storage Sample[p]			X	X		X			X			X	X		X	X

Assessments[a]	Screening (- 6 wks)	Pre-Baseline[l] (-2 wks)	Baseline Day 1	On-Treatment Visits[a]										ET	Week 20 Safety follow-up[z]
				Week 1	Wks 2 and 3	Week 4	48-96hrs post-dose	Wks 5, 6 and 7	Week 8	48-96hrs post-dose	Wks 9, 10, 11, 13, 14, 15	Week 12	Week 16		
Urine drug screen[i]	X														
Serologies[i]	X														
Endogenous FGF21[t]			X												
Enhanced Liver Fibrosis (ELF™)	X							X[f]					X	X	
Biomarkers[i]	X[y]		X	X		X			X			X	X	X	X
PK[j]			X	X		X	X		X	X		X	X	X	X
ADA and NAB[k]			X			X			X				X	X	X
Saliva cortisol testing[m]			X	X		X	X		X	X		X	X	X	X
Pregnancy testing[n]	X		X			X			X			X	X	X	X

ADA = anti-drug antibodies; AUDIT-C = alcohol use disorders identification test; BP = blood pressure; DXA = Dual Energy X-ray Absorptiometry; ECG = electrocardiogram; HR = heart rate; PK = pharmacokinetic; NAB = neutralizing antibody

a All visits after Baseline (Day 1) can be performed within the following window: ± 3 days for weekly visits 1 - 20. Other windows are indicated below.
b Complete PE including assessment at Screening, Baseline and Weeks 12 and 16, symptom driven at weeks 1, 4, 8, 20, or ET. CP Score assessments should be completed as part of the physical exam at Screening, Baseline, Week 12, Week 16, ET and Week 20.
c Vitals to include systolic and diastolic BP, pulse, respiration rate, and oral temperature (at Screening and Baseline only) will be collected at each visit.
d Triplicate BP/HR at Baseline and at 48 - 96 hours post-dose on Weeks 4 and 8 to correspond to C_{max}. BP/HR to be measured in same arm with at least two minutes rest between measurements.
e Historical liver biopsy obtained within 24 months of screening required to confirm eligibility. If a historical biopsy is not available within 24 months of Screening, the subject may undergo a liver biopsy to confirm eligibility.
f ELF™ will be performed during screening, at Weeks 6 and 16 ± 5 days.
g If the Baseline questionnaires are missed at Day 1, questionnaires should not be collected at the remaining timepoints. The following questionnaires will be administered: HRQoL include: SF-36, CLDQ-NAFLD, WPAI. It is recommended that the questionnaires be completed prior to any study procedures being performed and prior to the subject seeing a health care provider.
h AE reporting during screening is limited to SAEs and AEs related to study procedures.
i Refer to Table 3. At visits where CTX-1 or lipids are collected, the samples to be collected in the morning following an overnight fast of at least 8 hours.
j PK sampling will be obtained pre-dose at all timepoints listed and at 48-96 hours post-dose on weeks 4 and 8 to correspond to C_{max}.
k Samples will be obtained for detection of ADA against EFX and if required a neutralizing ADA assay will be run on the same sample.
l Pre-baseline labs should be collected 4 weeks post-Screening.
m Saliva cortisol testing will be obtained pre-dose at all timepoints listed and at 48 - 96 hours post-dose on Weeks 4 and 8 to correspond to C_{max}.
n For female subjects of childbearing potential only. Serum pregnancy test at screening and urine pregnancy test at other time points.
o At the discretion of the Investigator.

p Serum storage samples banked for possible additional clinical testing.
q At least 2-3 hours fasting is required prior to all elastography assessments.
r DXA scan should include lumbar spine, femoral neck, and total hip with Subjects in the standard (supine) position. Total body composition may also be collected at the DXA timepoints, where available, to determine body fat distribution.
s All subjects will receive counseling regarding lifestyle modifications including the maintenance of a healthy diet and participation in regular exercise at the Baseline Visit. At each subsequent visit, subjects will be asked if there has been a significant change in diet, or physical activity.
t Endogenous FGF21 serum sample to be collected in the morning following an overnight fast of at least 8 hours.
u Weight, waist and hip circumference should be obtained by the same study personnel between visits for each subject to minimize variability in the measurement. On dosing days, weight will be collected pre-dose. Weight should be obtained using a calibrated scale. The subject should be weighed in consistent clothing.
v Only confirmatory ALT/AST to be collected at the Pre-Baseline Visit to confirm eligibility. Pre-Baseline labs may not be repeated if inclusion criteria is not met.
w Administration of study drug should occur following all other study procedures and sample collection for that visit.
x Glucose only to be measured. Sample should be collected at the end of the study visit.
y HbA1c only to be measured.
z The Safety Follow-up visit should be performed 4 weeks following the last dose of study drug. For subjects who complete the study, this will be Week 20. Subjects who terminate the study early should have this visit 4 weeks following the last dose of study drug received.
aa Subjects will be given the option to collect an end of treatment biopsy. Biopsy should be taken within 2-4 weeks of last dose of EFX.

Appendix C. Pregnancy Precautions, Definition for Female of Childbearing Potential, and Contraceptive Requirements

1) Definitions

a) Definition of Childbearing Potential

For the purposes of this study, a female born subject is considered of childbearing potential following the initiation of puberty (Tanner stage 2) until becoming post-menopausal, unless permanently sterile or with medically documented ovarian failure.

Women are considered to be in a postmenopausal state when they are ≥ 54 years of age with cessation of previously occurring menses for ≥ 12 months without an alternative cause. In addition, women of any age with amenorrhea of ≥ 12 months may also be considered postmenopausal if their follicle stimulating hormone (FSH) level is in the postmenopausal range and they are not using hormonal contraception or hormonal replacement therapy.

Permanent sterilization includes hysterectomy, bilateral oophorectomy, or bilateral salpingectomy in a female subject of any age.

b) Definition of Male Fertility

For the purposes of this study, a male born subject is considered of fertile after the initiation of puberty unless permanently sterile by bilateral orchidectomy or medical documentation.

2) Contraception Requirements for Female Subjects

a) Study Drug Effects on Pregnancy and Hormonal Contraception

No formal studies have been conducted to evaluate the reproductive toxicity of EFX; therefore, the reproductive toxicity of EFX in humans is unknown.

EFX has not yet been studied in pregnant women.

Please refer to the latest version of the Investigator's Brochure for additional information.

b) Contraception Requirements for Female Subjects of Childbearing Potential

The inclusion of female subjects of childbearing potential requires the use of highly effective contraceptive measures. They must have a negative serum pregnancy test at screening and a negative pregnancy test on the Baseline/Day 1 visit prior to enrollment. Pregnancy tests will be performed at monthly intervals thereafter. Female subjects must agree to one of the following from screening until the Week 20 Safety Follow-up Visit.

- Complete abstinence from intercourse of reproductive potential. Abstinence is an acceptable method of contraception only when it is in line with the subject's preferred and usual lifestyle.

Or

- Consistent and correct use of 1 of the following methods of birth control listed below.
  - Intrauterine device (IUD) with a failure rate of < 1% per year
  - Intrauterine hormone-releasing system (IUS) with a failure rate of < 1% per year
  - Tubal sterilization
  - Essure® micro-insert system (provided confirmation of success 3 months after procedure)
  - Vasectomy in the male partner (provided that the partner is the sole sexual partner and had confirmation of surgical success 3 months after procedure)

Should female subjects wish to use a hormonally based method, use of a male condom by the female subject's male partner is required. Subjects who utilize a hormonal contraceptive as one of their birth control methods must have used the same method for at least three months prior to study dosing. Hormonally-based contraceptives permitted for use in this protocol are as follows:

- Oral contraceptives (either combined or progesterone only)
- Injectable progesterone
- Implants of levonorgestrel
- Transdermal contraceptive patch
- Contraceptive vaginal ring

Not all of these methods may be approved in each of the countries where the study is being conducted: please refer to local product information. Additional local regulatory requirements may apply.

Female subjects must also refrain from egg donation and in vitro fertilization during treatment and until at least 90 days after the last dose of study drug.

3) Contraception Requirements for Male Subjects

It is theoretically possible that a relevant systemic concentration may be achieved in a female partner from exposure of the male subject’s seminal fluid. Therefore, male subjects with female partners of childbearing potential must use condoms during treatment until 90 days after the last dose of study drug. Female partners of male study subjects are asked to select one of the above methods.

Male subjects must also refrain from sperm donation during treatment and until at least 90 days after the last dose of study drug.

4) Unacceptable Birth Control Methods

Birth control methods that are unacceptable include periodic abstinence (e.g., calendar, ovulation, symptothermal, post-ovulation methods), withdrawal (coitus interruptus), spermicides only, and lactational amenorrhea method (LAM). Female condom and male condom should not be used together.

5) Procedures to be Followed in the Event of Pregnancy

Subjects will be instructed to notify the investigator if they become pregnant at any time during the study, or if they become pregnant within 90 days of last study drug dose. Subjects who become pregnant or who suspect that they are pregnant during the study must report the information to the investigator and discontinue study drug immediately. Subjects whose partner has become pregnant or suspects she is pregnant during the study must report the information to the investigator. Instructions for reporting pregnancy, partner pregnancy, and pregnancy outcome are outlined in Section 6.21.5.2.
